# Seleno-Warfare against Cancer: Decoding Antitumor Activity of Novel Acylselenoureas and Se-Acylisoselenoureas

**DOI:** 10.3390/pharmaceutics16020272

**Published:** 2024-02-14

**Authors:** Eduardo Angulo-Elizari, Asif Raza, Ignacio Encío, Arun K. Sharma, Carmen Sanmartín, Daniel Plano

**Affiliations:** 1 Departamento de Ciencias Farmacéuticas, Universidad de Navarra, Irunlarrea 1, 31008 Pamplona, Spain; eangulo.1@alumni.unav.es; 2 Department of Pharmacology, Penn State Cancer Institute, CH72, Penn State College of Medicine, 500 University Drive, Hershey, PA 17033, USA; mraza@pennstatehealth.psu.edu (A.R.); asharma1@pennstatehealth.psu.edu (A.K.S.); 3 Instituto de Investigación Sanitaria de Navarra (IdiSNA), Irunlarrea, 3, 31008 Pamplona, Spain; ignacio.encio@unavarra.es; 4 Departamento de Ciencias de la Salud, Universidad Pública de Navarra, Avda. Barañain s/n, 31008 Pamplona, Spain

**Keywords:** acylselenourea, acylisoselenourea, carbodiimide, synthesis, antiproliferative, cancer, selenium, apoptosis

## Abstract

Currently, cancer remains a global health problem. Despite the existence of several treatments, including chemotherapy, immunotherapy, and radiation therapy, the survival rate for most cancer patients, particularly those with metastasis, remains unsatisfactory. Thus, there is a continuous need to develop novel, effective therapies. In this work, 22 novel molecules containing selenium are reported, including seven Se-acylisoselenoureas synthesized from aliphatic carbodiimides as well as acylselenoureas with the same carbo- and heterocycles and aliphatic amines. After an initial screening at two doses (50 and 10 µM) in MDA-MB-231 (breast), HTB-54 (lung), DU-145 (prostate), and HCT-116 (colon) tumor cell lines, the ten most active compounds were identified. Additionally, these ten hits were also submitted to the DTP program of the NCI to study their cytotoxicity in a panel of 60 cancer cell lines. Compound **4** was identified as the most potent antiproliferative compound. The results obtained showed that compound **4** presented IC_50_ values lower than 10 µM in the cancer cell lines, although it was not the most selective one. Furthermore, compound **4** was found to inhibit cell growth and cause cell death by inducing apoptosis partially via ROS production. Overall, our results suggest that compound **4** could be a potential chemotherapeutic drug for different types of cancer.

## 1. Introduction

Cancer continues to be one of the major health problems worldwide. The incidence, prevalence, and, unfortunately, the number of deaths provoked by this pathology continue increasing year by year. The most frequent tumors based on the incidence are breast, lung, colorectal, and prostate cancer, with more than 1.5 million new cases of each one in 2020 [1]. These high incidence rates translate into high subsequent prevalence and number of deaths in the most common types of tumors. According to the World Health Organization (WHO), the number of patients that died in the year 2020 was 1.79 million from lung cancer, 0.93 million from colorectal cancer, 0.68 million from breast cancer, and 0.37 million from prostate cancer [1]. Cancer includes a group of different diseases originating from diverse genetic and environmental factors. Despite the existence of treatments available, the diversity of cancer types and their heterogenicity, together with the increase in resistance, has emphasized the necessity to develop novel anticancer drugs [2,3].

Selenium (Se), a trace element essential for the correct function of the human organism, is required in small quantities (RDA 55 µg/day). Most of the functions performed by this micronutrient, which can be obtained from the diet, are due to its presence in the amino acid selenocysteine, which is included in selenoproteins. To date, 25 selenoproteins have been characterized. The prolonged deficiency of Se could trigger some pathologies. One of the principal functions of Se is the regulation and maintenance of redox balance and avoiding oxidative stress damage [4,5,6,7,8]. Oxidative stress is related to various diseases because of the damage to macromolecules (membrane lipids, proteins, enzymes, nucleic acids), which harm the cell function, causing its death. In cancer cells, there is an imbalance in the redox homeostasis and the tumor microenvironment [9,10,11]. Endogenous antioxidant enzymes (glutathione peroxidase, thioredoxin reductase, superoxide dismutase), which are selenoproteins, could prevent cancer development [12,13]. Se-containing molecules have exhibited a wide variety of mechanisms of action depending on the type of tumor and the chemical structure. These mechanisms of action include the stimulation of DNA repair, modulation of angiogenesis and cell migration, different types of regulated cell death (apoptosis, autophagy, entosis, mitotic catastrophe, etc.), and modulation of the activity of certain kinases, among other representative ones [14,15]. The antitumor activity of a (single) selenocompound depends on its chemical form and dose. Additionally, (most of the time) multiple mechanisms of action can be triggered by one selenocompound depending on the type of cancer cells or even on the same cancer cell line [16]. Among all of the types of species, organic molecules containing Se have the advantage of being less toxic and more active than inorganic molecules. Compounds such as methylseleninic acid [17], methylselenocysteine [18,19], and selenomethionine [20,21] have been extensively studied, and their anticancer activity in vitro has been demonstrated. Other functional groups with Se are continuously being reported as cytotoxic for cancerous cell lines, including selenocyanates [22,23], selenoesters [24,25], selenides and diselenides [26,27], selenoureas [28,29,30], isoselenocyanates [31,32], and heterocyclic selenazo compounds [33,34] among others [35,36,37,38,39]. The unique properties of this element contained in molecules have emerged as a promising line of research.

In this work, as a continuation of our previous research, a library of novel acylselenoureas was designed together with their Se-acylisoselenoureas analogs [40,41]. The reason to design both libraries of compounds is to evaluate the impact of the exchange on nitrogen (N) and Se atoms in the molecules. Hence, seven Se-acylisoselenoureas were synthesized to compare their biological activity with the corresponding acylselenoureas. A total of 22 new compounds were synthesized, characterized, and purified. The antitumor activity of the compounds was evaluated in four different tumor cell lines derived from breast (MDA-MB-231), lung (HTB-54), colon (HCT-116), and prostate (DU-145) using the colorimetric assay of 3-(4,5-dimethylthiazol-2-yl)-2,5-diphenyltetrazolium bromide (MTT). The ability to induce apoptosis in DU-145, along with the measurement of total radical oxygen species (ROS) levels, were determined.

## 2. Materials and Methods

### 2.1. Chemistry

#### 2.1.1. General Information

Chemical reagents and solvents were purchased from commercial suppliers, including Sigma Aldrich (Merck, Darmstadt, Germany) and Acros Organics (Thermo Scientific, Waltham, MA, USA), and were used as received. Reaction courses were monitored by thin-layer chromatography (TLC) on precoated silica gel 60 UV_254_ aluminum sheets (Merck, Darmstadt, Germany), and the spots were visualized under ultraviolet (UV) light (254 nm). Synthesized compounds were purified by a chromatography column of silica gel 60 Å (0.040–0.063 mm) (Merck, Darmstadt, Germany) with hexane/ethyl acetate as elution solvent. Melting points were determined using a Mettler Toledo FP82 + FP80 apparatus (Mettler Toledo, Greifensee, Switzerland). The structural characterization was made by ^1^H-,^13^C-, ^77^Se-Nuclear Magnetic Resonance (NMR) spectra recorded on a Bruker Avance Neo 400 MHz in CDCl_3_ and DMSO-*d*_6_ operating at 400, 100, and 76 MHz, respectively. Chemical shifts were reported in δ values (ppm), and *J* values were reported in hertz (Hz).

#### 2.1.2. General Procedure of Chlorination of Carboxylic Acids

It was necessary to obtain some acid chloride reagents. For this purpose, these compounds were synthesized by treatment of the corresponding carboxylic acid with an excess of thionyl chloride (20 mL) at reflux for 2 h. The novel acid chloride was isolated by the rotary evaporation of the thionyl chloride under vacuum, and the excess of thionyl chloride was removed by adding three fractions of methylene chloride (3 × 50 mL). The resulting acid chlorides were used without further purification.

#### 2.1.3. General Procedure for the Preparation of Se-Acylisoselenourea Derivatives

To a mixture of elemental selenium (7.62 mmol, 0.60 g) in distilled water (20 mL), sodium borohydride (NaBH_4_) (15.24 mmol, 0.57 g) was added, and the mixture was stirred for 15 min at room temperature. The corresponding carbodiimide (7.62 mmol, 1.5 g) was added in situ and stirred for 30–60 min. Tetrahydrofuran (5 mL) was added dropwise (THF) to the reaction in order to dissolve carbodiimide in the reaction mixture. After the formation of the corresponding selenate, acid chloride (7.62 mmol) was added in situ and stirred at room temperature for 120 min. Another 5 mL of THF can be added again to dissolve acid chloride. The product was isolated by vacuum filtration, and the final product was purified by column chromatography using a gradient of hexane/ethyl acetate as an eluent solvent.

#### 2.1.4. General Procedure for the Preparation of Acylselenourea Derivatives

Fifteen novel acylselenoureas were synthesized following the protocol previously described by our research group [41]. The corresponding acid chlorides (1 equivalent) were reacted with potassium selenocyanate (1 equivalent) in anhydrous acetone (30 mL) as a solvent for 30 min at room temperature. Then, the corresponding amines (1 equivalent) were added and stirred for 2 h. The resulting crude mixture was filtered under vacuum, and the filtrate was evaporated at reduced pressure. The obtained crude was purified by column chromatography using hexane/ethyl acetate as the mobile phase.

The characterization of all 22 novel *Se*-acylisoselenoureas and acylselenoureas is included in the Supplementary Material.

### 2.2. Biological Evaluation

#### 2.2.1. Cell Culture Conditions

The cell lines were purchased from the American Type Culture Collection (ATCC). The four tumor cell lines used were as follows: MDA-MB-231 (triple-negative breast cancer), HTB-54 (lung cancer), DU-145 (prostate cancer), and HCT-116 (colon cancer), and the non-tumoral cell line 185-B5 (mammary tissue) were grown in Roswell Park Memorial Institute (RPMI) 1640 medium culture (Thermo Scientific, USA), supplemented with 10% fetal bovine serum (FBS) (Thermo Scientific, USA) and 1% of antibiotics (10,000 units/mL penicillin and 10 mg/mL streptomycin (Thermo Scientific, USA)). Cells were preserved in tissue culture flasks at 37 ºC and 5% CO_2_. The culture medium was replaced every two to three days.

#### 2.2.2. Cell Viability Assay

Cell viability was determined using the MTT colorimetric assay [42]. A total of 10,000 cells were seeded in each well in a 96-well plate for 24 h. Then, cells were incubated with the corresponding concentration of each tested compound and with DMSO (1%) as control for 48 h. Each compound was dissolved in DMSO at a stock concentration of 0.01 M. Firstly, each compound was tested at 2 concentrations (10 µM and 50 µM) in four human tumoral cell lines MDA-MB-231, HTB-54, DU-145, and HCT-116. Serial dilutions from stock concentration with culture medium were prepared. The most active compounds, those showing cell viability damage in two out of the four cancer cell lines at 10 µM, were further evaluated at seven different concentrations comprised between 1 and 100 µM in the three most active tumor cell lines (MDA-MB-231, HTB-54, and DU-145) and the non-malignant 184-B5 cells. The cell viability was determined by the addition of 20 µL of MTT and its incubation for 2.5 h. Later, the culture medium was removed, and 50 µL of DMSO was added to dissolve formazan crystals. The absorbance was measured at 550 nm. Inhibitory concentration 50 (IC_50_) and selectivity index (SI) were calculated. Three different experiments were performed independently.

#### 2.2.3. NCI60 Analysis

The ten compounds with the highest cell growth inhibition activity were submitted to the Developmental Therapeutics Program (DTP) of the National Cancer Institute (NCI). The assay consists of the evaluation of the cytotoxicity by a One-Dose screening at 10^−5^ M against a panel of 60 cancer cell lines of different tumors (leukemia, lung, central nervous system (CNS), melanoma, ovarian, renal, prostate, and breast cancer cell lines). Seven of the ten hit compounds showed potent cytotoxic activity and were selected for the Five-Dose assay (0.01 µM–100 µM) against the same panel containing 60 cancer cell lines. A range of 5000–40,000 cells/well were seeded and cultured in RPMI 1640 containing 5% of FBS and 2 mM *L*-glutamine and were inoculated into 96-well microtiter plates in 100 µL. The microtiter plates are incubated at 37 °C and 5% humidified CO_2_ for 24 h prior to the addition of experimental drugs. After that, an experimental drug was added, and the microtiter was incubated for 48 h.

#### 2.2.4. Apoptosis Assay

The apoptosis assays were conducted using the Muse Caspase-3/7 assay kit and the Annexin V/7-AAD kit (EMD Millipore, Darmstadt, Germany) following the manufacturer’s instructions. Initially, DU-145 cells were seeded in 6-well plates at a density of 1 × 10^5^ cells per well. After 24 h of incubation, the cells received treatment with 2.5 µM and 5 µM of compound **4**. Subsequently, the cells were incubated for 48 h. Upon completion of the treatment period, the cells were harvested using enzyme-free Cell Dissociation Buffer. The samples were then subjected to the appropriate dyes according to the manufacturer’s instructions and analyzed using the Muse Cell Analyzer (Merck Millipore, Darmstadt, Germany).

#### 2.2.5. Western Blotting

Western blot analysis was conducted to evaluate changes in protein expression levels before and after treatment. In brief, DU-145 cells were treated with 5 µM of compound **4**, and upon completing the designated treatment duration, cell lysis was achieved using RIPA buffer (Thermo Scientific, USA), supplemented with a protease inhibitor cocktail (Thermo Scientific, USA). Subsequently, the resulting lysates were chilled on ice for 30 min and then centrifuged at 10,000 rpm for 15 min to remove cellular debris. Total protein concentration was determined using the BCA assay (Thermo Scientific, USA). Equivalent quantities of cell lysates were separated using NuPAGE 4–12% gels (Life Technologies, Carlsbad, CA, USA), followed by electro-transfer onto a PVDF membrane. Various antibodies were employed to investigate protein expression levels, with detection accomplished using the Enhanced Chemiluminescent reagent (Life Technologies, USA). All antibodies utilized in this study were procured from Cell Signaling Technologies (Danvers, MA, USA).

#### 2.2.6. ROS Assay

The ROS assay was conducted using the Muse oxidative stress assay kit (EMD Millipore, Darmstadt, Germany) following the manufacturer’s instructions. The Muse Oxidative Stress Kit employs a specialized reagent to detect ROS within cells. This kit distinguishes between two distinct cell populations: ROS-negative (ROS−) cells, represented by the M1 peak, and ROS-positive (ROS+) cells, depicted by the M2 peak on the graph. The proportions of ROS-negative and ROS-positive cells were quantified using the Muse cell analyzer. The DU-145 cells were treated with 5 μM concentration of compound **4** with or without 5 mM of N-acetylcysteine (NAC) in the serum-enriched medium for 12 and 24 h.

#### 2.2.7. Statistical Analysis

Cell viability results are expressed as the mean ± standard deviation (SD), and experiments were independently performed thrice in triplicates. IC_50_ values were determined using a non-linear curve regression analysis calculated by OriginPro 9.0 software.

A statistical program from GraphPad software was used for all statistical calculations. Each treatment was independently performed thrice in triplicate unless otherwise noted, and the data are reported as the Mean ± SD. Students’ t-tests and ANOVA analyses were used to determine the significance of differences between groups. A significant difference was assumed if *p* < 0.05. Non-linear curve regression analysis was used to assess the IC_50_ values.

## 3. Results and Discussion

### 3.1. Chemical Design

**Figure 1:**
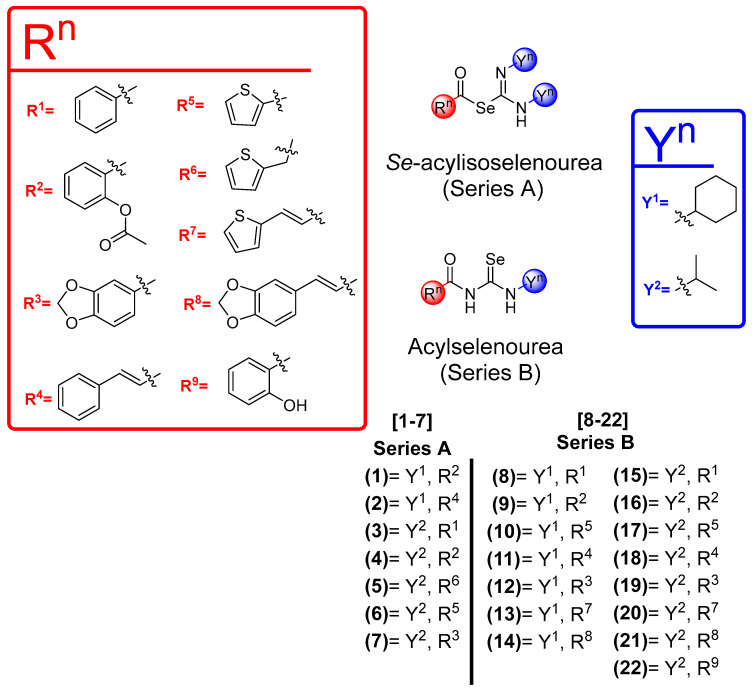
General structures for the novel 22 Se-acylisoselenourea and acylselenourea derivatives synthesized in this study.

**Figure 2:**
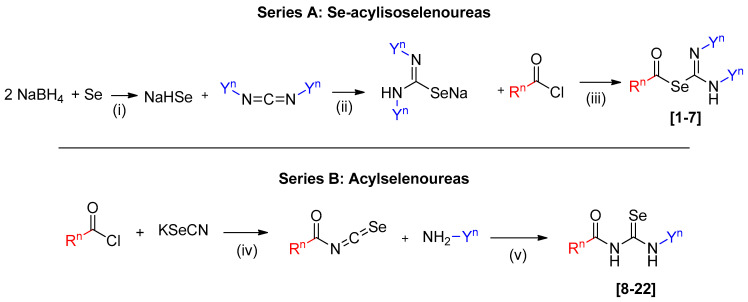
Series (**A**) Synthetic route of Se-acylisoselenoureas (compounds **1–7**). The conditions used were (i) H_2_O, r.t., 15 min; (ii) H_2_O, THF, r.t., 30–60 min; (iii) H_2_O, THF, r.t., 120 min. Series (**B**) Synthetic route of acylselenoureas (compounds **8–22**). The conditions used were (iv) acetone, r.t., 30 min; (v) acetone, r.t., 120 min.

In previous works, our research group has synthesized different acylselenoureas using several aromatic or aliphatic amines, rendering compounds with potent antitumor activity [40,41]. To the best of our knowledge, this work represents the first attempt to exchange the positions of N and Se to evaluate the differences in their biological activity. Additionally, this marks the first occasion where carbodiimides were employed as a fragment to synthesize a new molecule with anticancer activity following the fragment-based design strategy. A library of novel Se-acylisoselenoureas and acylselenoureas containing selenium were grouped into two series: series A, comprising compounds **1–7***,* and series B, encompassing compounds **8–22** (Figure 1), respectively. We considered the use of different small aromatic and heterocyclic rings in the acyl fragment, including thiophene or benzodioxole, with or without a double bond spacer (Figure 1). The synthetic route carried out for the synthesis of the compounds is depicted in Figure 2. The incorporation of heterocyclic structures in molecules with biological activities, including approved drugs, is a common strategy used in medicinal chemistry [43,44,45]. We consider it important to study how the change in N and Se positions affects the biological activity.

### 3.2. Chemistry

The Se-acylisoselenoureas were obtained from the selenated carbodiimide by reaction with different acid chlorides. The complexity in the purification to separate the leftover unreacted carbodiimide diminished the yield, especially in the case of compounds **2**, **5**, and **7**. In contrast, compounds **1**, **3**, **4**, and **6**, with less secondary products in the reaction, presented a moderate yield (20–60%). The mentioned purification issues, especially with cyclohexylcarbodiimide, rendered some products unattainable, leading to their discarding. Few compounds from series A were not obtained because of purification issues of the reaction products (R^1^, R^3^, R^5^, R^6^, R^7^, R^8^, and R^9^ with the cyclohexylcarbodiimide and R^4^, R^8^, and R^9^ with the isopropylacarbodiimide). Further studies into the reaction and purification conditions or the exploration of alternative synthetic routes should be considered in the future. The synthetic procedure of acylselenoureas is also presented (Figure 2). The corresponding acyl isoselenocyanate formed in situ by the reaction of the acid chloride with potassium selenocyanate reacts with the cyclohexylamine or isopropylamine to obtain the corresponding acylselenourea. Similar to Se-acylisoselenoureas, these compounds presented a low to moderate yield. One contributing factor could be the use of aliphatic amines. In previous works of our research group, aliphatic acylselenoureas present much lower yields than aromatic ones [41]. In the case of the aspirin derivative, the hydrolysis of the acetyl group to form the salicylic derivative could be responsible for the lower yields observed in these compounds. The yields for both series of derivatives ranged from 8 to 57%. All the new compounds synthesized in this work were stable at room temperature for at least 6 months. The purity of these compounds was assessed by elemental analysis, and they were characterized by spectroscopic analysis of ^1^H-NMR, ^13^C-NMR, ^77^Se-NMR, and 2D-NMR experiments. These spectra can be found in the supplementary material (Figures S19–S83). The NMR spectra were recorded using different deuterated solvents. Hence, DMSO-*d*_6_ was used for 14 of the compounds due to their poor solubility in CDCl_3_.

In the ^1^H-NMR spectra, hydrogen positions of the aromatic and heteroaromatic rings are similar to several literature data. Regarding the signal peaks corresponding to the CH adjacent to the NH group, slight differences can be found for cyclohexyl and isopropyl moieties. In the case of isopropyl for the anhydride derivatives (series A), the shift appears downfield (ranging from 4.09 to 5.05 ppm) when compared with cyclohexyl (3.49–3.97 ppm). For the amide derivatives (series B), no significant differences were observed for both substituents (ranging from 4.22 to 4.83 ppm).

In the ^13^C-NMR, the observed carbon signals of the compounds were in accordance with the anticipated structures. Interestingly, the signal due to the carbonyl group (C=O) of selenoester (series A) and amide (series B) appears at chemical shifts between 162 and 168 ppm. This signal presented the lowest values for the thiophene substituent in both anhydride (series A) and amide (series B) derivatives. Furthermore, no differences were observed for the C=Se signal in both series A and B, where the signal appears at 178 ppm. Marginal differences were observed for the C=N signal in series A derivatives (compounds **1–7**). All the compounds presented this peak ranging from 185 to 187 ppm, except compound 2, the cinnamic one, which showed the C=N signal at 153 ppm.

The peak for the Se atom appears at different chemical shifts for both structures: *Se*-acylisoselenoureas showed their peak ranged from 450 ppm to 570 ppm, while ^77^Se-NMR shifts of acylselenoureas can be found between 300 and 350 ppm.

### 3.3. Biological Evaluation

#### 3.3.1. Cytotoxic Activity

All the compounds were evaluated in four different cancer cell lines obtained from breast (MDA-MB-231), lung (HTB-54), prostate (DU-145), and colon (HCT-116) at two concentrations (10 and 50 µM). Cell viability was determined after 48 h of treatment through the MTT colorimetric assay. The results are expressed as mean values of the percentage of cell growth from at least three independent experiments carried out in quadruplicates (Figure S1). Most of the compounds presented potent activity in the DU-145 cell line, which proved to be the most sensitive cell line among them. Slightly more moderate activity was shown in MDA-MB-231 and HTB-54 cells. In contrast, each compound presented considerable activity in HCT-116 except for compound **4,** which resulted in the lowest percentage of cell growth in this cell line (36.77%). Those derivatives that produced cell growth inhibition values greater than 50% in at least three cell lines at 10 µM were selected as the lead compounds (**1**, **4**, **8**, **9**, **10**, **12**, **15**, **16**, **17**, and **19**). These lead compounds were further evaluated at seven concentrations comprised between 1 and 100 µM to establish dose-response curves after 48 h of treatment in the MDA-MB-231, HTB-54, and DU-145 cancer cell lines. Interestingly, all compounds presented in their structure hit only four out of the nine different aromatic fragments: phenyl, 2-*O*-acetylphenyl, 1,3-benzodioxole, and thiophene. Additionally, any of the corresponding fragments had an ethylene spacer. In terms of the chemical structure, no differences were exhibited among cyclohexane and isopropane incorporated from their corresponding carbodiimide for series A and amine for series B. No significant differences in the cell growth inhibition activity were observed among different structures in any of the series. The potential selectivity of the compounds was further studied in the cell line derived from normal breast tissue. Results of the dose-response curve are expressed as IC_50_ ± SD values (µM), and the SI is calculated as the ratio of the IC_50_ values of non-malignant cells and tumor cells. The results are compiled in Table 1.

Compound **4** showed the most potent cell growth inhibition with IC_50_ below 7.5 µM toward the three tumoral cell lines. It also exhibited the lowest IC_50_ values in comparison with the rest of the hit compounds in the tumoral cell lines, with the exception of the lung cancer cell line HTB-54, where it was the second most active following compound **16**. Regarding the SI, compound **4** presented a lower selectivity index compared to other lead compounds that were evaluated. Despite its low selectivity, compound **4** was selected to study its mechanism of action further, given its outstanding activity in all three cancer cell lines and its unique chemical structure. The hit compounds obtained from 2-thiophenecarbonyl chloride (compounds **10** and **17**) exhibited the highest SI in the breast cancer cell line (4.83 and 4.15, respectively).

The effect of elemental Se on cell growth has been evaluated in the literature in MDA-MB-231 cells alone and in combination with docetaxel, showing no effect on cell growth when the cells were incubated alone with Se [46]. In contrast, the effect of SeO_2_ and selenium nanoparticles in this breast tumor cancer cell line provokes a diminish in cell viability but maintains at least 50% of cell viability [47]. Other inorganic selenocompounds have been evaluated in the literature in MDA-MB-231, all of them showing IC_50_ values much higher than our synthesized compounds and with similar SIs [48]. On the other hand, the cytotoxicity of elemental Se has not been evaluated in the rest of the cancer cell lines studied herein, although the apoptotic cell death caused by elemental Se in DU-145 has been described [49].

#### 3.3.2. NCI60 Analysis of Compounds **1**, **4**, **8**, **9**, **10**, **12**, **15**, **16**, **17**, and **19**

**Figure 3:**
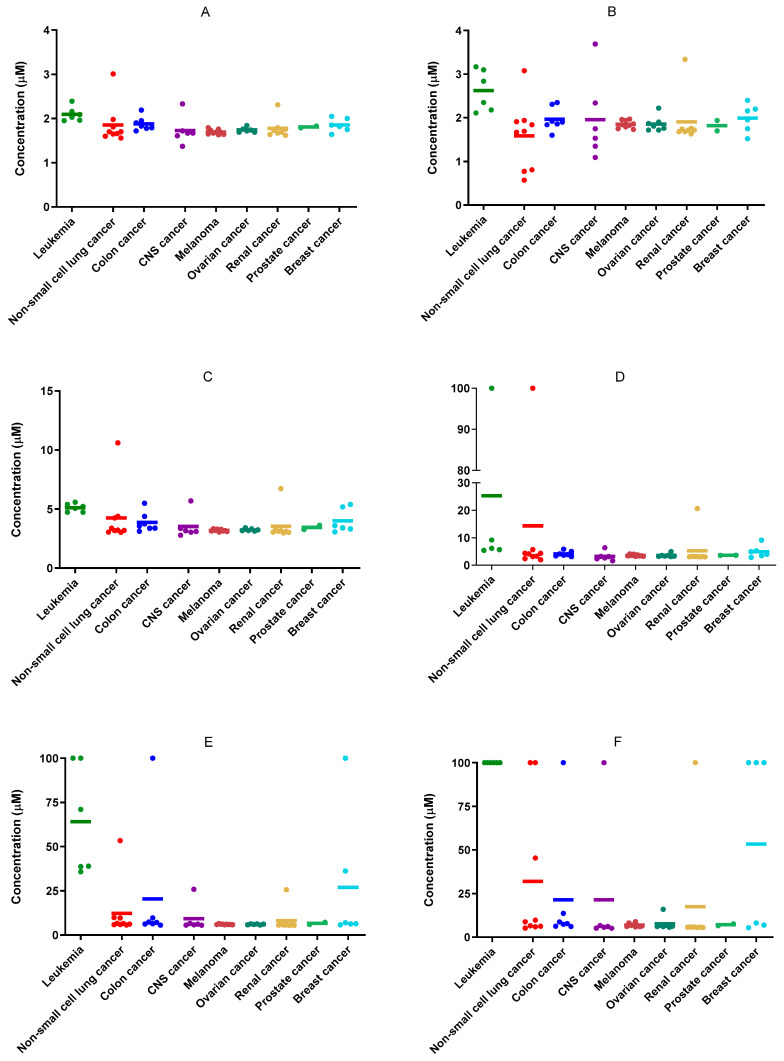
Representation of growth inhibition 50 (GI_50_) (**A**,**B**), total growth inhibition (TGI) (**C**,**D**), and lethal concentration 50 (LC_50_) (**E**,**F**) values for compounds **1** (**A**,**C**,**E**) and **4** (**B**,**D**,**F**) in the panel of 60 cancer cell lines grouped by type of tumor. The representative color lines represent the mean value of each cancer type.

The selected compounds (**1**, **4**, **8**, **9**, **10**, **12**, **15**, **16**, **17**, and **19**) were submitted to the DTP of the NCI. All the compounds, with the exception of **8**, **12**, and **19**, showed outstanding results in the One-Dose assay (1·10^−5^ M) against a panel of 60 cancer cell lines (Figures S2–S11). Compounds **1** and **4** exhibited the most potent activity in this assay, with a mean value of cell growth of −68.50% and −67.93%, respectively. These cell growth mean values represent a significant difference when compared with the third most active compound of the series (**15**), which presented a value of −31.58%. Both compounds (**1** and **4**) present similar cytotoxic behavior in all types of tumor cells. Interestingly, leukemia cells were the least sensitive cells after 48 h of treatment. It should be mentioned that the hit compounds exhibit potent cytotoxicity in the most resistant cell lines of the panel (TK10, SK-MEL-28, SNB-19, SK-OV-3, NCI-H322M, OVCAR-5). Considering these promising results, seven of the ten compounds were selected to perform a Five-Dose assay. Dose-response curves were determined, and the growth inhibition 50 (GI_50_), total growth inhibition (TGI), and lethal concentration 50 (LC_50_) values were calculated for each compound in each cancer cell line. The results are depicted in Figures S12–S18. The seven compounds showed GI_50_ values in the low micromolar range, especially compounds **1**, **4**, and **15**, with GI_50_ values below 2 µM (Figure 3). Once again, the most active compounds, **1** and **4**, showed remarkable inhibition growth capacity in the most resistant cancer cell lines mentioned previously (Figure 3). These results seem to indicate that the combination of aspirin with carbodiimide would achieve hybrid molecules with high antitumor capacity.

#### 3.3.3. Compound **4** Induces Apoptosis in DU-145 Cells

**Figure 4:**
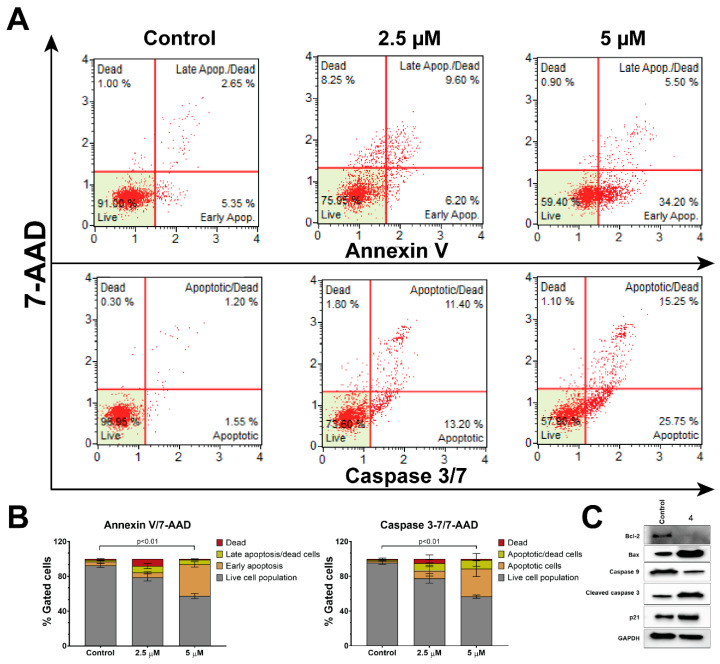
(**A**) DU-145 cells were treated with 2.5 and 5 µM of compound **4** for 48 h, followed by Annexin V/7-AAD assay and Caspase 3/7 assay. (**B**) Quantification of the apoptotic cell population based on the results of the Annexin V/7-AAD assay and Caspase 3/7 assay. The increase in total apoptotic cell population from control to the 5 µM of compound **4** treatment is statistically significant with *p* < 0.01 (**C**) Western blot analysis was conducted to assess the expression of apoptotic proteins in DU-145 cells following treatment with 5 µM of compound **4** for 24 h. The data are presented as mean ± SD, with *n* = 3 for each treatment condition.

DU-145 cells were exposed to varying concentrations of compound **4**, as illustrated in Figure 4, for a 48-h time period. Apoptosis was assessed using the Muse Annexin V & Dead Cell assay kit following the manufacturer’s guidelines.

Cells treated with the vehicle (DMSO) primarily occupied the bottom left quadrant (BLQ), indicating a healthy state (Figure 4). As the concentration of compound **4** increased, cells transitioned from a healthy state to an apoptotic state. At a 2.5 μM concentration of compound **4**, over 25% of cells exhibited signs of apoptosis. With increasing dosage, cells shifted from the bottom right quadrant (BRQ) to the upper right quadrant (URQ). At a 5 μM concentration of compound **4**, more than 50% of cells were identified as apoptotic. Notably, very few cells were classified as necrotic, as there were very few cells detected in the upper left quadrant (ULQ), which would have been 7-AAD positive while remaining Annexin-V and caspase 3/7 negative.

Similarly, compound **4** exhibited a dose-dependent increase in caspase 3/7 activity, as shown in Figure 4. Figure 4B provides the quantification of the total percentage of apoptotic cells, determined through both Annexin-V and caspase 3/7 assays.

Furthermore, we conducted Western blot analysis to examine alterations in apoptotic marker proteins throughout the apoptosis process (Figure 4C). When a cell receives apoptotic signals from external stimuli, pro-caspase-8 undergoes cleavage, and its expression level decreases in the cells. Subsequently, the cleaved caspase-8 activates caspase-3 through direct proteolytic processing or initiates the mitochondrial pathway to apoptosis by facilitating the release of cytochrome c from the mitochondria and the formation of the apoptosome. This, in turn, triggers the activation of caspase-9, leading to the initiation of a cascading process that activates caspases-3 and -7. Bcl-2 protein is believed to prevent Bax from releasing cytochrome c, thus restricting downstream activation of apoptotic machinery. Since a high Bax and/or low Bcl-2, as well as a high Bax/Bcl-2 ratio, favor apoptosis, we analyzed the expression of Bax and Bcl-2 proteins as well.

In our investigation, DU-145 cells treated with compound **4** exhibited a clear activation of the apoptotic pathway, as illustrated in Figure 4C. Treatment with compound **4** resulted in a reduction in the expression of pro-caspase-9, which subsequently increased the cleavage and activation of caspase-3, ultimately leading to heightened apoptosis. Downregulation of Bcl-2 and upregulation of Bax support the progression of apoptosis after the treatment. In summary, our findings conclusively establish that compound **4** hinders the proliferation of prostate cancer cells by inducing apoptosis.

#### 3.3.4. Compound **4** Induced Time-Dependent ROS Production in Prostate Cells

To explore whether the cytotoxic effects induced by compound **4** are linked to ROS production, we assessed total ROS levels in DU-145 cells treated with compound **4**, following a previously established method [50]. DU-145 cells were exposed to compound **4** for 12 and 24 h, and the total ROS levels were quantified.

**Figure 5:**
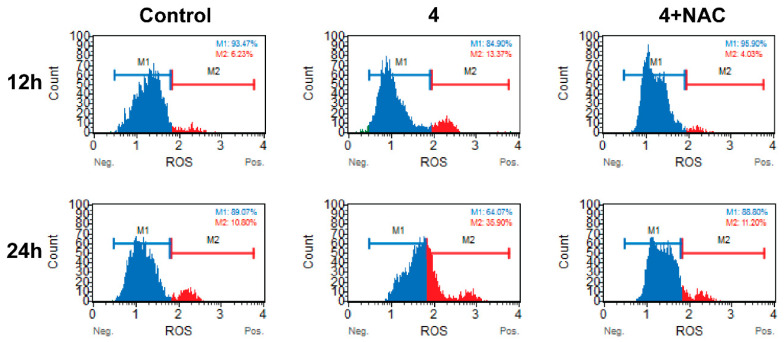
To evaluate the influence of compound **4** on ROS levels, DU-145 cells were exposed to compound **4** for 12 and 24 h. Subsequently, Muse flow cytometry-based oxidative stress analysis was conducted to measure total ROS levels. The histogram reveals the presence of two distinct cell populations: ROS-negative (ROS−) cells (depicted as the M1 peak in blue) and ROS-positive (ROS+) cells (illustrated as the M2 peak in red). Notably, NAC served as a negative control in this analysis.

As depicted in Figure 5, the total ROS levels exhibited a small but significant change following **4**′s treatment during the early time point (12 h). However, after 24 h, there was a notable increase in ROS levels, reaching approximately 35.90% in the cells treated with compound **4**. These findings strongly indicate that compound **4** induces ROS production during the initial stages of treatment. Also, its prolonged exposure continues to support ROS production, which correlates with an increase in apoptosis. This suggests that the induction of cell death by compound **4** may indeed be attributed to ROS production. Nevertheless, further experiments are needed to ensure this pro-oxidant activity is the main cause of the antitumor activity of these derivatives. A great number of reports have demonstrated that pro-oxidant activity is related to the biological effects shown by many selenoderivatives. Recently, a review has discussed this pivotal mechanism and its implications in the pharmacological application of selenocompounds [51].

## 4. Conclusions

In this work, 22 novel selenoderivatives were synthesized through a fragment-based strategy, introducing, for the first time, the use of carbodiimides to obtain *Se*-acylisoselenoureas, which demonstrated potent antiproliferative activity. The combination of *N*-aliphatic amines or carbodiimides together with different carbo- and hetero-cycles resulted in these new acylselenoureas and *Se*-acylisoselenoureas. Our research revealed that altering the position of Se and N presents no significant impact on the antiproliferative activity. In addition, the antitumoral activity of novel compounds was demonstrated in different cancer cell lines. Compound **4** is highlighted as the most potent compound, showing a dose-dependent induction of apoptosis. Moreover, the ROS production by compound **4** suggested a possible mechanism by which this compound may produce cell death. To conclude, the novel *Se*-acylisoselenourea scaffold, together with its acylselenourea analogs, presents the potential to be further developed as a novel therapeutic approach against cancer.

## Figures and Tables

**Table 1:** Inhibitory concentration 50 (IC_50_) values, expressed in micromolar and selectivity index (SI) for compounds **1**, **4**, **8**, **9**, **10**, **12**, **15**, **16**, **17**, and **19** in different human cancer and non-tumoral cell lines.

	Cell Lines				
Compound	Prostate	Lung	Breast	Breast	
	DU-145	HTB-54	MDA-MB-231	184-B5	SI ^a^
	IC_50_ (µM)	IC_50_ (µM)	IC_50_ (µM)	IC_50_ (µM)	
Compound **1**	5.17 ± 0.32	8.31 ± 1.01	8.56 ± 0.10	15.79 ± 1.33	1.84
Compound **4**	5.06 ± 0.02	7.20 ± 1.81	5.48 ± 0.42	9.60 ± 0.97	1.75
Compound **8**	8.83 ± 0.16	14.62 ± 1.11	17.37 ± 5.09	27.02 ± 5.20	1.55
Compound **9**	8.76 ± 2.13	29.82 ± 22.48	10.24 ± 0.29	11.42 ± 18.00	1.11
Compound **10**	5.18 ± 0.19	10.09 ± 4.88	10.14 ± 0.25	48.99 ± 39.17	4.83
Compound **12**	5.22 ± 0.13	10.72 ± 13.75	30.67 ± 2.31	39.32 ± 18.36	1.28
Compound **15**	5.19 ± 1.55	26.35 ± 16.85	24.54 ± 7.55	8.51 ± 4.81	0.34
Compound **16**	11.40 ± 3.07	23.01 ± 23.53	10.21 ± 13.56	22.19 ± 3.41	2.17
Compound **17**	5.12 ± 0.07	5.09 ± 0.34	10.16 ± 0.39	42.23 ± 11.39	4.15
Compound **19**	5.13 ± 0.02	21.92 ± 9.10	12.69 ± 10.80	36.79 ± 14.50	2.89

Inhibitory concentration 50 (IC_50_) values are represented as the mean ± SD of the three independent experiments determined by the MTT assay. ^a^ Selectivity index (SI) is calculated in lung cells as IC_50_ 184-B5/IC_50_ MDA-MB-231, respectively.

## Data Availability

Dataset available on request from the authors.

## Supplementary Materials

The following supporting information can be downloaded at: https://www.mdpi.com/article/10.3390/pharmaceutics16020272/s1, Figure S1–Figure S18: Biological Evaluation, Figure S19–Figure S83: Structural characterization.

## Abbreviations

7-AAD	7-Amino-actinomycin D
ATCC	American Tissue Culture Collection
C	Carbon
DMSO	Dimethyl sulfoxide
DNA	Deoxyribonucleic acid
DTP	Drug Therapeutic Program
FBS	Fetal bovine serum
g	gram
GI_50_	Growth inhibition 50
H	Hydrogen
Hz	Hertz
IC_50_	Inhibitory concentration 50
LC_50_	Lethal concentration 50
mmol	Milimol
MTT	3-(4,5-dimethylthiazol-2-yl)-2,5-diphenyltetrazolium bromide
N	Nitrogen
NAC	N-acetylcysteine
NCI	National Cancer Institute
NMR	Nuclear Magnetic Resonance
r.t.	room temperature
RDA	Recommended Dietary Allowance
ROS	Reactive Oxygen Species
RPMI	Roswell Park Memorial Institute
SD	Standard deviation
Se	Selenium
SI	Selectivity index
TGI	Total growth inhibition
THF	Tetrahydrofuran
TLC	Thin Layer Chromatography
UV	Ultraviolet

## References

[1] Sung H., Ferlay J., Siegel R.L., Laversanne M., Soerjomataram I., Jemal A., Bray F. (2021). Global Cancer Statistics 2020: GLOBOCAN Estimates of Incidence and Mortality Worldwide for 36 Cancers in 185 Countries. CA Cancer J. Clin..

[2] Holohan C., Van Schaeybroeck S., Longley D.B., Johnston P.G. (2013). Cancer drug resistance: An evolving paradigm. Nat. Rev. Cancer.

[3] Maleki E.H., Bahrami A.R., Matin M.M. (2024). Cancer cell cycle heterogeneity as a critical determinant of therapeutic resistance. Genes Dis..

[4] Mehdi Y., Hornick J.L., Istasse L., Dufrasne I. (2013). Selenium in the environment, metabolism and involvement in body functions. Molecules.

[5] Mojadadi A., Au A., Salah W., Witting P., Ahmad G. (2021). Role for Selenium in Metabolic Homeostasis and Human Reproduction. Nutrients.

[6] Roman M., Jitaru P., Barbante C. (2014). Selenium biochemistry and its role for human health. Metallomics.

[7] Zoidis E., Seremelis I., Kontopoulos N., Danezis G.P. (2018). Selenium-Dependent Antioxidant Enzymes: Actions and Properties of Selenoproteins. Antioxidants.

[8] Labunskyy V.M., Hatfield D.L., Gladyshev V.N. (2014). Selenoproteins: Molecular pathways and physiological roles. Physiol. Rev..

[9] Forman H.J., Zhang H. (2021). Targeting oxidative stress in disease: Promise and limitations of antioxidant therapy. Nat. Rev. Drug Discov..

[10] Moldogazieva N.T., Lutsenko S.V., Terentiev A.A. (2018). Reactive Oxygen and Nitrogen Species-Induced Protein Modifications: Implication in Carcinogenesis and Anticancer Therapy. Cancer Res..

[11] Assi M. (2017). The differential role of reactive oxygen species in early and late stages of cancer. Am. J. Physiol.-Regul. Integr. Comp. Physiol..

[12] Davis C.D., Tsuji P.A., Milner J.A. (2012). Selenoproteins and cancer prevention. Annu. Rev. Nutr..

[13] Short S.P., Williams C.S. (2017). Selenoproteins in Tumorigenesis and Cancer Progression. Adv. Cancer Res..

[14] Kuršvietienė L., Mongirdienė A., Bernatonienė J., Šulinskienė J., Stanevičienė I. (2020). Selenium Anticancer Properties and Impact on Cellular Redox Status. Antioxidants.

[15] Radomska D., Czarnomysy R., Radomski D., Bielawski K. (2021). Selenium Compounds as Novel Potential Anticancer Agents. Int. J. Mol. Sci..

[16] Kim S.J., Choi M.C., Park J.M., Chung A.S. (2021). Antitumor Effects of Selenium. Int. J. Mol. Sci..

[17] Varlamova E.G., Turovsky E.A. (2021). The main cytotoxic effects of methylseleninic acid on various cancer cells. Int. J. Mol. Sci..

[18] Lendvai G., Szekerczés T., Kontsek E., Selvam A., Szakos A., Schaff Z., Björnstedt M., Kiss A. (2020). The Effect of Methylselenocysteine and Sodium Selenite Treatment on microRNA Expression in Liver Cancer Cell Lines. Pathol. Oncol. Res..

[19] Lu Z., Qi L., Li G.X., Bo X.J., Liu G.D., Wang J.M. (2015). Se-methylselenocysteine suppresses the growth of prostate cancer cell DU145 through connexin 43-induced apoptosis. J. Cancer Res. Ther..

[20] Korbut E., Ptak-Belowska A., Brzozowski T. (2018). Inhibitory effect of selenomethionine on carcinogenesis in the model of human colorectal cancer in vitro and its link to the Wnt/β-catenin pathway. Acta Biochim. Pol..

[21] Li T., Xiang W., Li F., Xu H. (2018). Self-assembly regulated anticancer activity of platinum coordinated selenomethionine. Biomaterials.

[22] Huang Y., Wei M., Peng Z., Cheng Y., Zhang Y., Li J., Xiao J., Gan C., Cui J. (2022). Synthesis of estrone selenocyanate Compounds, anti-tumor activity evaluation and Structure-activity relationship analysis. Bioorganic Med. Chem..

[23] Huang Y.M., Cheng Y., Peng Z.N., Pang L.P., Li J.Y., Xiao J.A., Zhang Y.F., Cui J.G. (2023). Synthesis and antitumor activity of some cholesterol-based selenocyanate compounds. Steroids.

[24] Csonka A., Kincses A., Nové M., Vadas Z., Sanmartín C., Domínguez-Álvarez E., Spengler G. (2019). Selenoesters and Selenoanhydrides as Novel Agents Against Resistant Breast Cancer. Anticancer Res..

[25] Radomska D., Czarnomysy R., Szymanowska A., Radomski D., Domínguez-Álvarez E., Bielawska A., Bielawski K. (2022). Novel Selenoesters as a Potential Tool in Triple-Negative Breast Cancer Treatment. Cancers.

[26] Álvarez-Pérez M., Ali W., Marć M.A., Handzlik J., Domínguez-Álvarez E. (2018). Selenides and Diselenides: A Review of Their Anticancer and Chemopreventive Activity. Molecules.

[27] Nie Y., Zhong M., Li S., Li X., Zhang Y., Zhang Y., He X. (2020). Synthesis and Potential Anticancer Activity of Some Novel Selenocyanates and Diselenides. Chem. Biodivers..

[28] Nie Y., Li S., Lu Y., Zhong M., Li X., Zhang Y., He X. (2022). New Organoselenium (NSAIDs-Selenourea and Isoselenocyanate) Derivatives as Potential Antiproliferative Agents: Synthesis, Biological Evaluation and in Silico Calculations. Molecules.

[29] Barbosa F.A.R., Siminski T., Canto R.F.S., Almeida G.M., Mota N., Ourique F., Pedrosa R.C., Braga A.L. (2018). Novel pyrimidinicselenourea induces DNA damage, cell cycle arrest, and apoptosis in human breast carcinoma. Eur. J. Med. Chem..

[30] Hussain R.A., Badshah A., Pezzuto J.M., Ahmed N., Kondratyuk T.P., Park E.J. (2015). Ferrocene incorporated selenoureas as anticancer agents. J. Photochem. Photobiol. B.

[31] Frieben E.E., Amin S., Sharma A.K. (2019). Development of Isoselenocyanate Compounds’ Syntheses and Biological Applications. J. Med. Chem..

[32] Hunakova L., Horvathova E., Matuskova M., Bobal P., Otevrel J., Brtko J. (2022). In vitro antiproliferative and cytotoxic activities of novel triphenyltin isoselenocyanate in human breast carcinoma cell lines MCF 7 and MDA-MB-231. Med. Oncol..

[33] Cui J., Pang L., Wei M., Gan C., Liu D., Yuan H., Huang Y. (2018). Synthesis and antiproliferative activity of 17-[1′,2′,3′]-selenadiazolylpregnenolone compounds. Steroids.

[34] Mhaidat N.M., Al-Smadi M., Al-Momani F., Alzoubi K.H., Mansi I., Al-Balas Q. (2015). Synthesis, antimicrobial and in vitro antitumor activities of a series of 1,2,3-thiadiazole and 1,2,3-selenadiazole derivatives. Drug Des. Dev. Ther..

[35] Tang H., Liang Y., Cheng J., Ding K., Wang Y. (2021). Bifunctional chiral selenium-containing 1,4-diarylazetidin-2-ones with potent antitumor activities by disrupting tubulin polymerization and inducing reactive oxygen species production. Eur. J. Med. Chem..

[36] Chuai H., Zhang S.Q., Bai H., Li J., Wang Y., Sun J., Wen E., Zhang J., Xin M. (2021). Small molecule selenium-containing compounds: Recent development and therapeutic applications. Eur. J. Med. Chem..

[37] Da Cruz E.H.G., Silvers M.A., Jardim G.A.M., Resende J.M., Cavalcanti B.C., Bomfim I.S., Pessoa C., de Simone C.A., Botteselle G.V., Braga A.L. (2016). Synthesis and antitumor activity of selenium-containing quinone-based triazoles possessing two redox centres, and their mechanistic insights. Eur. J. Med. Chem..

[38] Gandin V., Khalkar P., Braude J., Fernandes A.P. (2018). Organic selenium compounds as potential chemotherapeutic agents for improved cancer treatment. Free Radic. Biol. Med..

[39] He X., Zhong M., Li S., Li X., Li Y., Li Z., Gao Y., Ding F., Wen D., Lei Y. (2020). Synthesis and biological evaluation of organoselenium (NSAIDs-SeCN and SeCF(3)) derivatives as potential anticancer agents. Eur. J. Med. Chem..

[40] Astrain-Redin N., Raza A., Encío I., Sharma A.K., Plano D., Sanmartín C. (2023). Novel Acylselenourea Derivatives: Dual Molecules with Anticancer and Radical Scavenging Activity. Antioxidants.

[41] Ruberte A.C., Ramos-Inza S., Aydillo C., Talavera I., Encío I., Plano D., Sanmartín C. (2020). Novel N,N’-Disubstituted Acylselenoureas as Potential Antioxidant and Cytotoxic Agents. Antioxidants.

[42] Mosmann T. (1983). Rapid colorimetric assay for cellular growth and survival: Application to proliferation and cytotoxicity assays. J. Immunol. Methods.

[43] Sachdeva H., Khaturia S., Saquib M., Khatik N., Khandelwal A.R., Meena R., Sharma K. (2022). Oxygen- and Sulphur-Containing Heterocyclic Compounds as Potential Anticancer Agents. Appl. Biochem. Biotechnol..

[44] Martins P., Jesus J., Santos S., Raposo L.R., Roma-Rodrigues C., Baptista P.V., Fernandes A.R. (2015). Heterocyclic Anticancer Compounds: Recent Advances and the Paradigm Shift towards the Use of Nanomedicine’s Tool Box. Molecules.

[45] Archna, Pathania S., Chawla P.A. (2020). Thiophene-based derivatives as anticancer agents: An overview on decade’s work. Bioorganic Chem..

[46] Park S.O., Yoo Y.B., Kim Y.H., Baek K.J., Yang J.H., Choi P.C., Lee J.H., Lee K.R., Park K.S. (2015). Effects of combination therapy of docetaxel with selenium on the human breast cancer cell lines MDA-MB-231 and MCF-7. Ann. Surg. Treat. Res..

[47] Luo H., Wang F., Bai Y., Chen T., Zheng W. (2012). Selenium nanoparticles inhibit the growth of HeLa and MDA-MB-231 cells through induction of S phase arrest. Colloids Surf. B Biointerfaces.

[48] Da Costa N.S., Lima L.S., Oliveira F.A.M., Galiciolli M.E.A., Manzano M.I., Garlet Q.I., Irioda A.C., Oliveira C.S. (2023). Antiproliferative Effect of Inorganic and Organic Selenium Compounds in Breast Cell Lines. Biomedicines.

[49] Jiang C., Wang Z., Ganther H., Lu J. (2001). Caspases as key executors of methyl selenium-induced apoptosis (anoikis) of DU-145 prostate cancer cells. Cancer Res..

[50] Plano D., Karelia D.N., Pandey M.K., Spallholz J.E., Amin S., Sharma A.K. (2016). Design, synthesis, and biological evaluation of novel selenium (Se-NSAID) molecules as anticancer agents. J. Med. Chem..

[51] Nogara P.A., Bortoli M., Orian L., Rocha J.B.T. (2022). Biological activity of synthetic organoselenium compounds: What do we know about the mechanim?. Curr. Chem. Biol..

