# Supplementary material for: Seleno-Warfare against Cancer: Decoding Antitumor Activity of Novel Acylselenoureas and Se-Acylisoselenoureas
Source: Pharmaceutics. 2024 Feb 14;16(2):272. doi: 10.3390/pharmaceutics16020272 (PMC10891803; doi:10.3390/pharmaceutics16020272)
Supplement: Supplementary file 1 [file pharmaceutics-16-00272-s001.zip › pharmaceutics-2824925-supplementary.pdf]

Seleno-warfare against cancer: Decoding antitumor activity of novel acylselenoureas and *Se*-acylisoselenoureas.

Eduardo Angulo-Elizari[1], Asif Raza[2], Ignacio Encío[3,4], Arun K. Sharma[2], Carmen Sanmartín[1,3,*], Daniel Plano[1,3,*]

1 Departamento de Ciencias Farmacéuticas, Universidad de Navarra, Irunlarrea 1,31008 Pamplona, Spain; eangulo.1@alumni.unav.es (E.A.E.)
2 Department of Pharmacology, Penn State Cancer Institute, CH72, Penn State College of Medicine, 500 University Drive, Hershey, PA 17033, USA; mraza@pennstatehealth.psu.edu (A.R.); asharma1@pennstatehealth.psu.edu (A.K.S.)
3 Instituto de Investigación Sanitaria de Navarra (IdiSNA), Irunlarrea, 3, 31008 Pamplona, Spain; ignacio.encio@unavarra.es (I.E.)
4 Departamento de Ciencias de la Salud, Universidad Pública de Navarra, Avda. Barañain s/n, 31008 Pamplona, Spain
* Correspondence: sanmartin@unav.es (C.S.); dplano@unav.es (D.P.)

Table of contents

**Biological evaluation**	
Cytotoxicity screening	Figure S1
NCI-60 Analysis	
Compound 1 (One-Dose Assay)	Figure S2
Compound 4 (One-Dose Assay)	Figure S3
Compound 8 (One-Dose Assay)	Figure S4
Compound 9 (One-Dose Assay)	Figure S5
Compound 10 (One-Dose Assay)	Figure S6
Compound 12 (One-Dose Assay)	Figure S7
Compound 15 (One-Dose Assay)	Figure S8
Compound 16 (One-Dose Assay)	Figure S9
Compound 17 (One-Dose Assay)	Figure S10
Compound 19 (One-Dose Assay)	Figure S11
Compound 1 (Five-Dose Assay)	Figure S12
Compound 4 (Five-Dose Assay)	Figure S13
Compound 9 (Five-Dose Assay)	Figure S14
Compound 10 (Five-Dose Assay)	Figure S15
Compound 15 (Five-Dose Assay)	Figure S16
Compound 16 (Five-Dose Assay)	Figure S17
Compound 17 (Five-Dose Assay)	Figure S18
**Structural characterization**	
^{1}H-, ^{13}C- and ^{77}Se-NMR spectra	Figures S19–S83
Characterization of the compounds synthesized in this work	

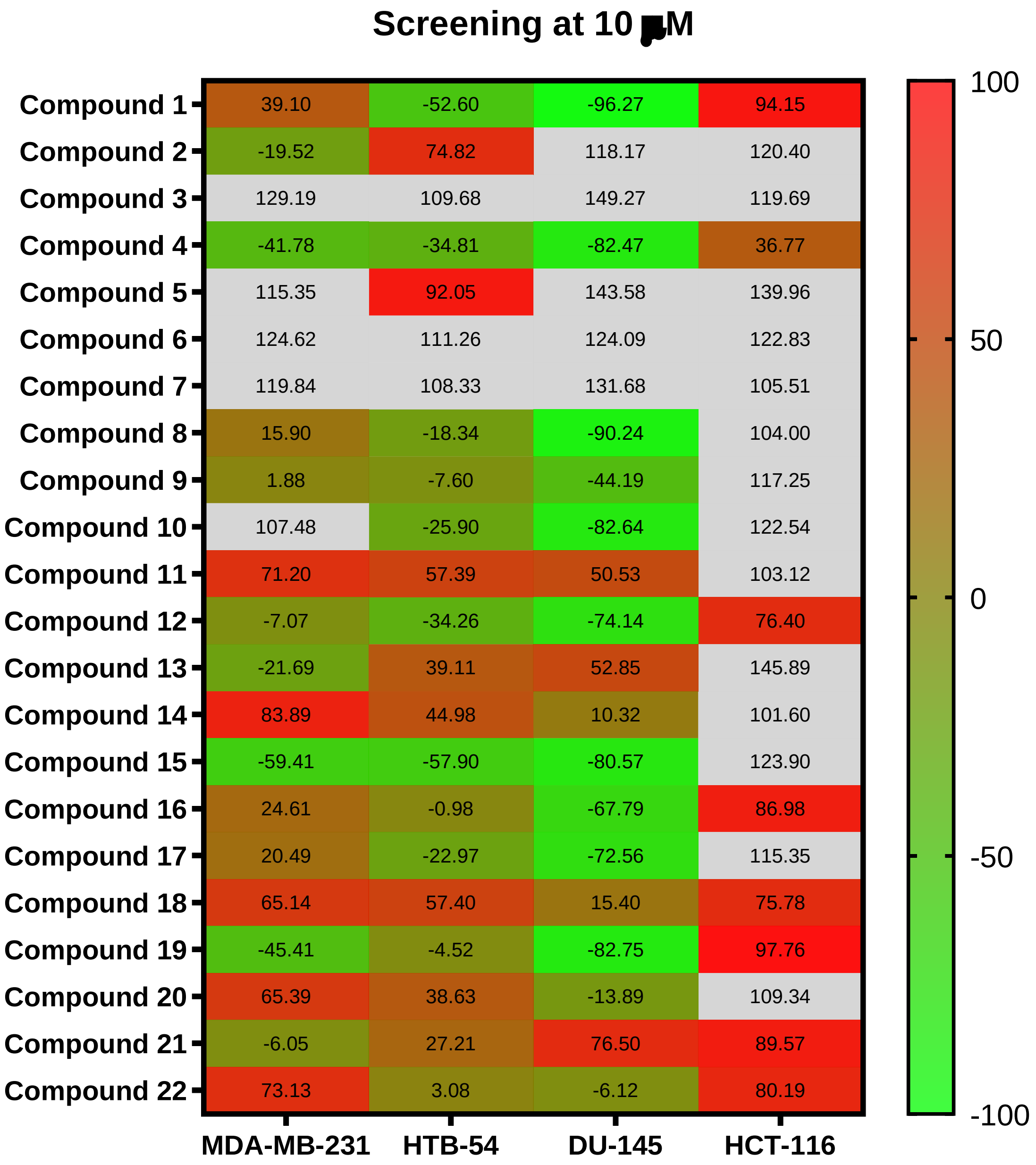

Figure S1. Heat map of screening results where the percentage of cell growth is represented at 10 µM for the 22 novel compounds in MDA-MB-231, HTB-54, DU-145 and HCT-116 cells.

Panel/Cell Line	Growth Percent
Leukemia	
CCRF-CEM	4.94
HL-60(TB)	-17.53
K-562	3.88
MOLT-4	-22.24
RPMI-8226	4.77
SR	-15.65
Non-Small Cell Lung Cancer	
A549/ATCC	0.23
EKVX	-97.62
HOP-62	-87.02
HOP-92	-13.61
NCI-H226	-88.07
NCI-H23	-79.87
NCI-H322M	-82.66
NCI-H460	-41.61
NCI-H522	-89.12
Colon Cancer	
COLO 205	-70.56
HCC-2998	-92.57
HCT-116	-69.88
HCT-15	-73.50
HT29	-60.83
KM12	-67.83
CNS Cancer	
SF-268	8.57
SF-295	-78.91
SF-539	-98.67
SNB-19	-94.90
SNB-75	-63.32
U251	-86.96
Melanoma	
MALME-3M	-91.78
M14	-73.67
MDA-MB-435	-77.20
SK-MEL-2	-74.47
SK-MEL-28	-89.13
SK-MEL-5	-89.30
UACC-257	-89.94
UACC-62	-90.69
Ovarian Cancer	
IGROV1	-94.04
OVCAR-3	-71.59
OVCAR-4	-85.89
OVCAR-5	-89.61
OVCAR-8	-81.78
NCI/ADR-RES	-63.28
SK-OV-3	-99.06
Renal Cancer	
786-0	-93.39
A498	-5.30
ACHN	-73.80
CAKI-1	-97.65
RXF 393	-99.72
SN12C	-99.32
TK-10	-98.82
UO-31	-100.00
Prostate Cancer	
PC-3	-31.01
DU-145	-79.99
Breast Cancer	
MCF7	-82.09
MDA-MB-231/ATCC	-98.47
BT-549	-91.68
T-47D	-43.31
MDA-MB-468	-77.84
Mean	-68.50
Delta	31.50
Range	108.57

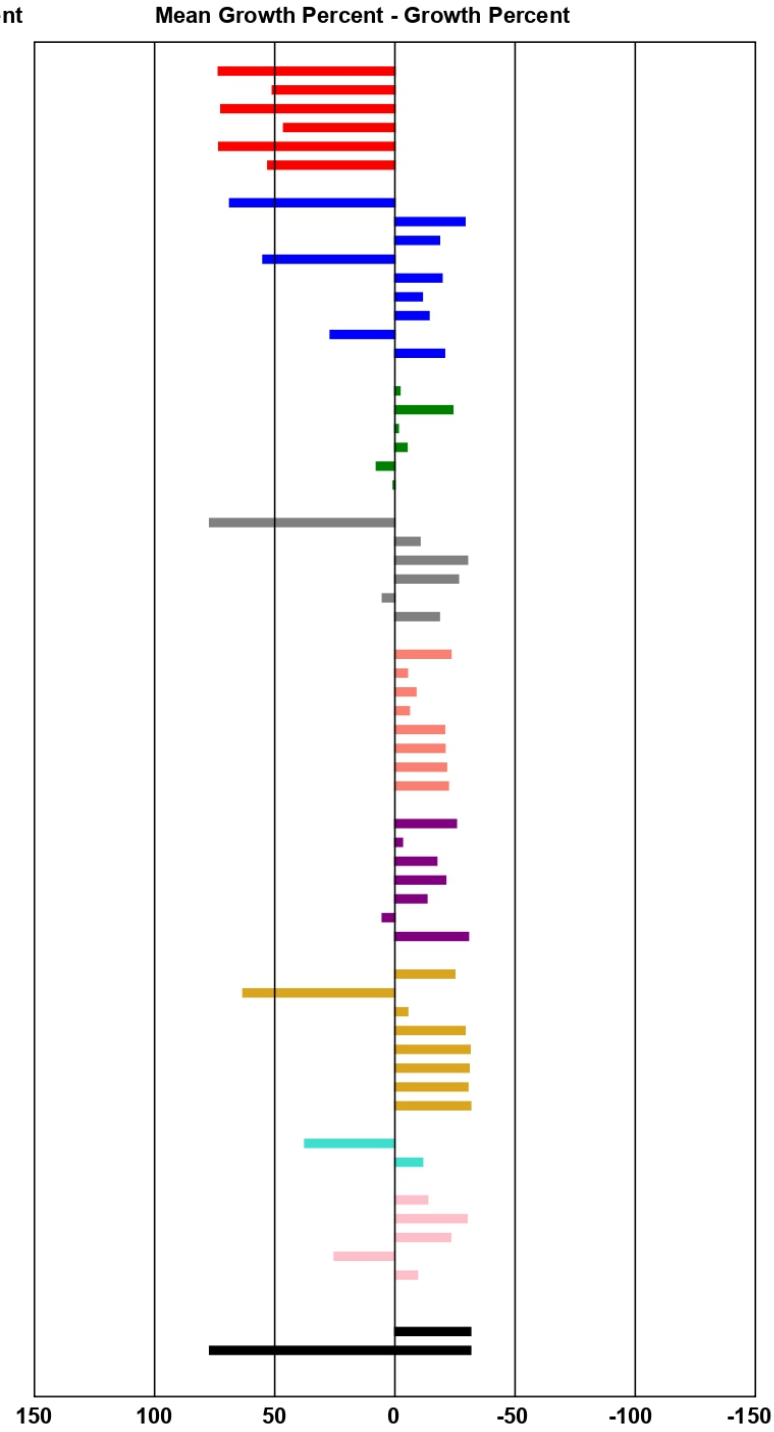

Figure S2. One-Dose Assay of compound **1** at 10 μM after 48h of treatment.

Panel/Cell Line	Growth Percent
**Leukemia**	
CCRF-CEM	-1.77
HL-60(TB)	-41.26
K-562	-29.63
MOLT-4	-15.39
RPMI-8226	-19.96
SR	-27.47
**Non-Small Cell Lung Cancer**	
A549/ATCC	-3.31
EKVX	-94.01
HOP-62	-70.88
HOP-92	-49.23
NCI-H226	-67.20
NCI-H23	-51.34
NCI-H322M	-82.56
NCI-H460	-70.24
NCI-H522	-97.02
**Colon Cancer**	
COLO 205	-69.04
HCC-2998	-90.99
HCT-116	-82.42
HCT-15	-45.60
HT29	-56.98
KM12	-89.61
SW-620	-72.42
**CNS Cancer**	
SF-268	-30.88
SF-295	-79.01
SF-539	-97.96
SNB-19	-88.51
SNB-75	-94.58
U251	-87.80
**Melanoma**	
LOX IMVI	-79.99
MALME-3M	-43.53
M14	-92.74
MDA-MB-435	-94.08
SK-MEL-2	-79.44
SK-MEL-28	-85.47
SK-MEL-5	-84.38
UACC-257	-95.08
UACC-62	-44.18
**Ovarian Cancer**	
IGROV1	-89.82
OVCAR-3	-93.71
OVCAR-4	-55.97
OVCAR-5	-66.45
OVCAR-8	-86.29
NCI/ADR-RES	-25.28
SK-OV-3	-95.55
**Renal Cancer**	
786-0	-95.88
A498	-24.82
ACHN	-80.77
CAKI-1	-98.53
RXF 393	-98.31
SN12C	-78.68
TK-10	-98.52
UO-31	-100.00
**Prostate Cancer**	
PC-3	-62.92
DU-145	-92.64
**Breast Cancer**	
MCF7	-54.95
MDA-MB-231/ATCC	-50.52
HS 578T	-15.58
BT-549	-94.75
T-47D	-45.65
MDA-MB-468	-64.38
Mean	-67.93
Delta	32.07
Range	98.23

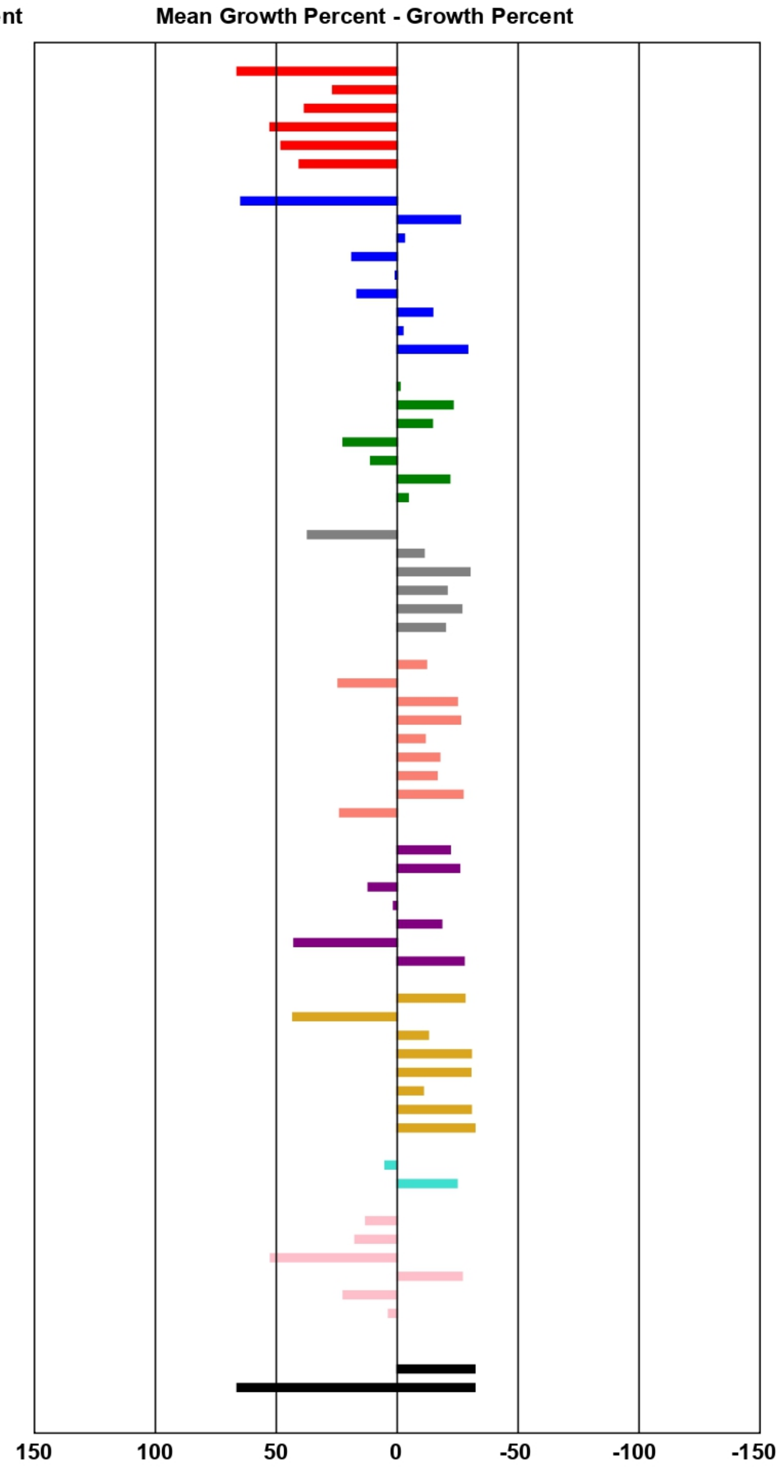

Figure S3. One-Dose Assay of compound **4** at 10 µM after 48h of treatment.

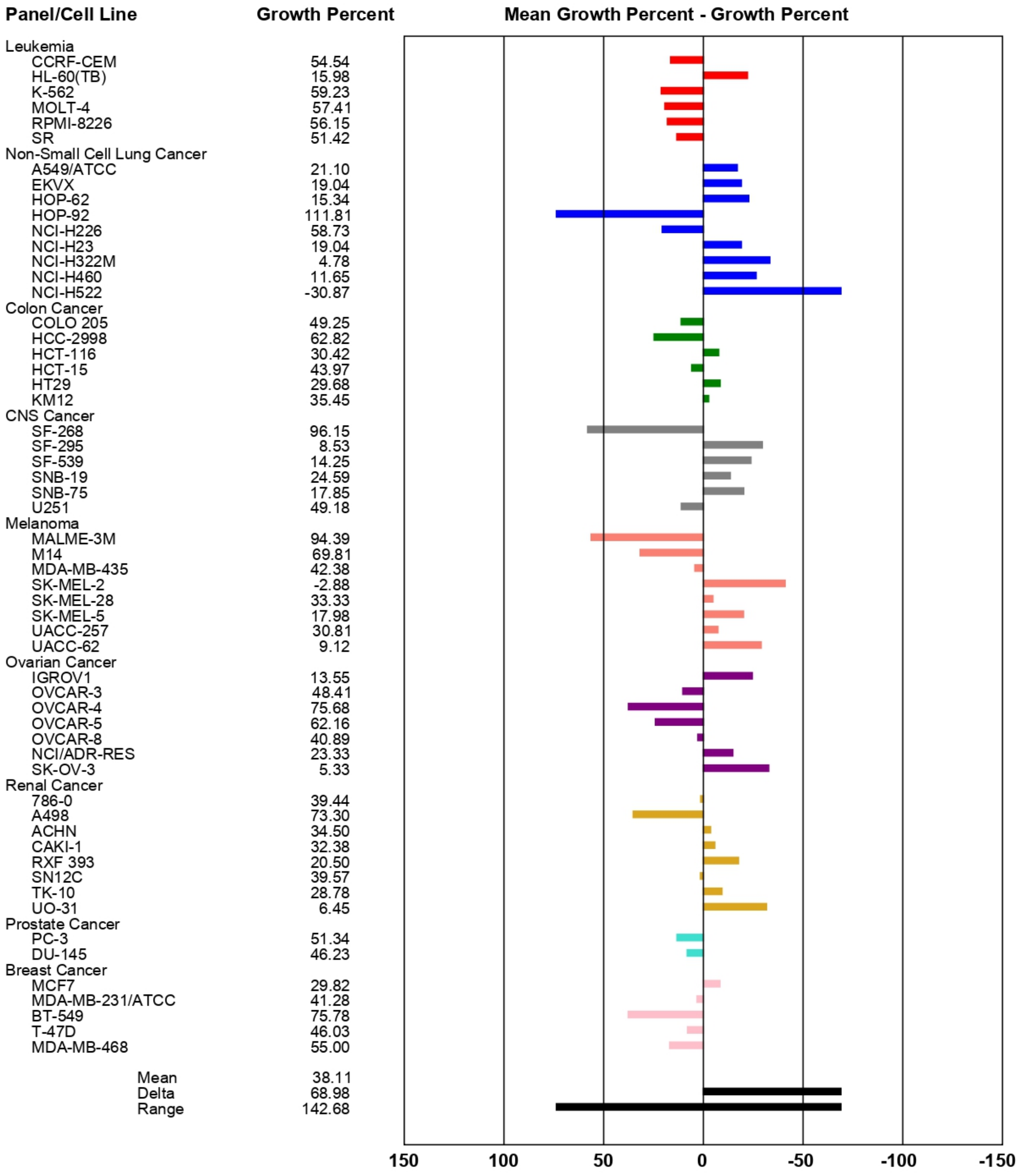

Figure S4. One-Dose Assay of compound **8** at 10 µM after 48h of treatment.

Panel/Cell Line	Growth Percent
Leukemia	
CCRF-CEM	28.55
HL-60(TB)	7.53
K-562	61.79
MOLT-4	29.39
RPMI-8226	27.94
SR	41.38
Non-Small Cell Lung Cancer	
A549/ATCC	20.44
EKVX	-58.97
HOP-62	9.51
HOP-92	32.03
NCI-H226	-21.01
NCI-H23	-0.22
NCI-H322M	0.72
NCI-H460	6.60
NCI-H522	-95.69
Colon Cancer	
COLO 205	28.87
HCC-2998	28.06
HCT-116	20.98
HCT-15	3.57
HT29	32.07
KM12	5.47
CNS Cancer	
SF-268	66.12
SF-295	-8.45
SF-539	-84.74
SNB-19	12.25
SNB-75	-75.27
U251	24.17
Melanoma	
MALME-3M	-6.63
M14	20.73
MDA-MB-435	8.85
SK-MEL-2	-43.68
SK-MEL-28	-14.68
SK-MEL-5	-79.97
UACC-257	-1.74
UACC-62	-57.53
Ovarian Cancer	
IGROV1	-46.03
OVCAR-3	-68.10
OVCAR-4	-13.25
OVCAR-5	-30.29
OVCAR-8	-56.36
NCI/ADR-RES	-16.42
SK-OV-3	-41.72
Renal Cancer	
786-0	-11.85
A498	16.34
ACHN	20.23
CAKI-1	-75.36
RXF 393	-68.27
SN12C	-42.69
TK-10	-52.89
UO-31	-87.70
Prostate Cancer	
PC-3	44.62
DU-145	19.98
Breast Cancer	
MCF7	1.18
MDA-MB-231/ATCC	-25.68
BT-549	33.81
T-47D	-9.49
MDA-MB-468	-4.29
Mean	-9.58
Delta	86.11
Range	161.81

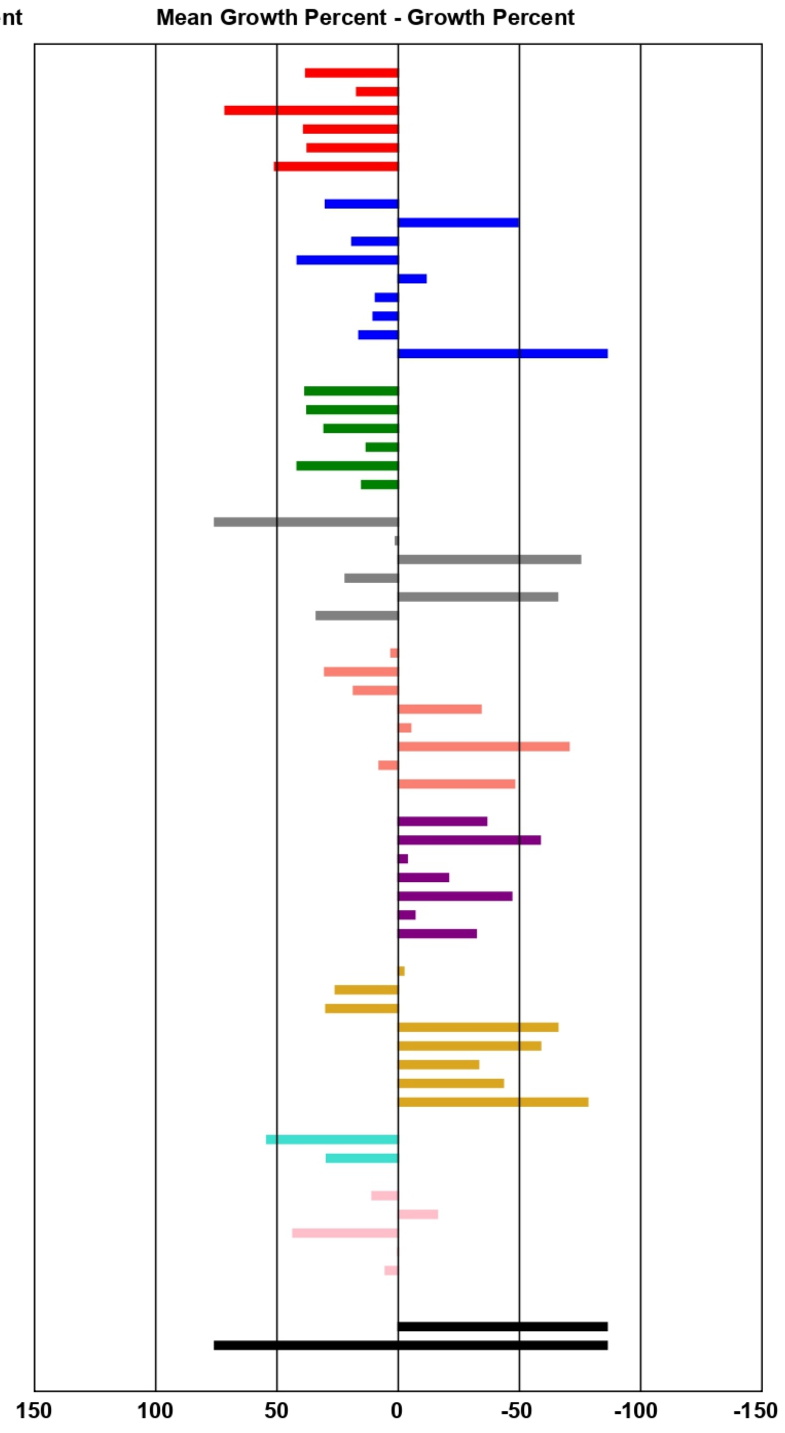

Figure S5. One-Dose Assay of compound **9** at 10 μM after 48h of treatment.

Panel/Cell Line	Growth Percent
Leukemia	
CCRF-CEM	0.40
HL-60(TB)	-11.67
K-562	60.16
MOLT-4	24.90
RPMI-8226	-4.65
SR	7.50
Non-Small Cell Lung Cancer	
A549/ATCC	23.88
EKVX	13.79
HOP-62	-1.42
HOP-92	76.44
NCI-H226	34.07
NCI-H23	-10.35
NCI-H322M	4.94
NCI-H460	5.23
NCI-H522	40.87
Colon Cancer	
COLO 205	80.05
HCC-2998	39.85
HCT-116	4.07
HCT-15	37.73
HT29	33.08
KM12	2.24
SW-620	17.83
CNS Cancer	
SF-268	80.41
SF-295	-4.41
SF-539	-8.37
SNB-19	4.56
SNB-75	-3.47
U251	7.55
Melanoma	
LOX IMVI	52.41
MALME-3M	85.19
M14	8.70
MDA-MB-435	45.38
SK-MEL-2	15.02
SK-MEL-28	41.54
SK-MEL-5	12.79
UACC-257	45.77
UACC-62	0.10
Ovarian Cancer	
IGROV1	32.49
OVCAR-3	53.66
OVCAR-4	70.46
OVCAR-5	5.61
OVCAR-8	1.84
NCI/ADR-RES	14.26
SK-OV-3	-20.00
Renal Cancer	
786-0	0.86
A498	42.38
ACHN	33.60
CAKI-1	46.08
RXF 393	38.61
TK-10	51.67
UO-31	5.16
Prostate Cancer	
PC-3	9.00
DU-145	14.40
Breast Cancer	
MCF7	25.68
MDA-MB-231/ATCC	9.77
HS 578T	-9.44
BT-549	44.90
T-47D	29.05
MDA-MB-468	40.01
Mean	23.77
Delta	43.77
Range	105.19

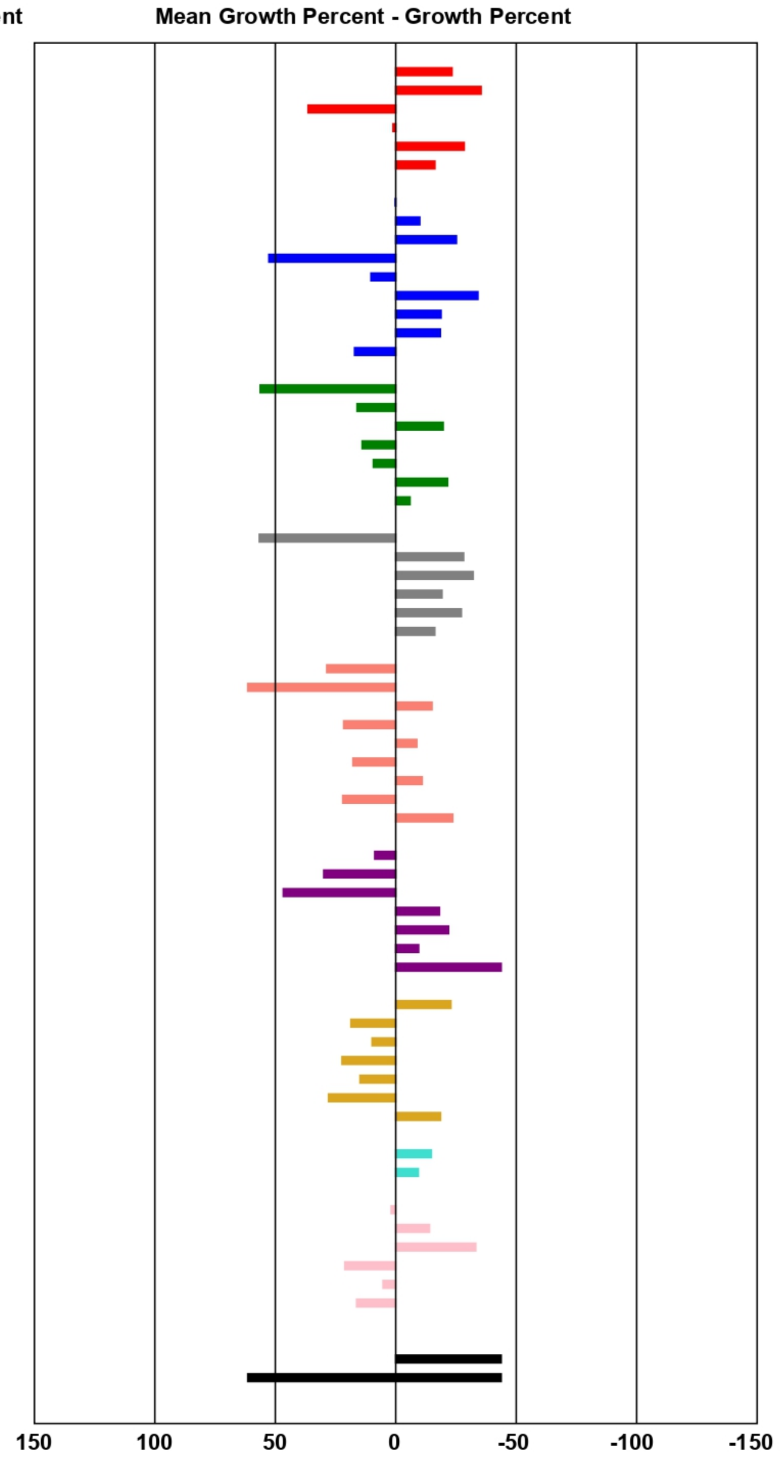

Figure S6. One-Dose Assay of compound **10** at 10 μM after 48h of treatment.

Panel/Cell Line	Growth Percent
Leukemia	
CCRF-CEM	35.25
HL-60(TB)	65.70
K-562	74.28
MOLT-4	55.56
RPMI-8226	63.05
SR	60.27
Non-Small Cell Lung Cancer	
A549/ATCC	26.82
EKVX	56.77
HOP-62	23.30
HOP-92	118.21
NCI-H226	85.58
NCI-H23	84.62
NCI-H322M	11.93
NCI-H460	10.59
NCI-H522	42.06
Colon Cancer	
COLO 205	28.08
HCC-2998	74.06
HCT-116	24.73
HCT-15	47.33
HT29	55.58
KM12	32.38
SW-620	41.20
CNS Cancer	
SF-268	122.20
SF-295	6.53
SF-539	56.89
SNB-19	33.30
SNB-75	10.59
U251	27.61
Melanoma	
LOX IMVI	81.58
MALME-3M	57.31
M14	60.21
MDA-MB-435	86.40
SK-MEL-2	99.62
SK-MEL-28	51.60
SK-MEL-5	79.28
UACC-257	50.49
UACC-62	76.30
Ovarian Cancer	
IGROV1	43.56
OVCAR-3	25.26
OVCAR-4	70.47
OVCAR-5	71.97
OVCAR-8	37.72
NCI/ADR-RES	34.40
SK-OV-3	8.53
Renal Cancer	
786-0	44.13
A498	31.32
ACHN	17.04
CAKI-1	39.56
RXF 393	65.92
SN12C	51.88
TK-10	55.48
UO-31	38.52
Prostate Cancer	
PC-3	36.44
DU-145	48.98
Breast Cancer	
MCF7	81.56
MDA-MB-231/ATCC	68.47
HS 578T	60.84
BT-549	96.71
T-47D	-0.39
MDA-MB-468	77.13
Mean	52.05
Delta	52.44
Range	122.59

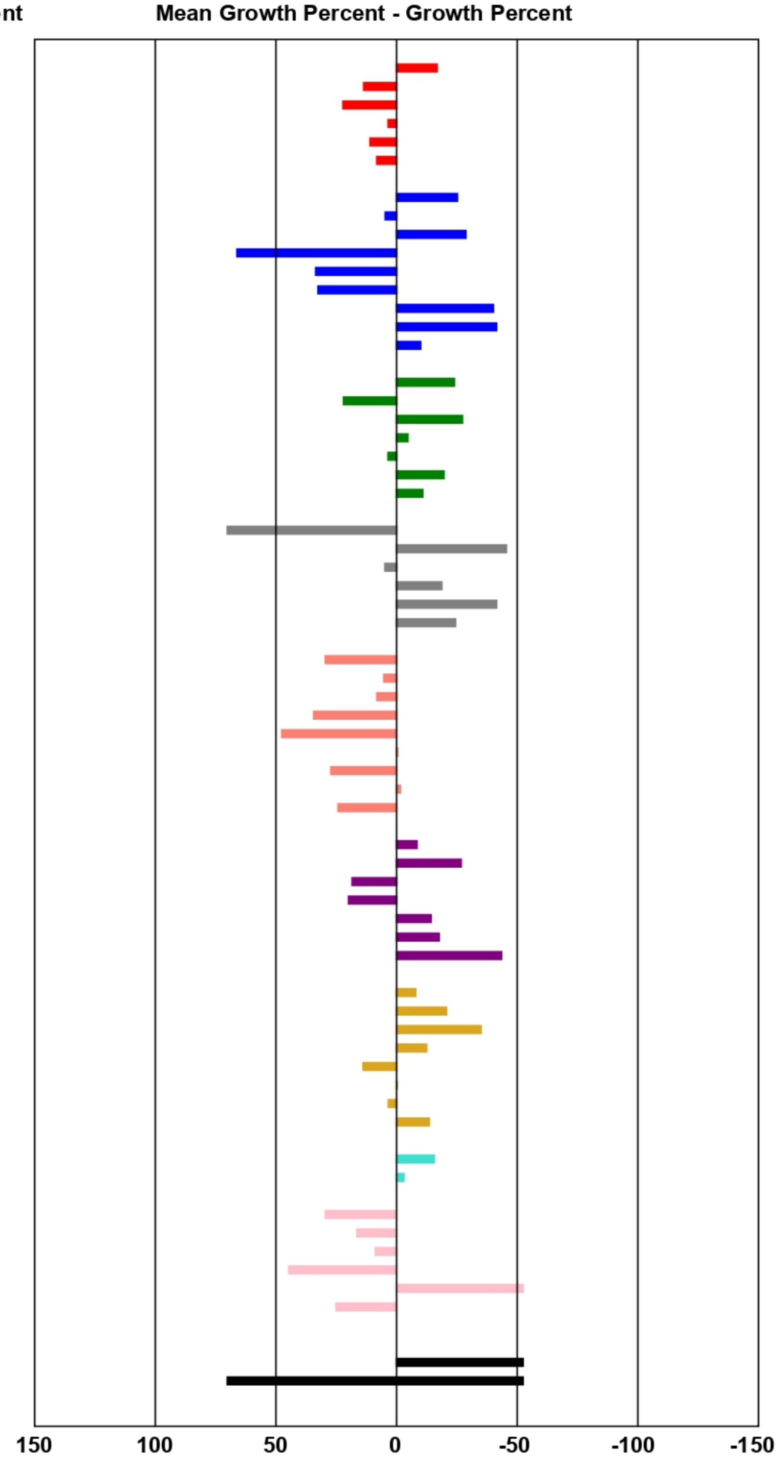

Figure S7. One-Dose Assay of compound **12** at 10 μM after 48h of treatment.

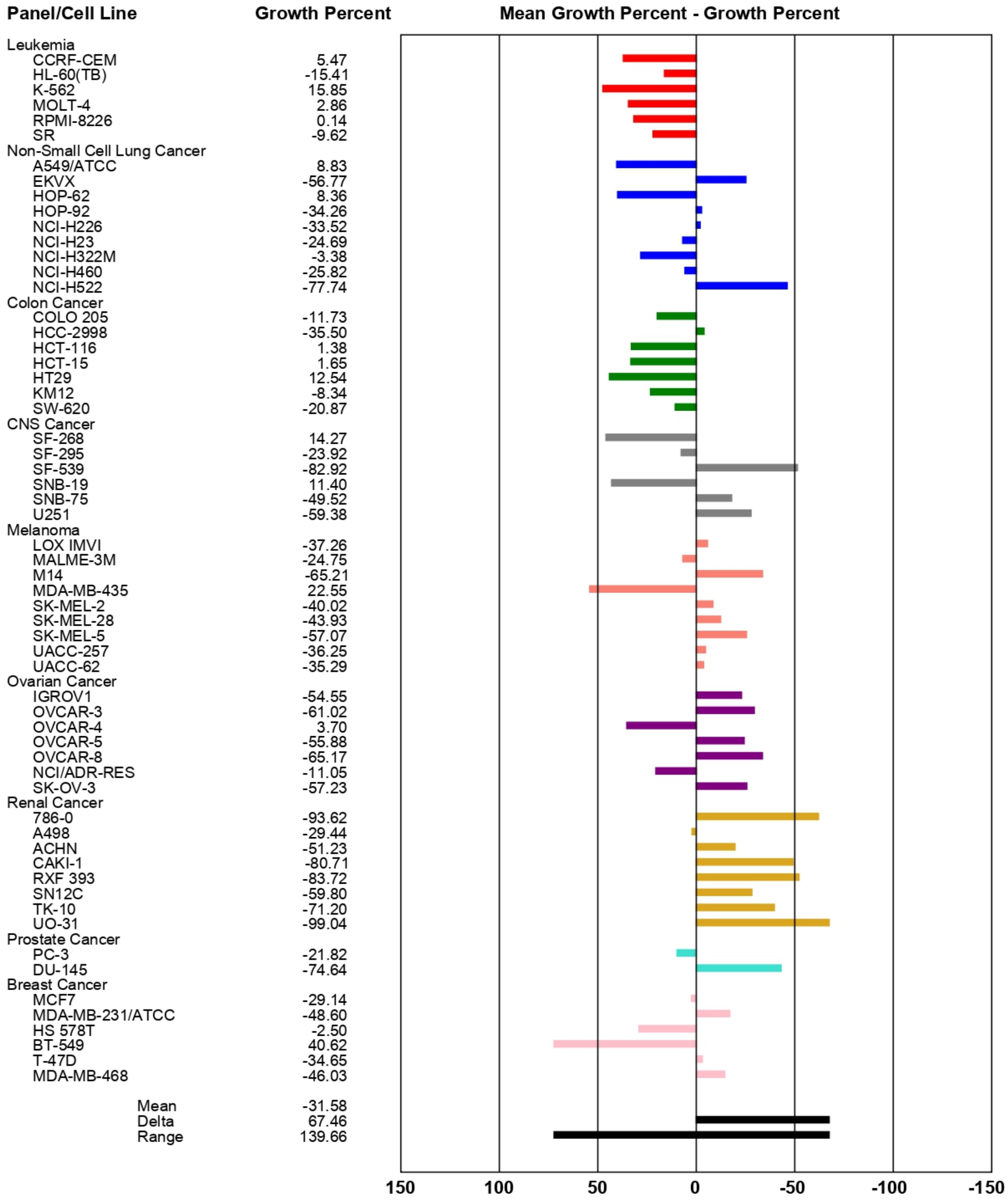

Figure S8. One-Dose Assay of compound **15** at 10 μM after 48h of treatment.

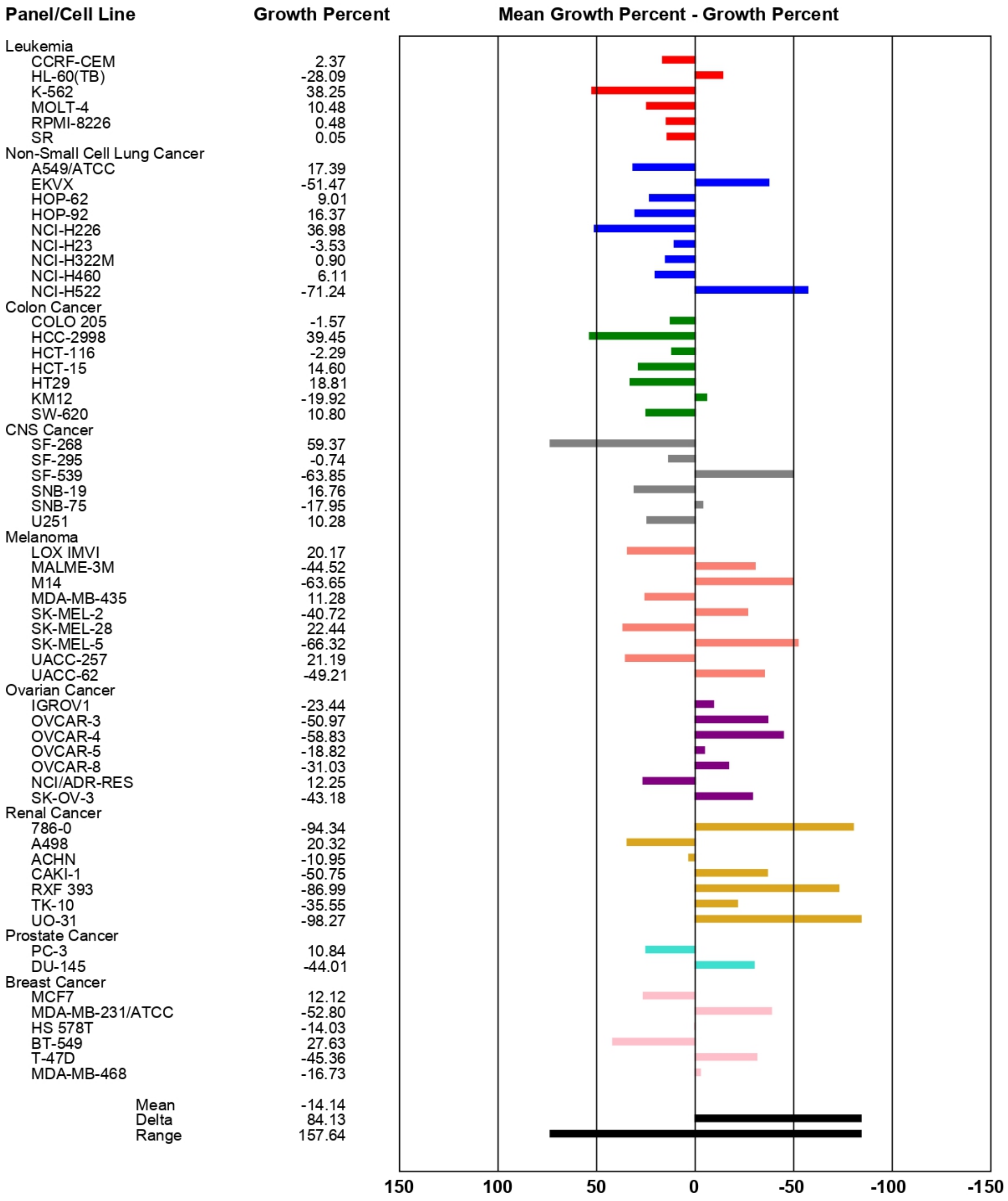

Figure S9. One-Dose Assay of compound **16** at 10 μM after 48h of treatment.

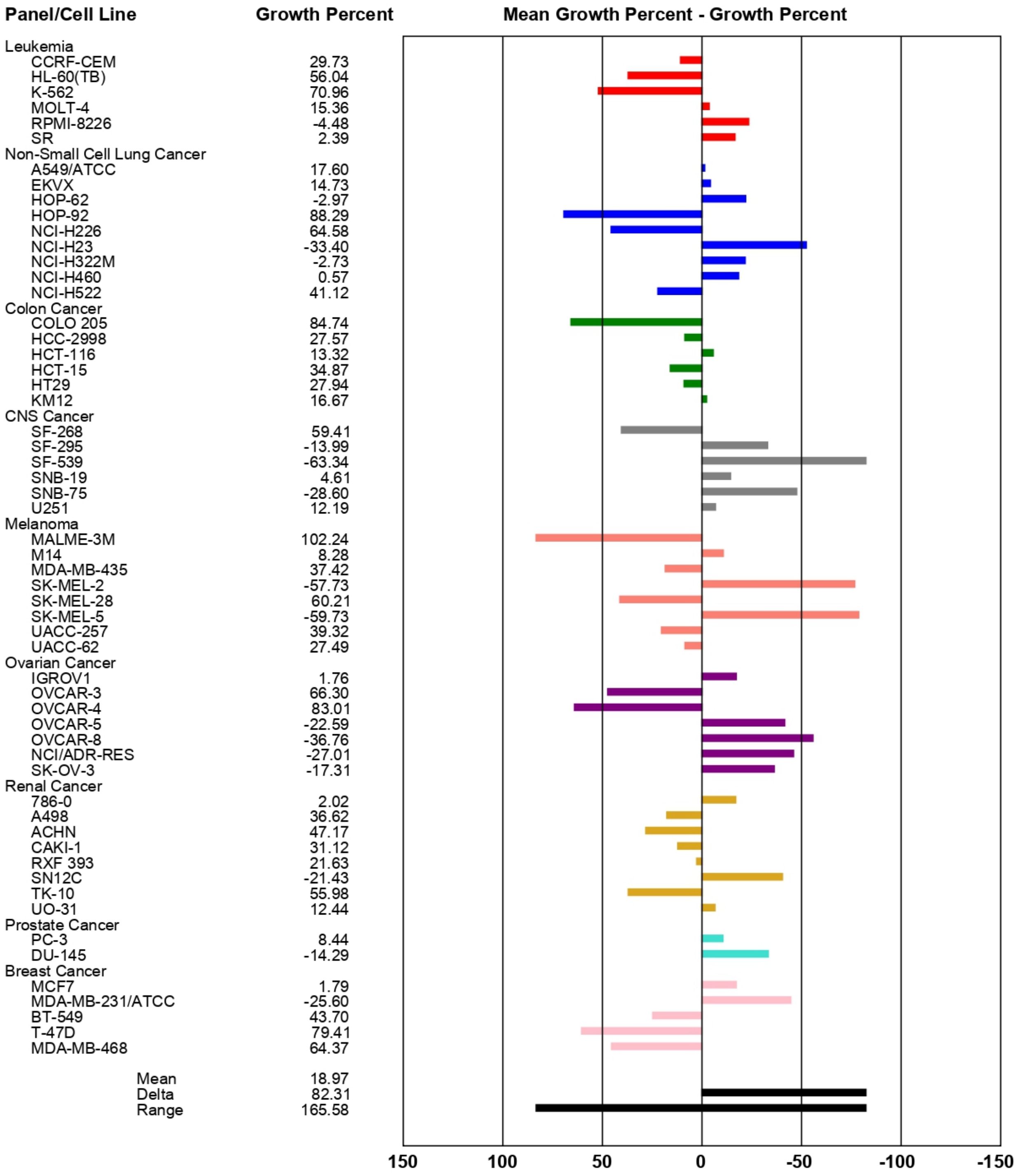

Figure S10. One-Dose Assay of compound **17** at 10 μM after 48h of treatment.

Panel/Cell Line	Growth Percent
Leukemia	
CCRF-CEM	105.23
HL-60(TB)	106.81
K-562	91.78
MOLT-4	115.55
RPMI-8226	102.91
SR	120.03
Non-Small Cell Lung Cancer	
A549/ATCC	101.16
EKVX	115.75
HOP-62	112.14
HOP-92	153.31
NCI-H226	128.10
NCI-H23	98.67
NCI-H322M	110.05
NCI-H460	107.77
NCI-H522	109.50
Colon Cancer	
COLO 205	113.25
HCC-2998	111.74
HCT-116	106.83
HCT-15	109.10
HT29	106.45
KM12	108.57
SW-620	107.71
CNS Cancer	
SF-268	114.85
SF-295	112.92
SF-539	103.15
SNB-19	111.85
SNB-75	115.33
U251	106.51
Melanoma	
LOX IMVI	103.12
MALME-3M	103.83
M14	96.61
MDA-MB-435	106.05
SK-MEL-2	139.88
SK-MEL-28	106.75
SK-MEL-5	99.92
UACC-257	102.72
UACC-62	105.23
Ovarian Cancer	
IGROV1	112.71
OVCAR-3	110.67
OVCAR-4	122.57
OVCAR-5	119.86
OVCAR-8	103.95
NCI/ADR-RES	111.41
SK-OV-3	137.47
Renal Cancer	
786-0	103.21
A498	154.05
ACHN	107.35
CAKI-1	98.62
RXF 393	143.70
SN12C	103.88
TK-10	129.50
UO-31	119.23
Prostate Cancer	
PC-3	110.58
DU-145	112.68
Breast Cancer	
MCF7	101.25
MDA-MB-231/ATCC	112.25
HS 578T	109.39
BT-549	117.23
T-47D	111.53
MDA-MB-468	109.94
Mean	112.24
Delta	20.46
Range	62.27

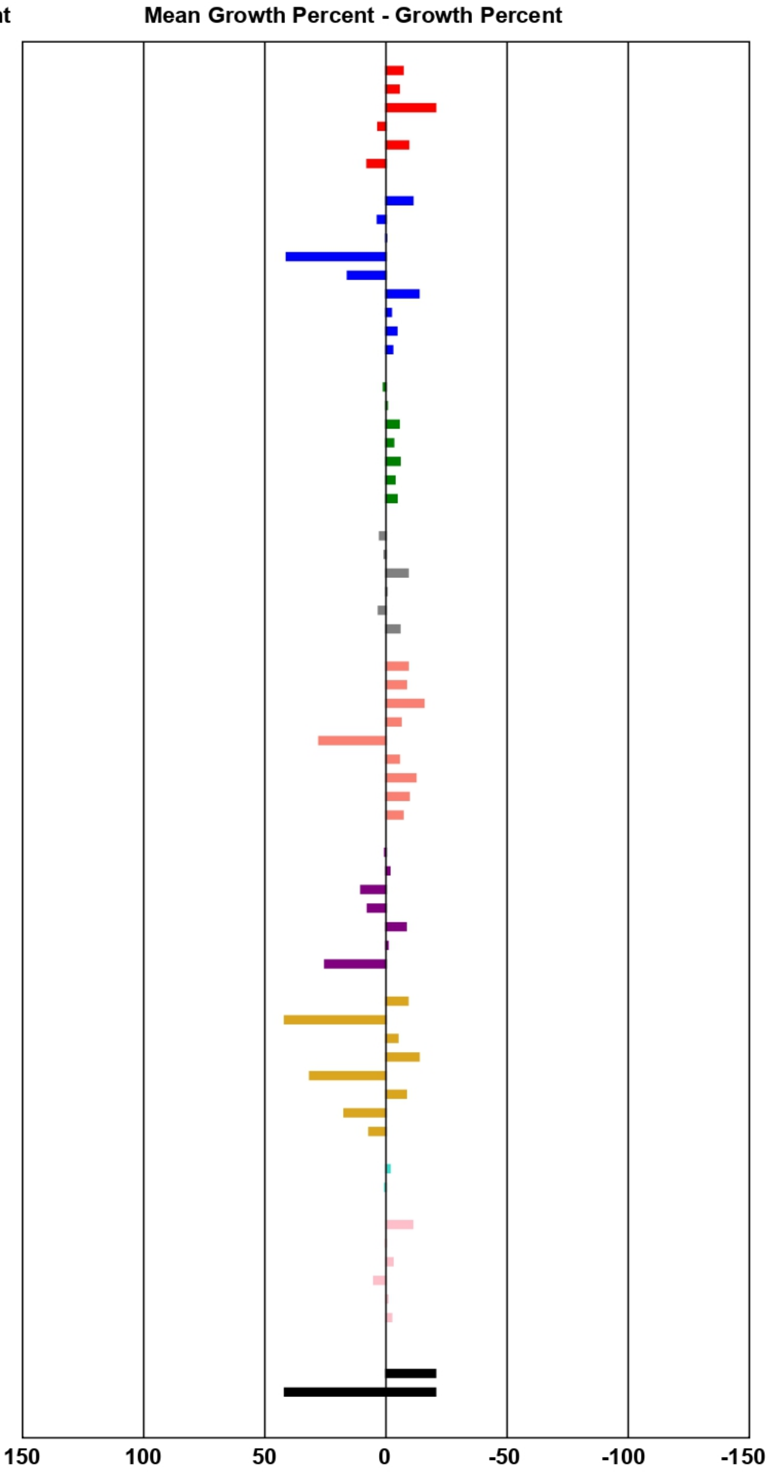

Figure S11. One-Dose Assay of compound **19** at 10 μM after 48h of treatment.

Panel/Cell Line	Log_{10}GI50	Log_{10}TGI	Log_{10}LC50
**Leukemia**			
CCRF-CEM	-5.69	-5.30	-4.15
HL-60(TB)	-5.71	-5.33	-4.45
K-562	-5.68	-5.28	-4.41
MOLT-4	-5.62	-5.25	> -4.00
RPMI-8226	-5.67	-5.27	> -4.00
SR	-5.71	-5.33	-4.41
**Non-Small Cell Lung Cancer**			
A549/ATCC	-5.52	-4.97	-4.27
EKVX	-5.78	-5.51	-5.25
HOP-62	-5.77	-5.47	-5.18
HOP-92	-5.74	-5.37	-5.00
NCI-H226	-5.80	-5.49	-5.19
NCI-H23	-5.78	-5.49	-5.21
NCI-H322M	-5.77	-5.50	-5.23
NCI-H460	-5.70	-5.36	-5.02
NCI-H522	-5.81	-5.52	-5.23
**Colon Cancer**			
COLO 205	-5.71	-5.36	-5.01
HCC-2998	-5.76	-5.51	-5.25
HCT-116	-5.72	-5.43	-5.13
HCT-15	-5.75	-5.45	-5.15
HT29	-5.66	-5.26	> -4.00
KM12	-5.74	-5.47	-5.20
SW-620	-5.75	-5.47	-5.20
**CNS Cancer**			
SF-268	-5.63	-5.25	-4.59
SF-295	-5.79	-5.50	-5.21
SF-539	-5.78	-5.52	-5.26
SNB-19	-5.78	-5.51	-5.24
SNB-75	-5.86	-5.56	-5.25
U251	-5.76	-5.47	-5.18
**Melanoma**			
LOX IMVI	-5.78	-5.51	-5.24
MALME-3M	-5.76	-5.49	-5.23
M14	-5.77	-5.50	-5.22
MDA-MB-435	-5.75	-5.48	-5.21
SK-MEL-2	-5.79	-5.50	-5.22
SK-MEL-28	-5.75	-5.49	-5.22
SK-MEL-5	-5.79	-5.51	-5.24
UACC-257	-5.78	-5.50	-5.23
UACC-62	-5.78	-5.48	-5.19
**Ovarian Cancer**			
IGROV1	-5.76	-5.50	-5.24
OVCAR-3	-5.74	-5.47	-5.20
OVCAR-4	-5.76	-5.48	-5.20
OVCAR-5	-5.77	-5.49	-5.21
OVCAR-8	-5.76	-5.50	-5.24
SK-OV-3	-5.77	-5.50	-5.22
**Renal Cancer**			
786-0	-5.75	-5.50	-5.25
A498	-5.64	-5.17	-4.59
ACHN	-5.75	-5.49	-5.22
CAKI-1	-5.77	-5.51	-5.24
RXF 393	-5.78	-5.52	-5.26
SN12C	-5.76	-5.51	-5.25
TK-10	-5.79	-5.52	-5.25
UO-31	-5.79	-5.53	-5.26
**Prostate Cancer**			
PC-3	-5.74	-5.44	-5.15
DU-145	-5.75	-5.48	-5.22
**Breast Cancer**			
MCF7	-5.74	-5.47	-5.20
MDA-MB-231/ATCC	-5.73	-5.45	-5.16
HS 578T	-5.69	-5.27	-4.44
BT-549	-5.79	-5.51	-5.24
T-47D	-5.70	-5.29	> -4.00
MDA-MB-468	-5.76	-5.48	-5.20
MID	-5.74	-5.44	-5.01
Delta	0.12	0.12	0.25
Range	0.34	0.59	1.26

Figure S12. Growth inhibition 50, total growth inhibition, and lethal concentration 50 values for compound **1** after 48h of treatment.

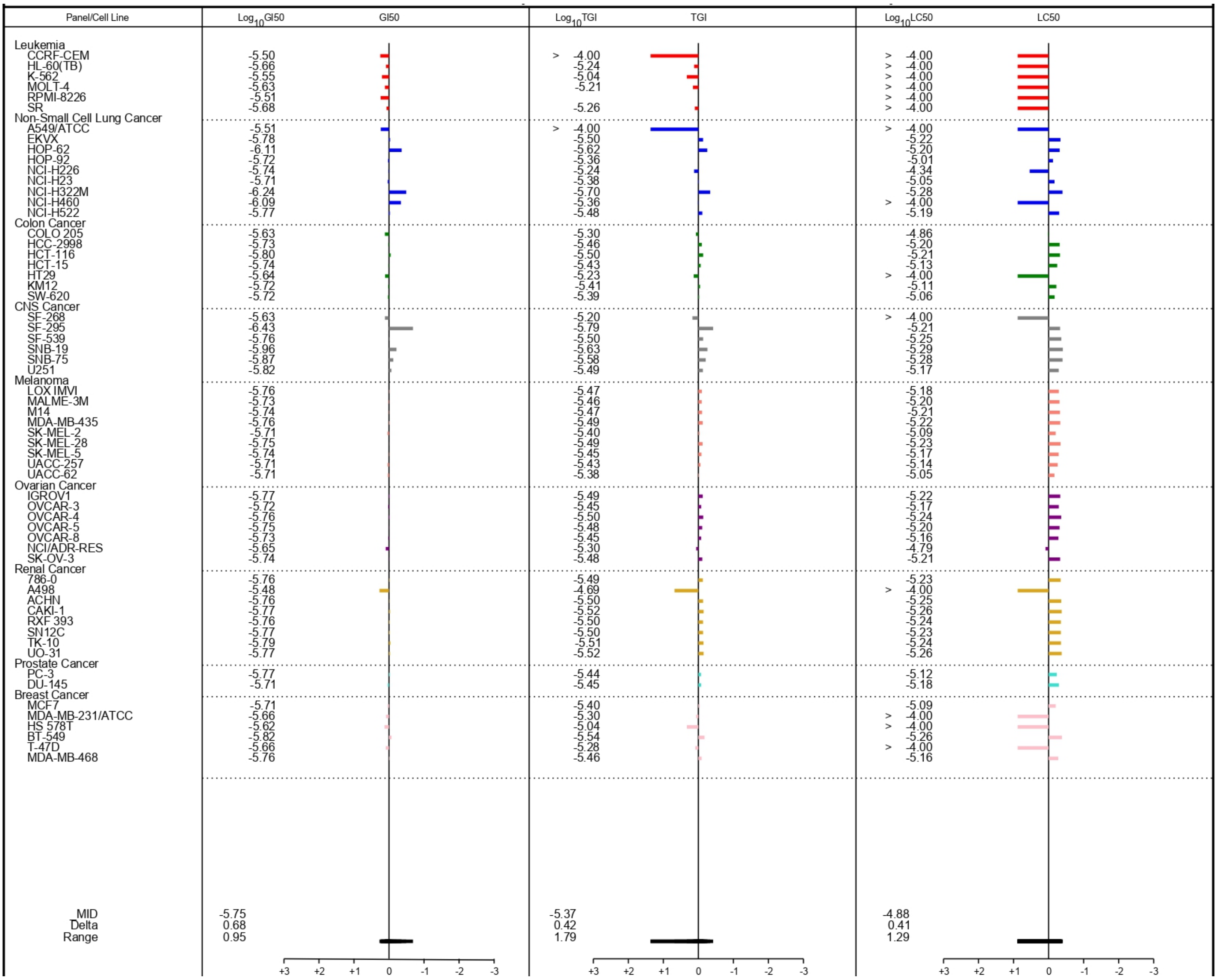

Figure S13. Growth inhibition 50, total growth inhibition, and lethal concentration 50 values for compound **4** after 48h of treatment.

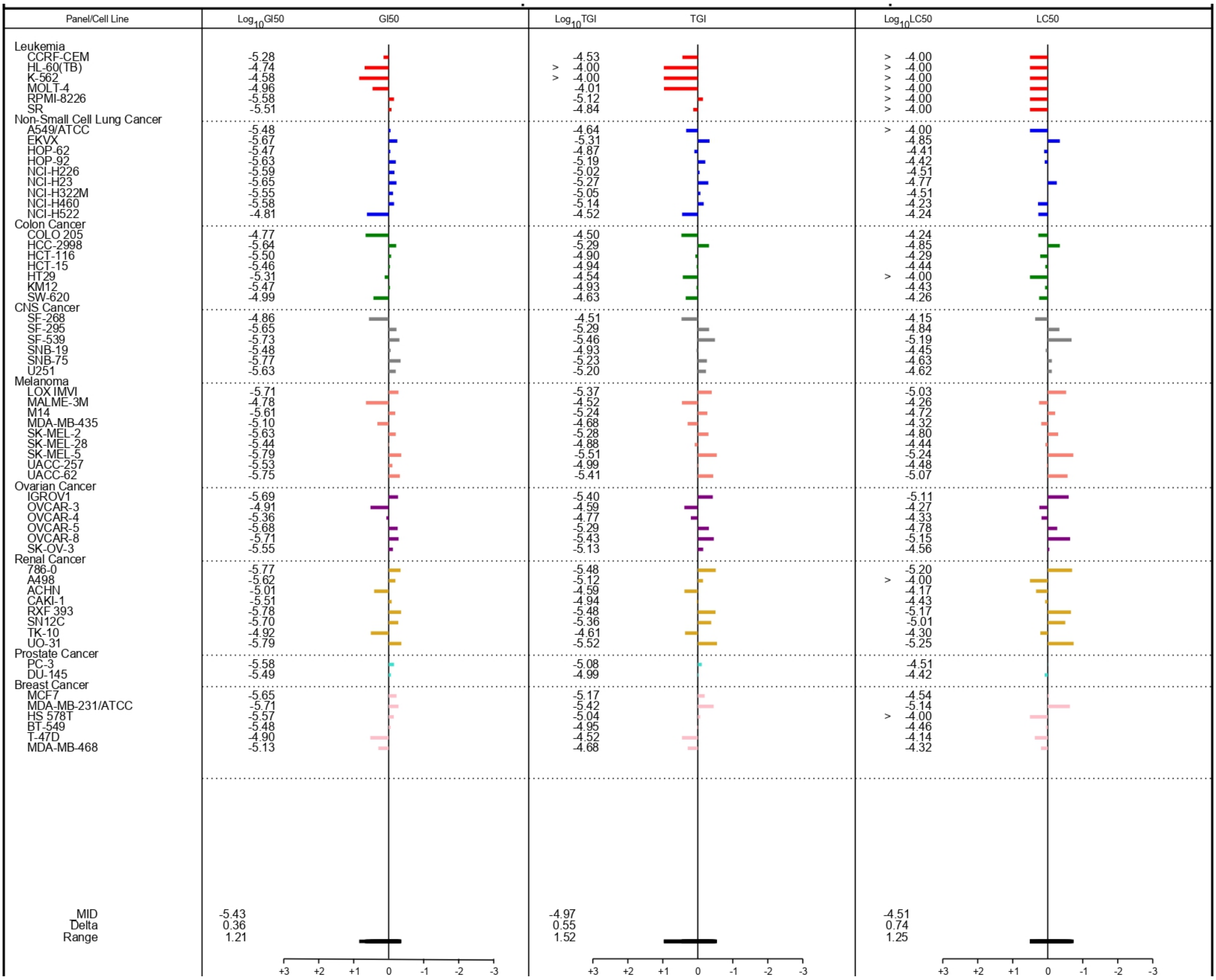

Figure S14. Growth inhibition 50, total growth inhibition, and lethal concentration 50 values for compound **9** after 48h of treatment.

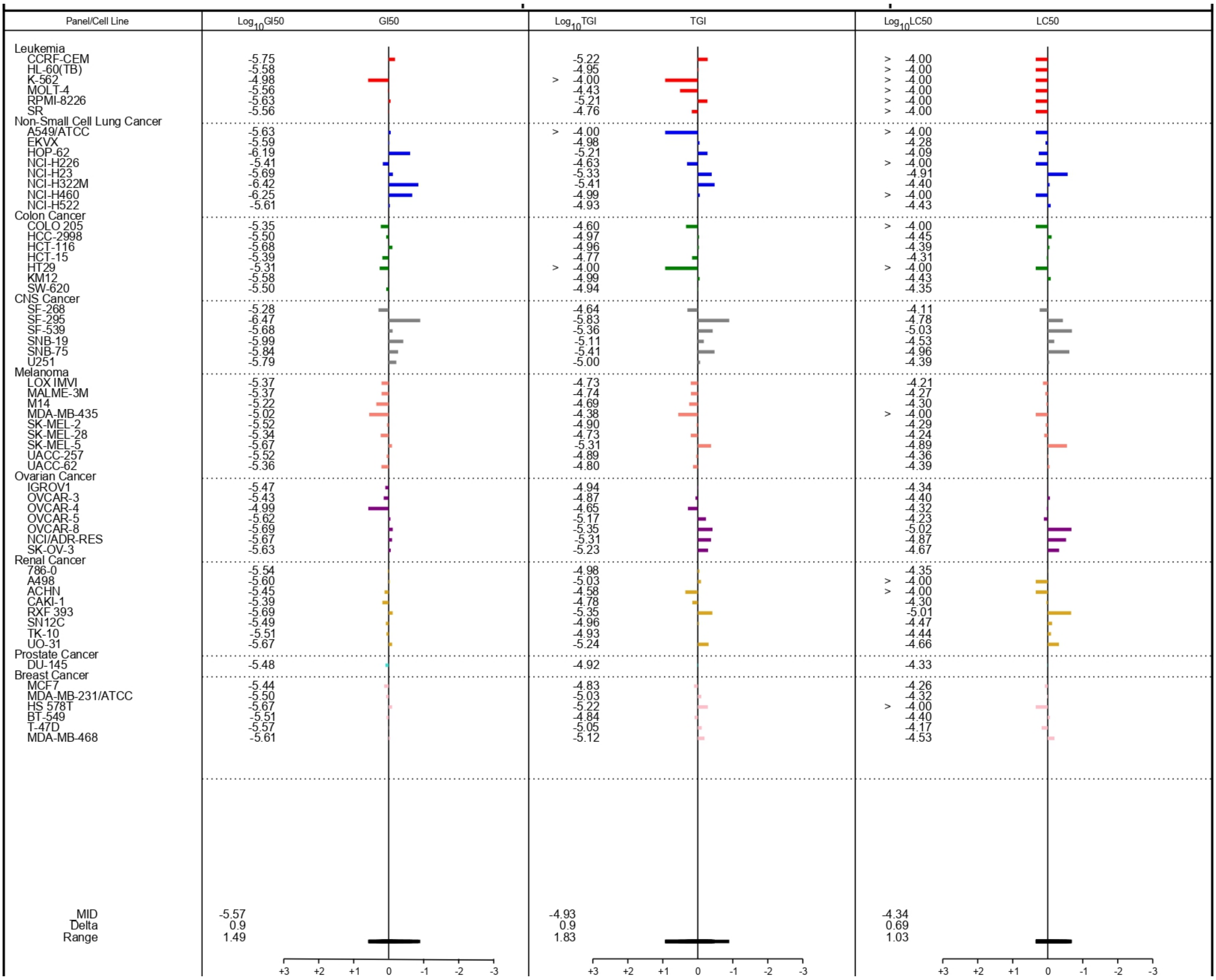

Figure S15. Growth inhibition 50, total growth inhibition, and lethal concentration 50 values for compound **10** after 48h of treatment.

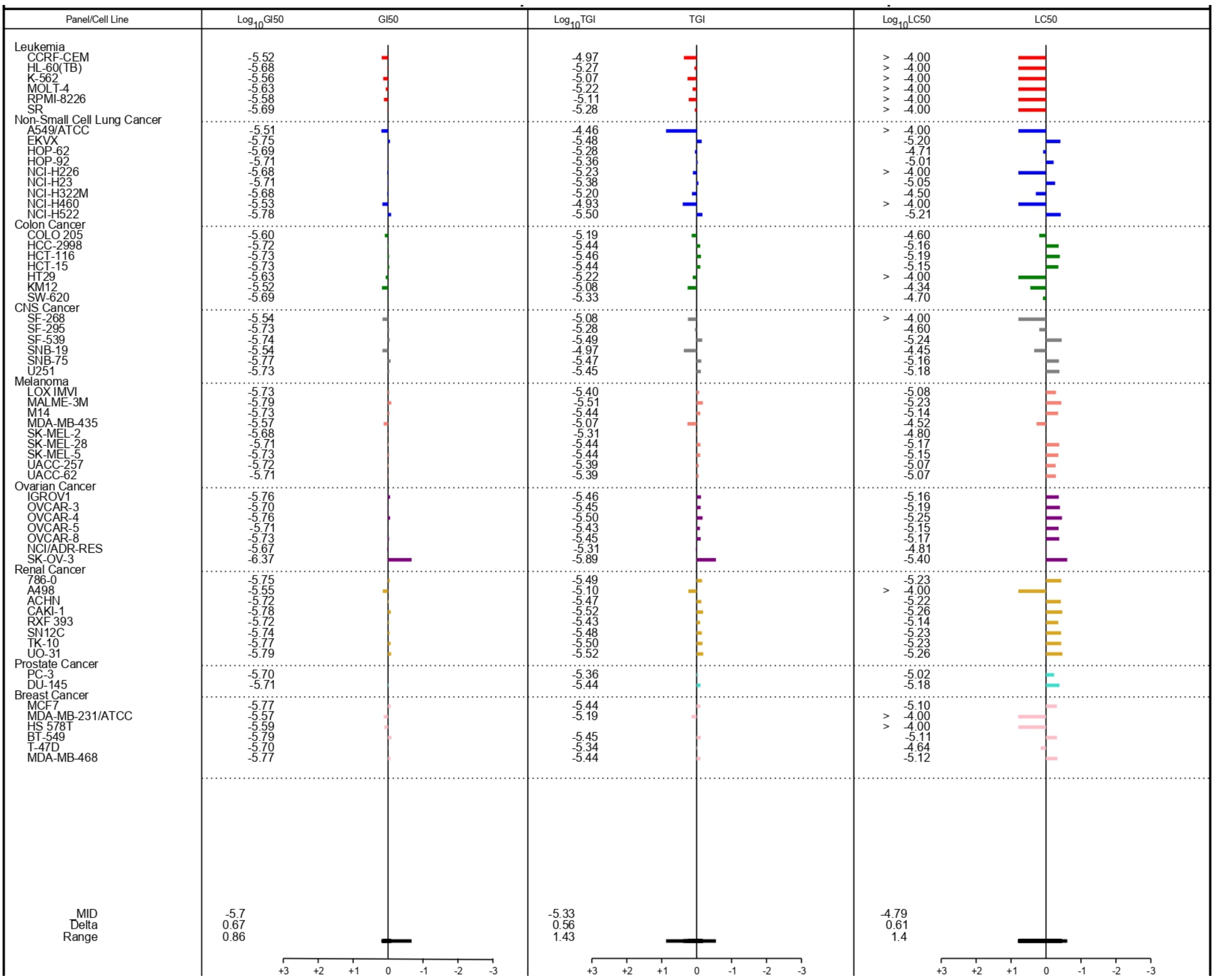

Figure S16. Growth inhibition 50, total growth inhibition, and lethal concentration 50 values for compound **15** after 48h of treatment.

Panel/Cell Line	Log_{10}GI50	Log_{10}TGI	Log_{10}LC50
**Leukemia**			
CCRF-CEM	-5.57	-5.10	> -4.00
HL-60(TB)	-5.01	> -4.00	> -4.00
K-562	-4.62	> -4.00	> -4.00
MOLT-4	-5.15	> -4.00	> -4.00
RPMI-8226	-5.55	-5.05	> -4.00
SR	-5.43	> -4.00	> -4.00
**Non-Small Cell Lung Cancer**			
A549/ATCC	-5.50	> -4.00	> -4.00
EKVX	-5.48	-4.59	> -4.00
HOP-62	-5.52	-4.80	> -4.00
NCI-H226	-5.23	-4.27	> -4.00
NCI-H23	-5.65	-5.24	> -4.00
NCI-H322M	-5.89	-5.37	> -4.00
NCI-H460	-5.69	-5.19	> -4.00
NCI-H522	-4.97	-4.61	-4.25
**Colon Cancer**			
COLO 205	-4.96	-4.54	-4.12
HCC-2998	-5.45	-4.89	-4.34
HCT-116	-5.41	> -4.00	> -4.00
HCT-15	-4.98	> -4.00	> -4.00
HT29	-5.11	> -4.00	> -4.00
KM12	-5.52	-5.04	-4.48
SW-620	-5.27	-4.73	-4.27
**CNS Cancer**			
SF-268	-5.19	> -4.00	> -4.00
SF-295	-6.10	-5.53	-5.02
SF-539	-5.34	-4.76	-4.29
SNB-19	-5.68	-5.23	> -4.00
SNB-75	-5.84	-5.44	-5.05
U251	-5.53	-4.84	> -4.00
**Melanoma**			
LOX IMVI	-4.55	> -4.00	> -4.00
MALME-3M	-4.57	> -4.00	> -4.00
M14	-4.79	-4.48	-4.17
MDA-MB-435	-4.92	> -4.00	> -4.00
SK-MEL-2	-5.47	-4.84	> -4.00
SK-MEL-28	-4.56	> -4.00	> -4.00
SK-MEL-5	-5.68	-5.34	-5.01
UACC-257	-5.24	-4.53	> -4.00
UACC-62	-5.19	-4.27	> -4.00
**Ovarian Cancer**			
IGROV1	-5.40	-4.80	-4.35
OVCAR-3	-4.96	-4.62	-4.28
OVCAR-4	-4.87	-4.57	-4.26
OVCAR-5	-5.07	-4.57	-4.10
OVCAR-8	-5.68	-5.34	-4.97
NCI/ADR-RES	-5.49	-5.02	-4.45
SK-OV-3	-5.66	-5.38	-5.09
**Renal Cancer**			
786-0	-5.37	-4.80	-4.30
A498	-5.55	-5.05	> -4.00
ACHN	-5.25	-4.00	> -4.00
CAKI-1	-4.91	> -4.00	> -4.00
RXF 393	-5.04	-4.61	-4.21
SN12C	-5.52	-4.78	> -4.00
TK-10	-4.75	-4.08	> -4.00
UO-31	-5.44	-4.82	-4.38
**Prostate Cancer**			
DU-145	-5.23	> -4.00	> -4.00
**Breast Cancer**			
MCF7	-5.15	-4.24	> -4.00
MDA-MB-231/ATCC	-5.22	-4.60	-4.03
HS 578T	-5.69	-5.17	> -4.00
BT-549	-4.90	-4.58	-4.26
T-47D	-4.92	-4.38	> -4.00
MDA-MB-468	-5.40	-4.77	-4.12
MID	-5.28	-4.6	-4.17
Delta	0.82	0.93	0.92
Range	1.55	1.53	1.09

Figure S17. Growth inhibition 50, total growth inhibition, and lethal concentration 50 values for compound **16** after 48h of treatment.

Panel/Cell Line	Log_{10}GI50	Log_{10}TGI	Log_{10}LC50
**Leukemia**			
CCRF-CEM	-4.76	-4.30	> -4.00
HL-60(TB)	-4.69	-4.26	> -4.00
K-562	-4.55	-4.06	> -4.00
MOLT-4	-4.68	-4.24	> -4.00
RPMI-8226	-4.70	-4.25	> -4.00
SR	-4.90	-4.30	> -4.00
**Non-Small Cell Lung Cancer**			
A549/ATCC	-5.37	-4.48	> -4.00
EKVX	-5.13	-4.68	-4.32
HOP-62	-5.39	-4.72	-4.05
HOP-92	-4.66	-4.29	> -4.00
NCI-H226	-4.79	-4.45	-4.12
NCI-H23	-4.99	-4.62	-4.26
NCI-H322M	-5.53	-4.94	-4.20
NCI-H460	-5.47	-4.72	> -4.00
NCI-H522	-4.84	-4.55	-4.26
**Colon Cancer**			
COLO 205	-4.78	-4.50	-4.22
HCC-2998	-5.24	-4.73	-4.36
HCT-116	-5.32	-4.73	-4.27
HCT-15	-4.74	-4.43	-4.12
HT29	-4.74	-4.33	> -4.00
KM12	-4.92	-4.58	-4.25
SW-620	-4.78	-4.48	-4.19
**CNS Cancer**			
SF-268	-4.70	-4.24	> -4.00
SF-295	-5.66	-5.17	-4.43
SF-539	-4.88	-4.58	-4.29
SNB-19	-5.32	-4.70	-4.19
SNB-75	-5.26	-4.67	-4.30
U251	-5.11	-4.67	-4.30
**Melanoma**			
LOX IMVI	-4.88	-4.57	-4.25
MALME-3M	-4.77	-4.48	-4.19
M14	-4.90	-4.58	-4.26
MDA-MB-435	-4.79	-4.48	-4.18
SK-MEL-2	-4.81	-4.46	-4.12
SK-MEL-28	-4.77	-4.45	-4.14
SK-MEL-5	-5.55	-4.85	-4.41
UACC-257	-4.81	-4.53	-4.24
UACC-62	-4.85	-4.54	-4.23
**Ovarian Cancer**			
IGROV1	-5.09	-4.67	-4.31
OVCAR-3	-4.83	-4.54	-4.26
OVCAR-4	-4.77	-4.44	-4.11
OVCAR-5	-4.80	-4.50	-4.21
OVCAR-8	-4.81	-4.53	-4.25
SK-OV-3	-4.79	-4.52	-4.24
**Renal Cancer**			
786-0	-4.75	-4.50	-4.24
A498	-5.18	-4.23	> -4.00
ACHN	-4.75	-4.43	-4.11
CAKI-1	-4.77	-4.49	-4.21
RXF 393	-4.91	-4.60	-4.30
SN12C	-4.96	-4.62	-4.28
TK-10	-4.80	-4.53	-4.26
UO-31	-4.86	-4.57	-4.29
**Prostate Cancer**			
PC-3	-5.39	-4.78	-4.31
DU-145	-4.72	-4.40	-4.08
**Breast Cancer**			
MCF7	-5.18	-4.45	> -4.00
MDA-MB-231/ATCC	-4.76	-4.44	-4.13
HS 578T	-5.15	-4.49	> -4.00
BT-549	-4.81	-4.51	-4.20
T-47D	-4.71	-4.31	> -4.00
MDA-MB-468	-4.78	-4.46	-4.14
MID	-4.94	-4.52	-4.17
Delta	0.72	0.65	0.26
Range	1.11	1.11	0.43

Figure S18. Growth inhibition 50, total growth inhibition, and lethal concentration 50 values for compound **17** after 48h of treatment.

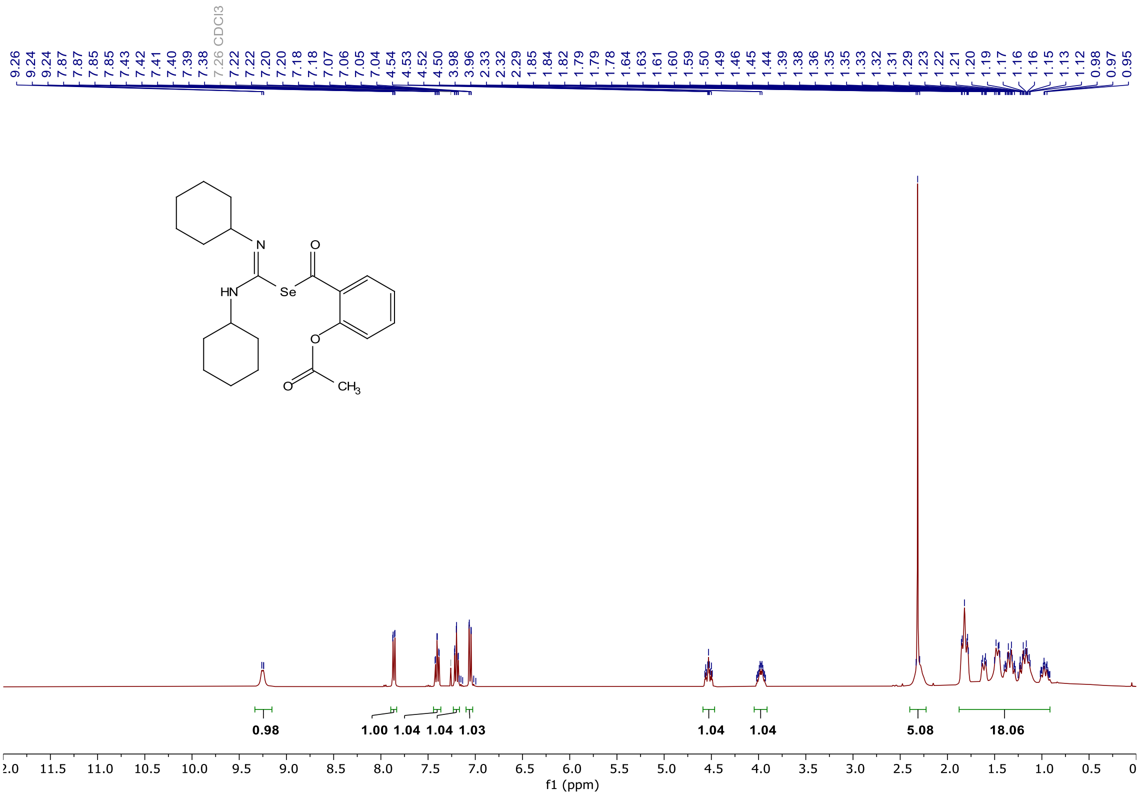

Figure S19. ^{1}H-NMR spectrum of compound **1**.

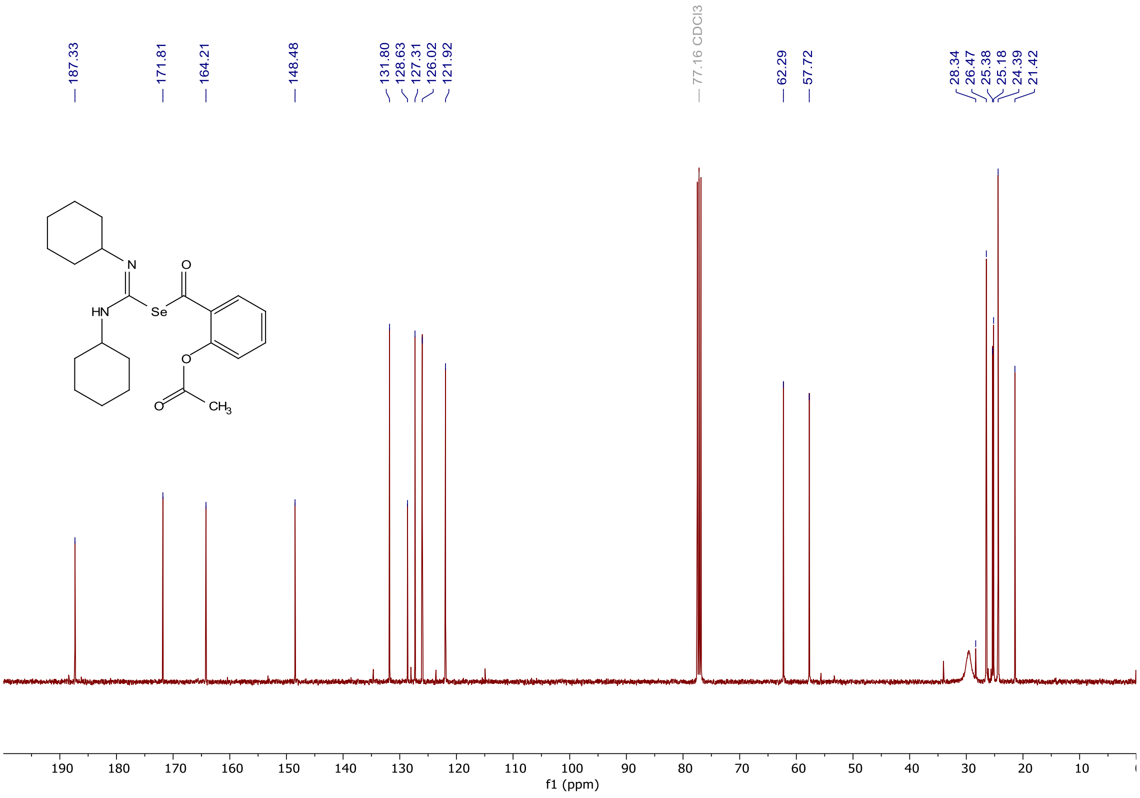

Figure S20. ^{13}C-NMR spectrum of compound **1**.

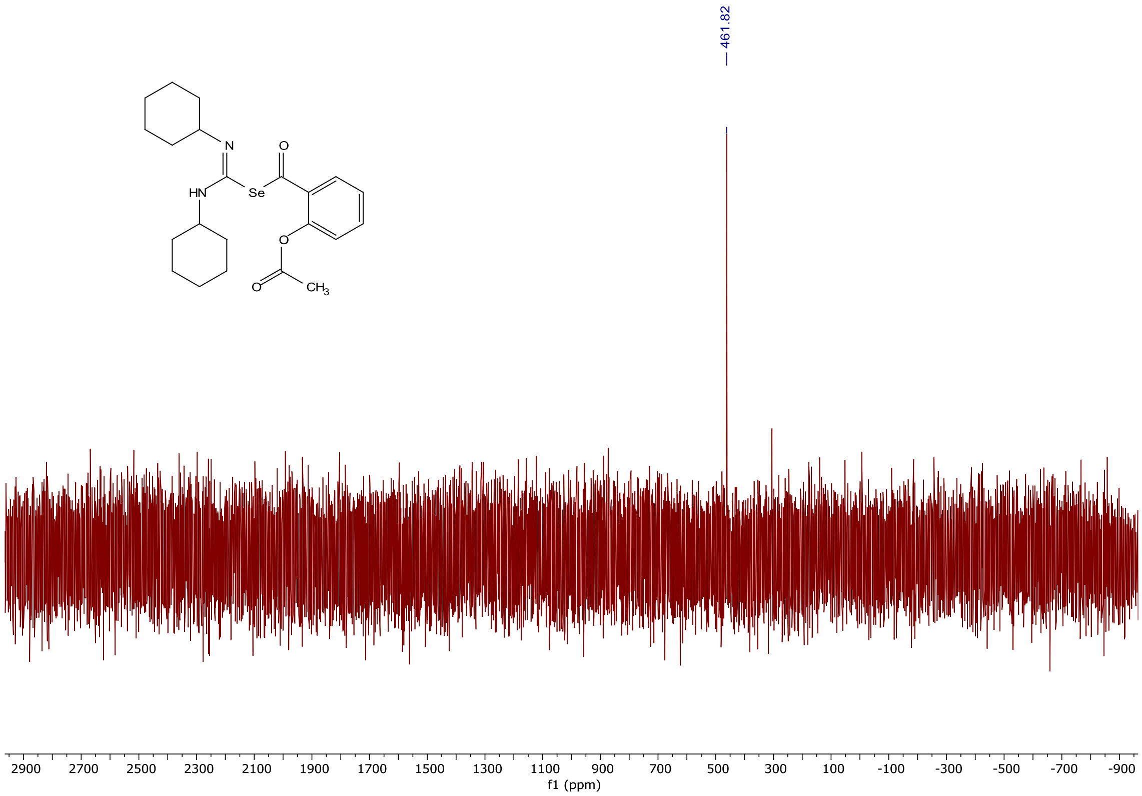

Figure S21. ^{77}Se-NMR spectrum of compound **1**.

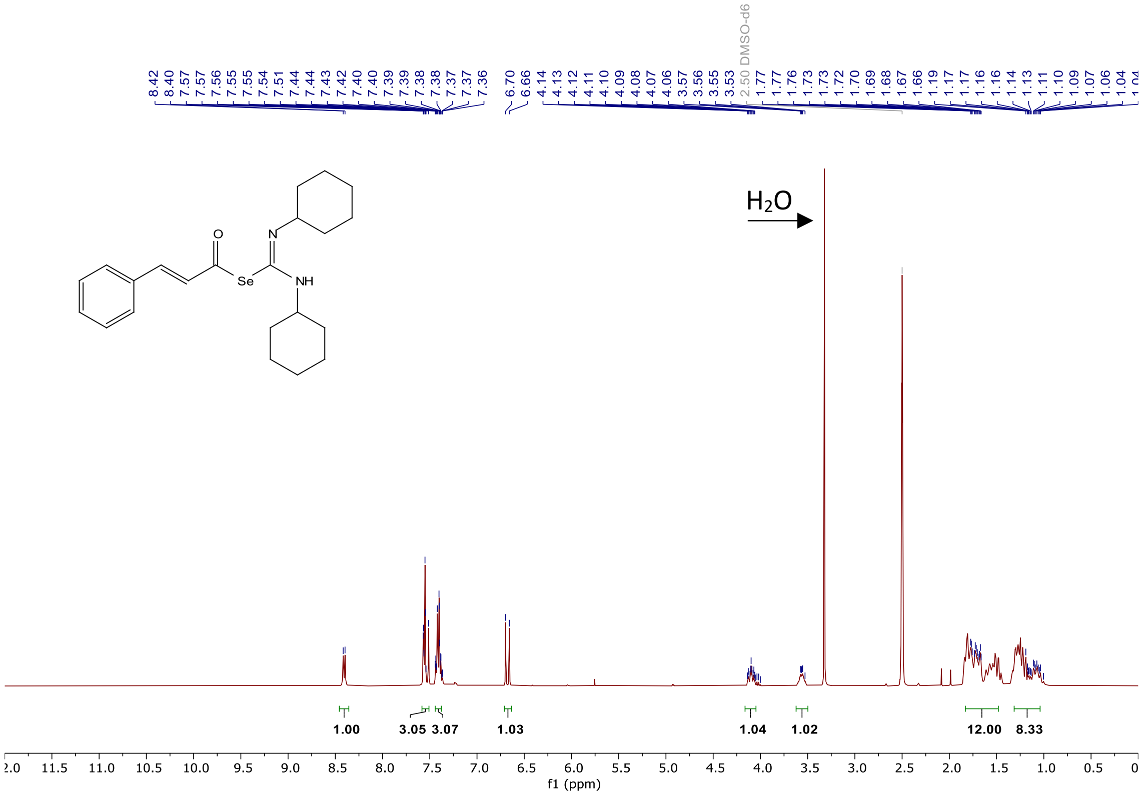

Figure S22. ^{1}H-NMR spectrum of compound **2**.

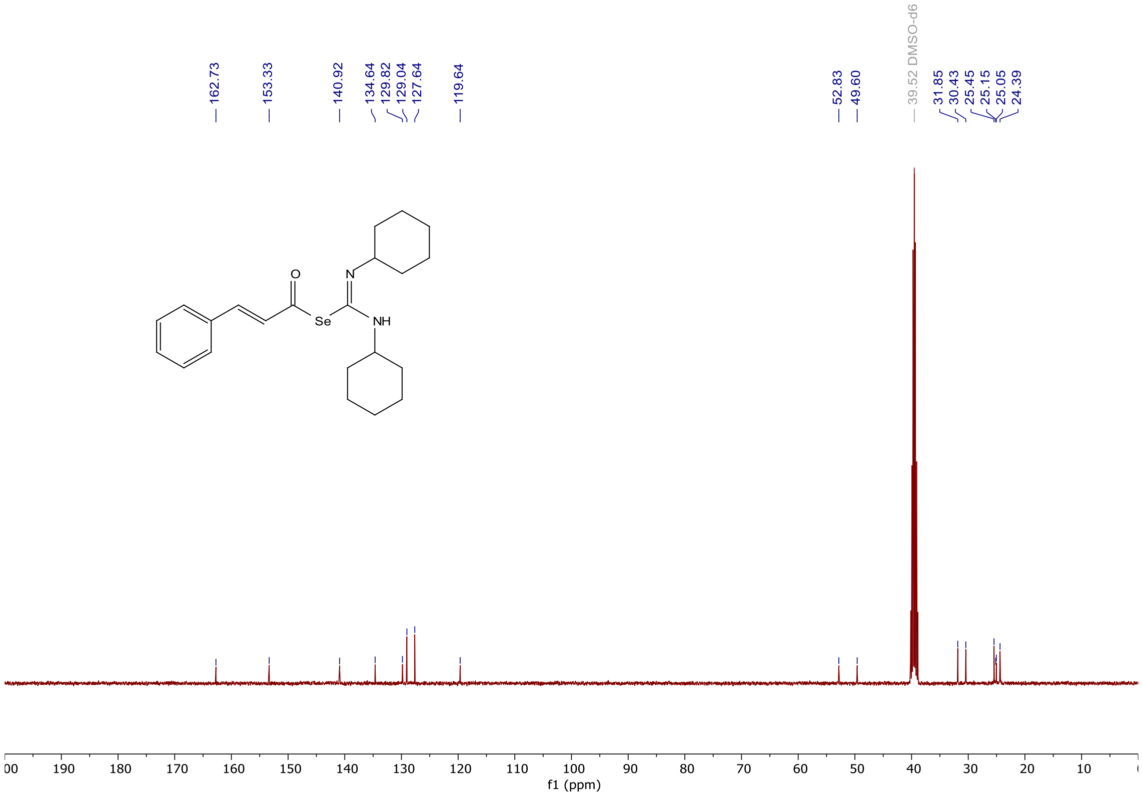

Figure S23. ^{13}C-NMR spectrum of compound **2**.

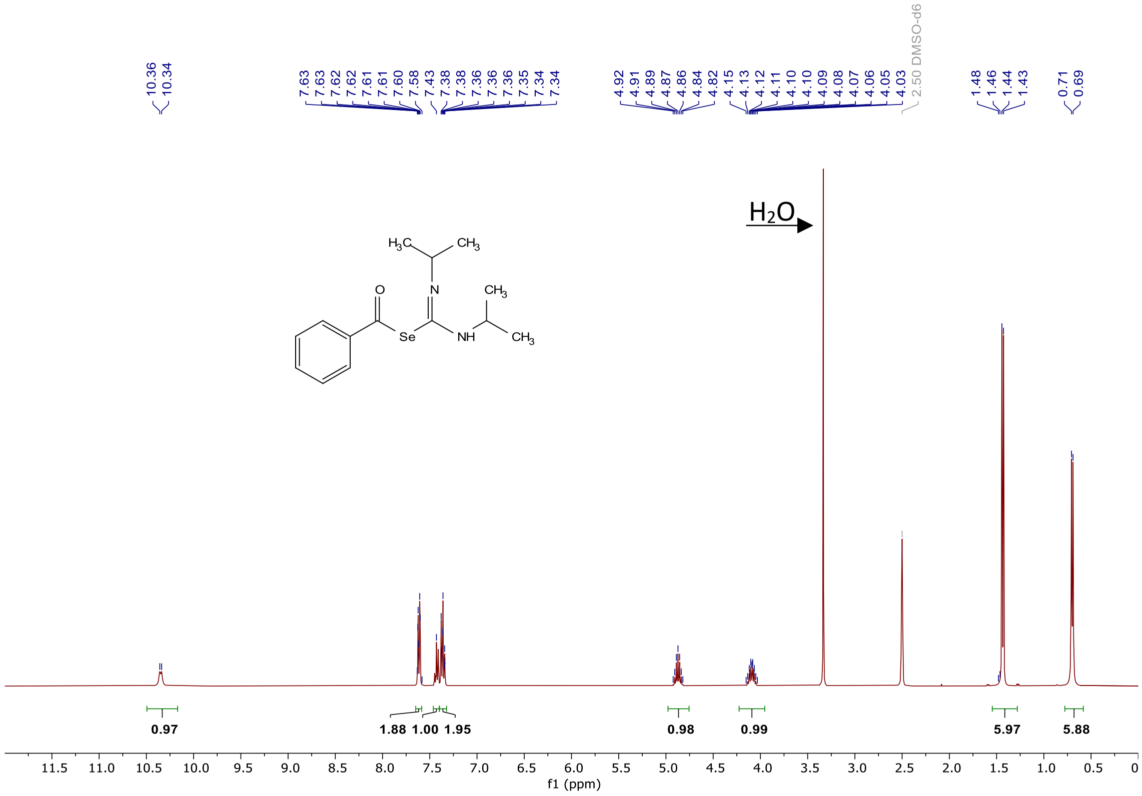

Figure S24. ^{1}H-NMR spectrum of compound **3**.

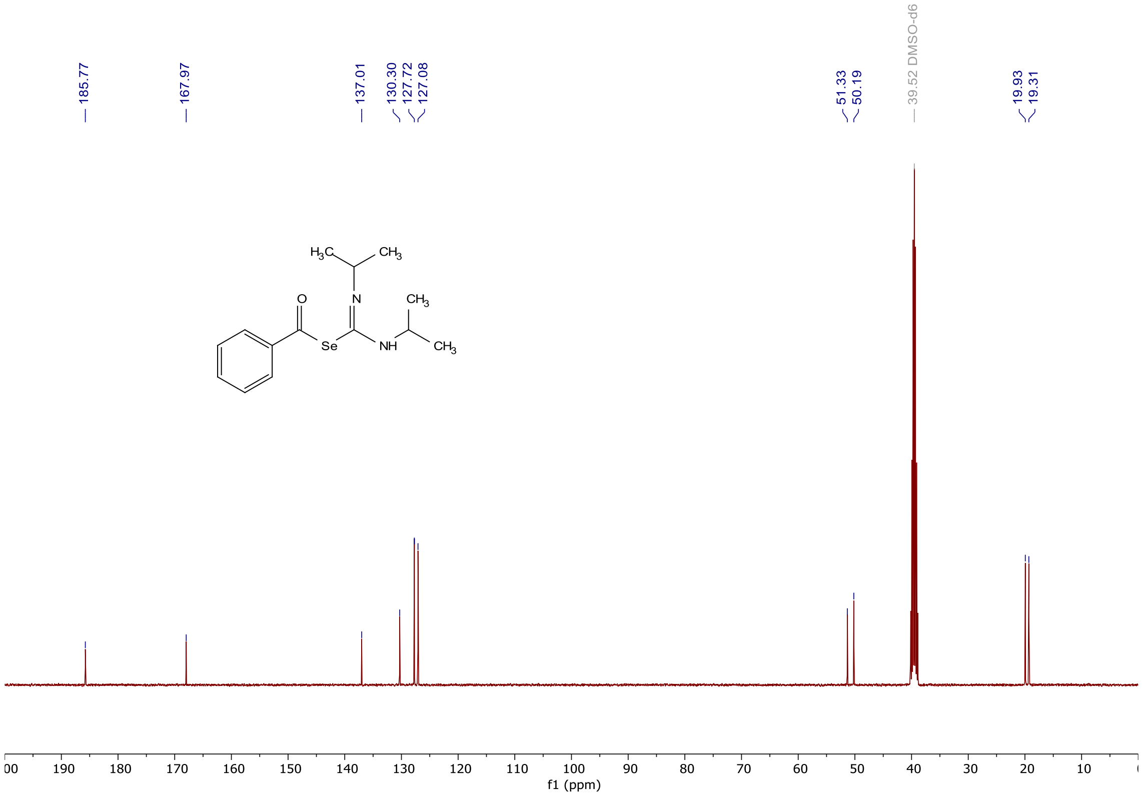

Figure S25. ^{13}C-NMR spectrum of compound **3**.

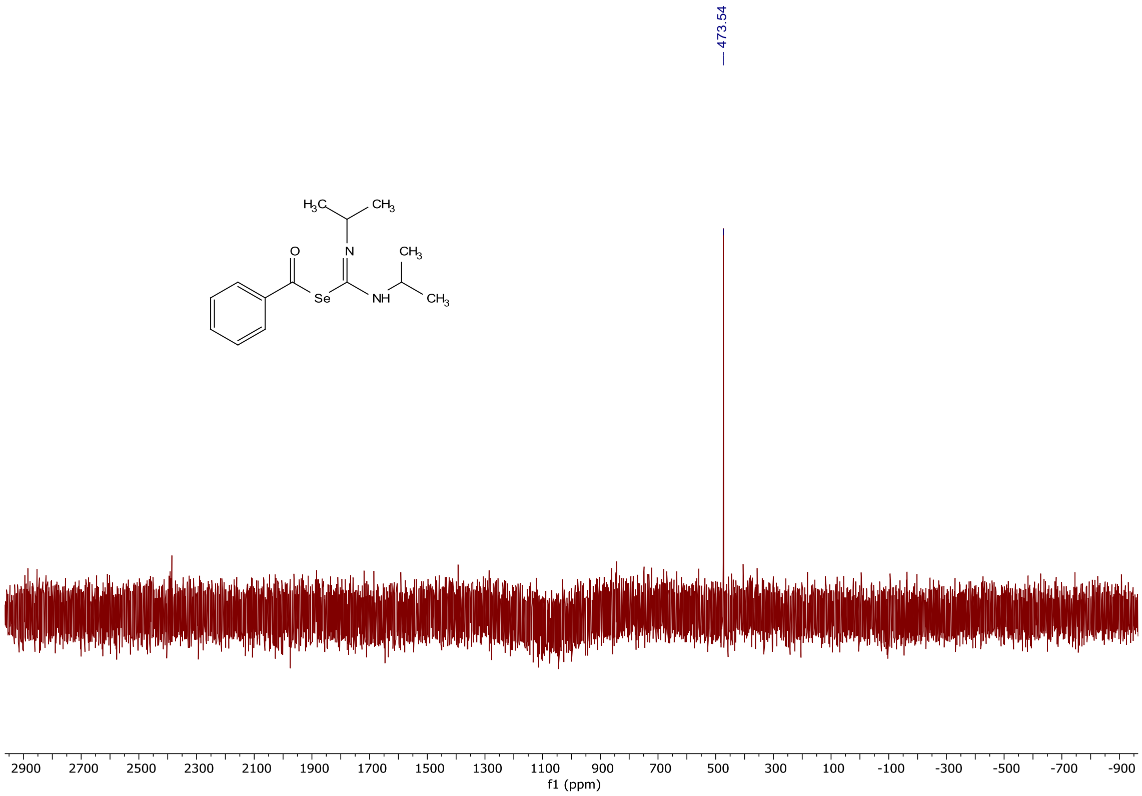

Figure S26. ^{77}Se-NMR spectrum of compound **3**.

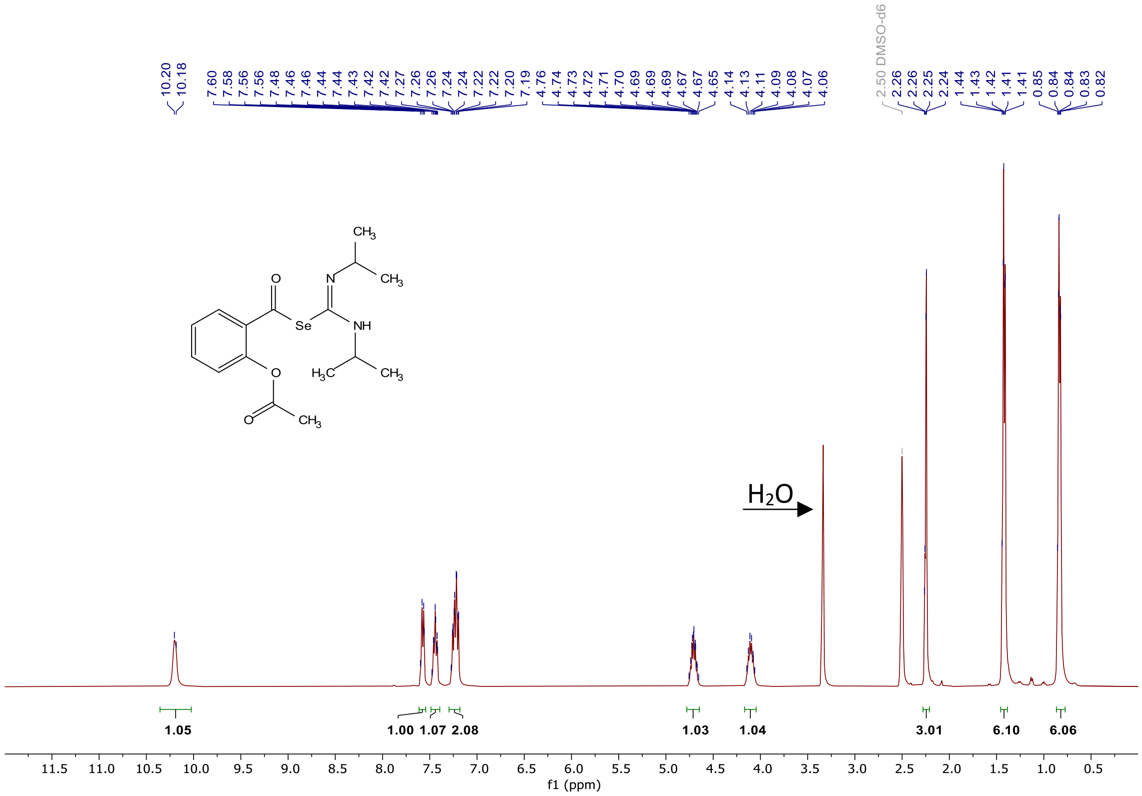

Figure S27. ^{1}H-NMR spectrum of compound **4**.

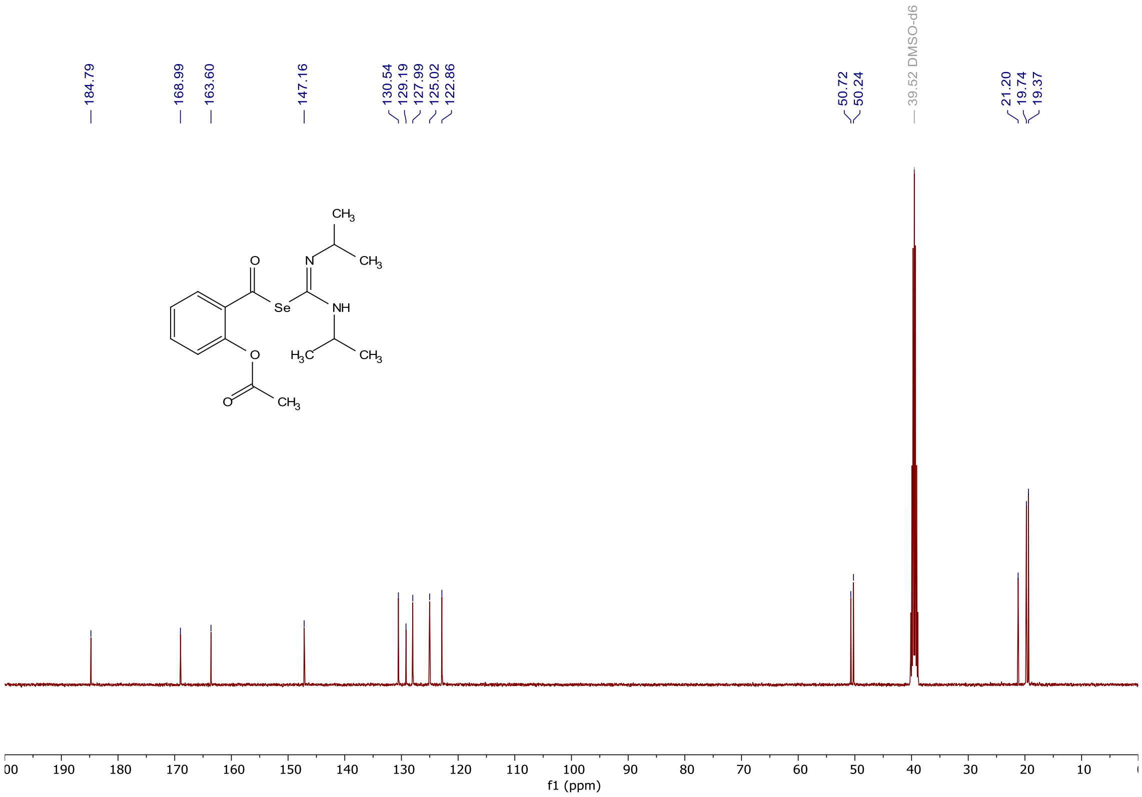

Figure S28. ^{13}C-NMR spectrum of compound **4**.

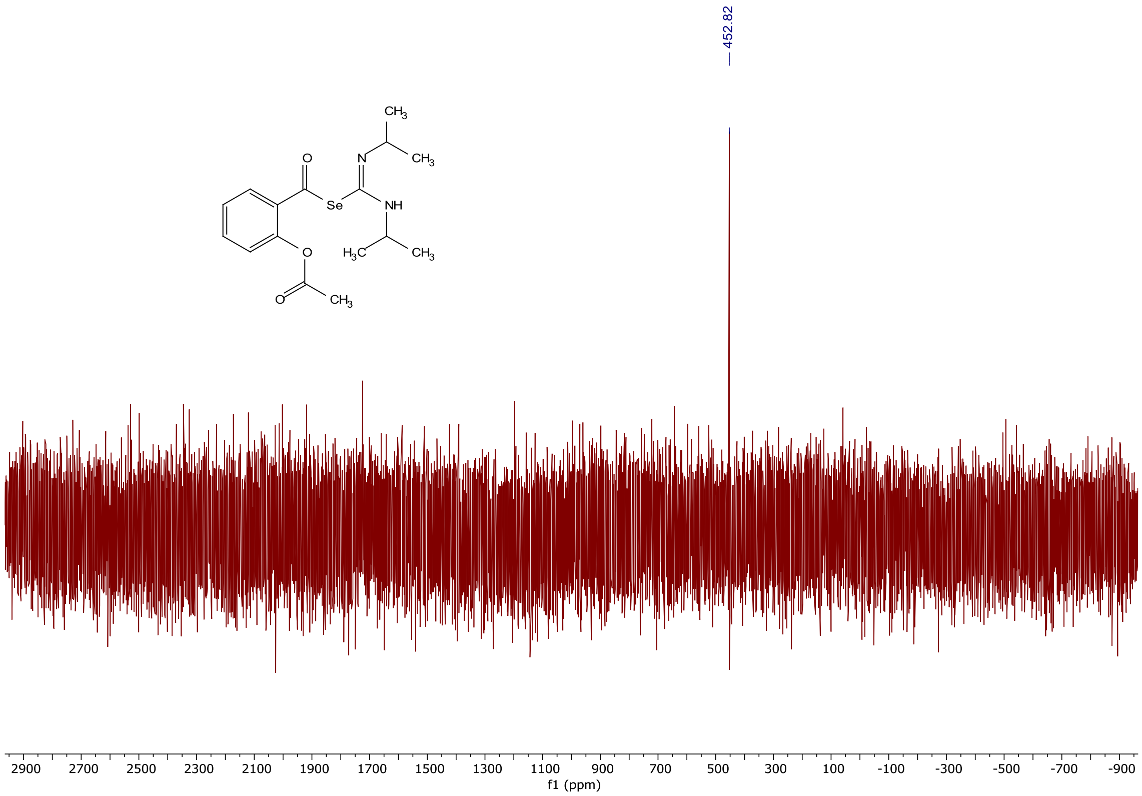

Figure S29. ^{77}Se-NMR spectrum of compound **4**.

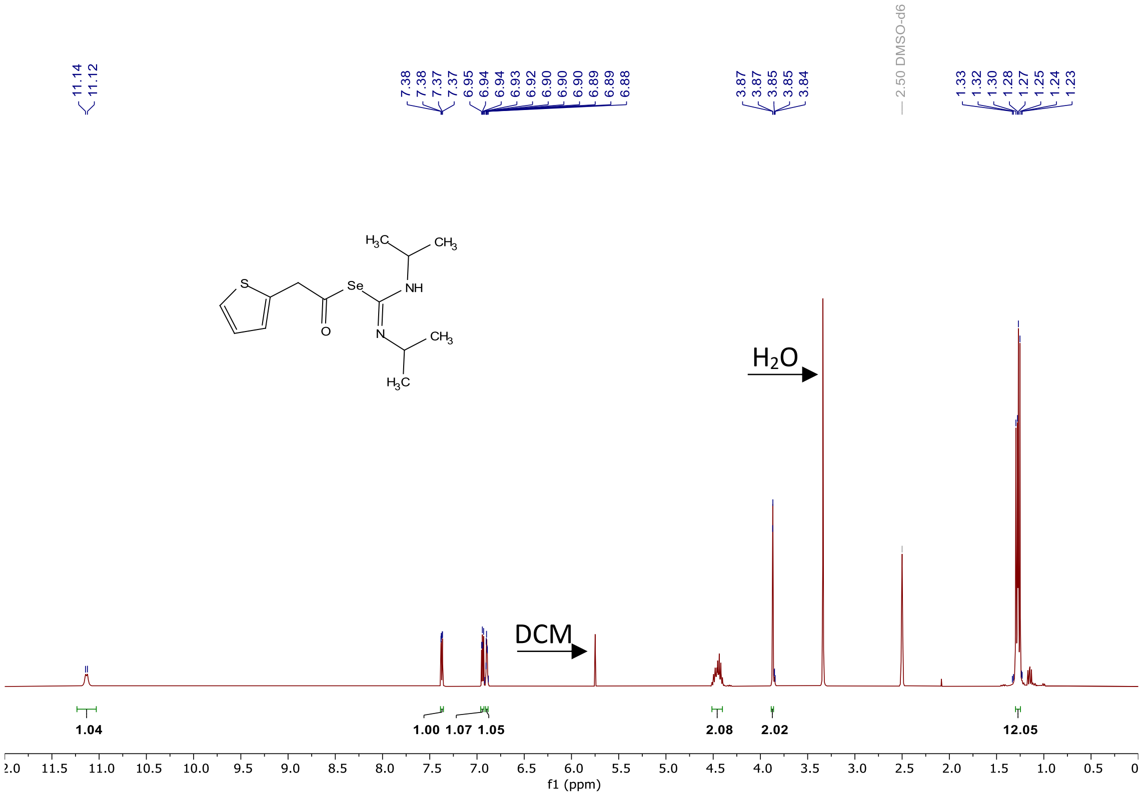

Figure S30. ^{1}H-NMR spectrum of compound **5**.

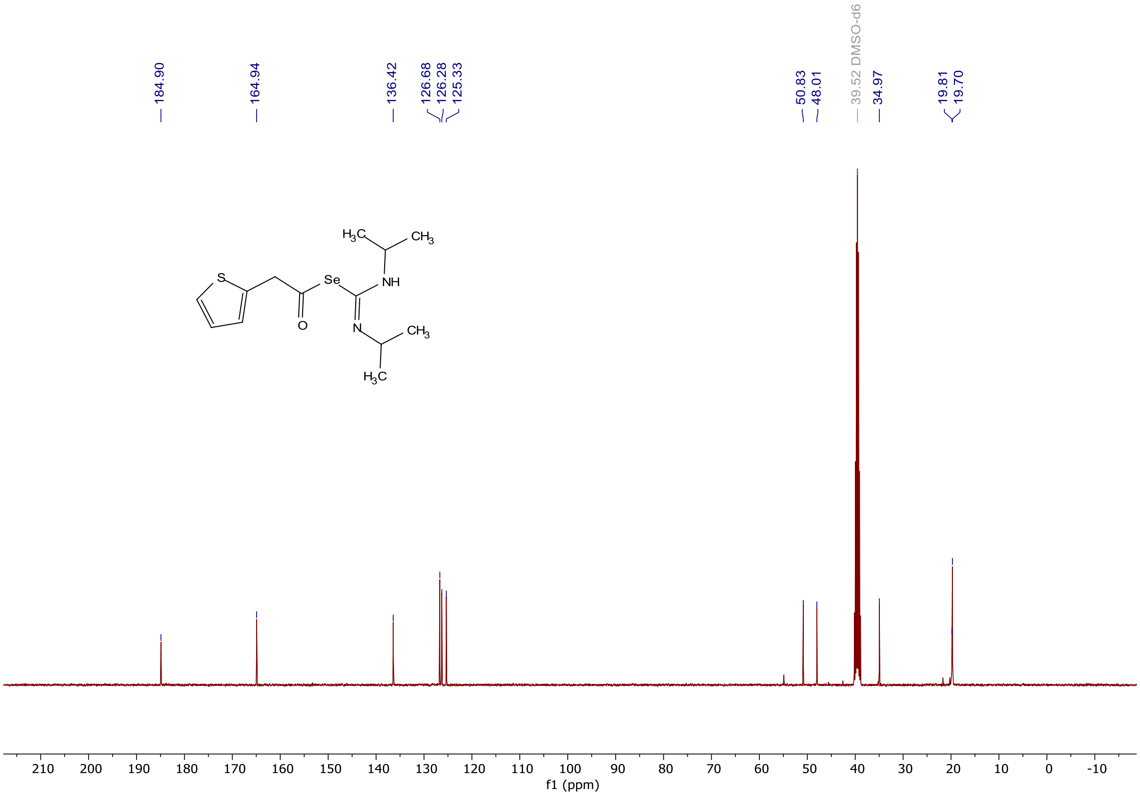

Figure S31. ^{13}C-NMR spectrum of compound **5**.

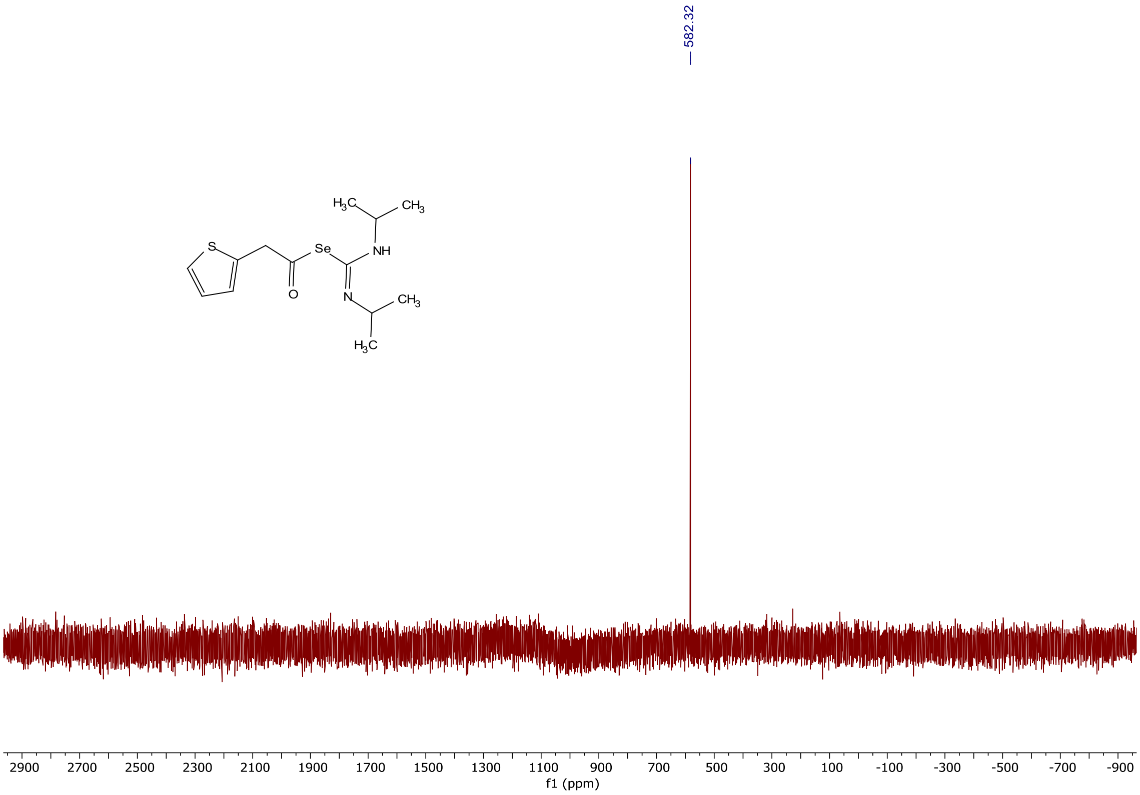

Figure S32. ^{77}Se-NMR spectrum of compound **5**.

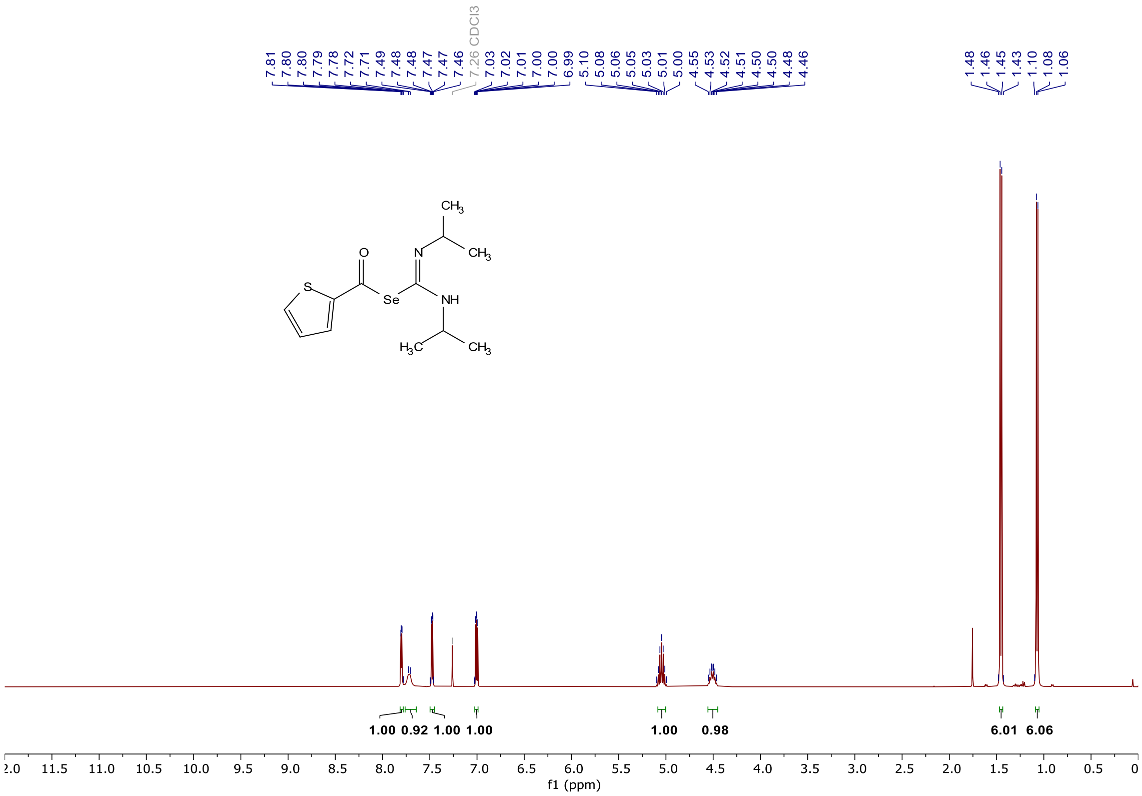

Figure S33. ^{1}H-NMR spectrum of compound **6**.

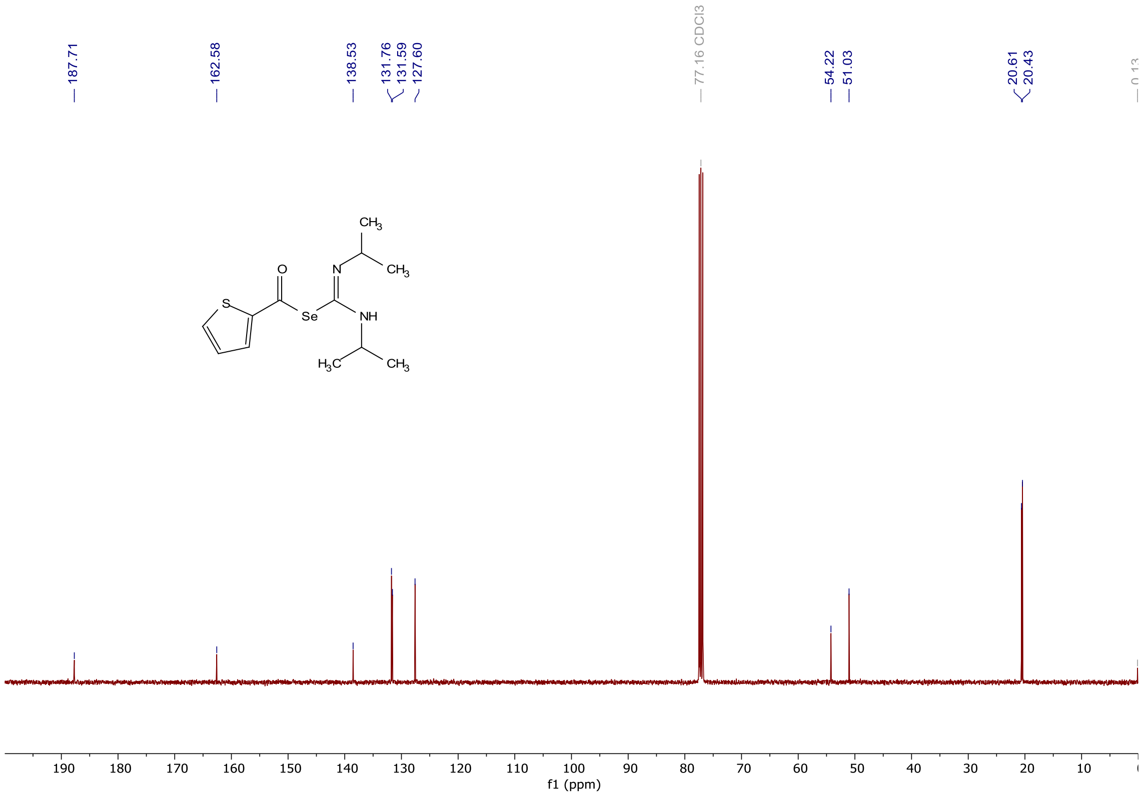

Figure S34. ^{13}C-NMR spectrum of compound **6**.

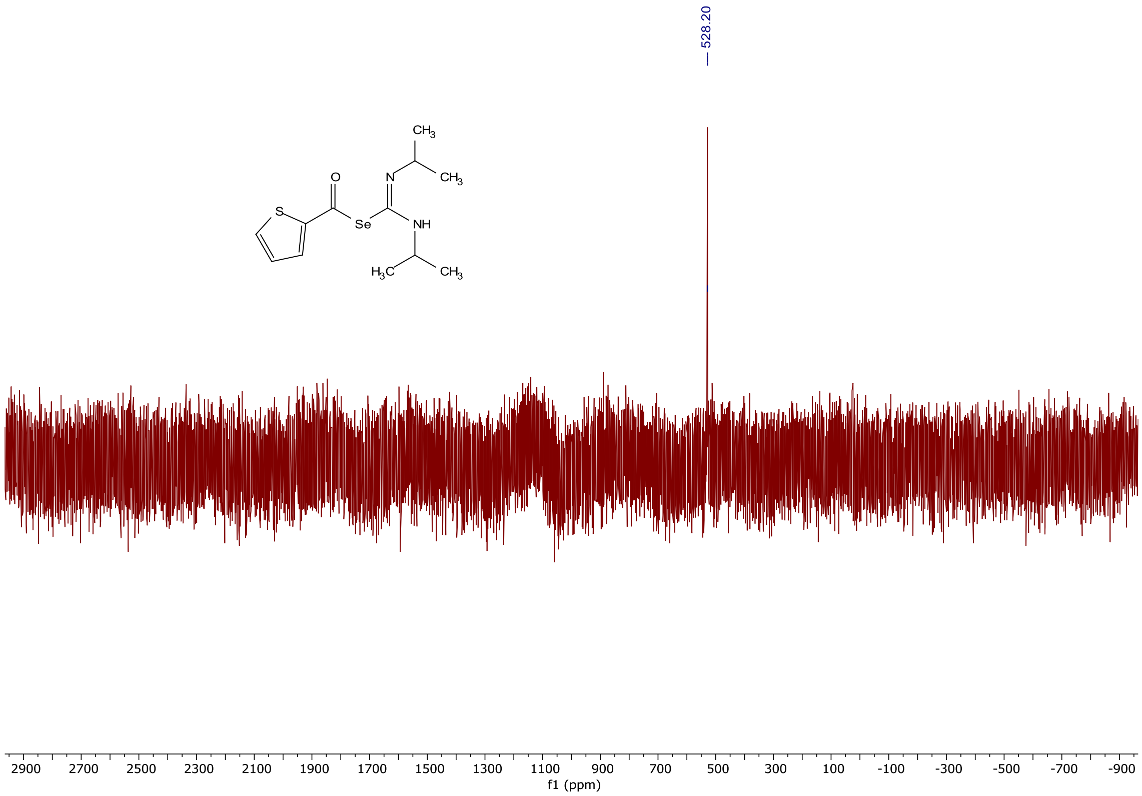

Figure S35. ^{77}Se-NMR spectrum of compound **6**.

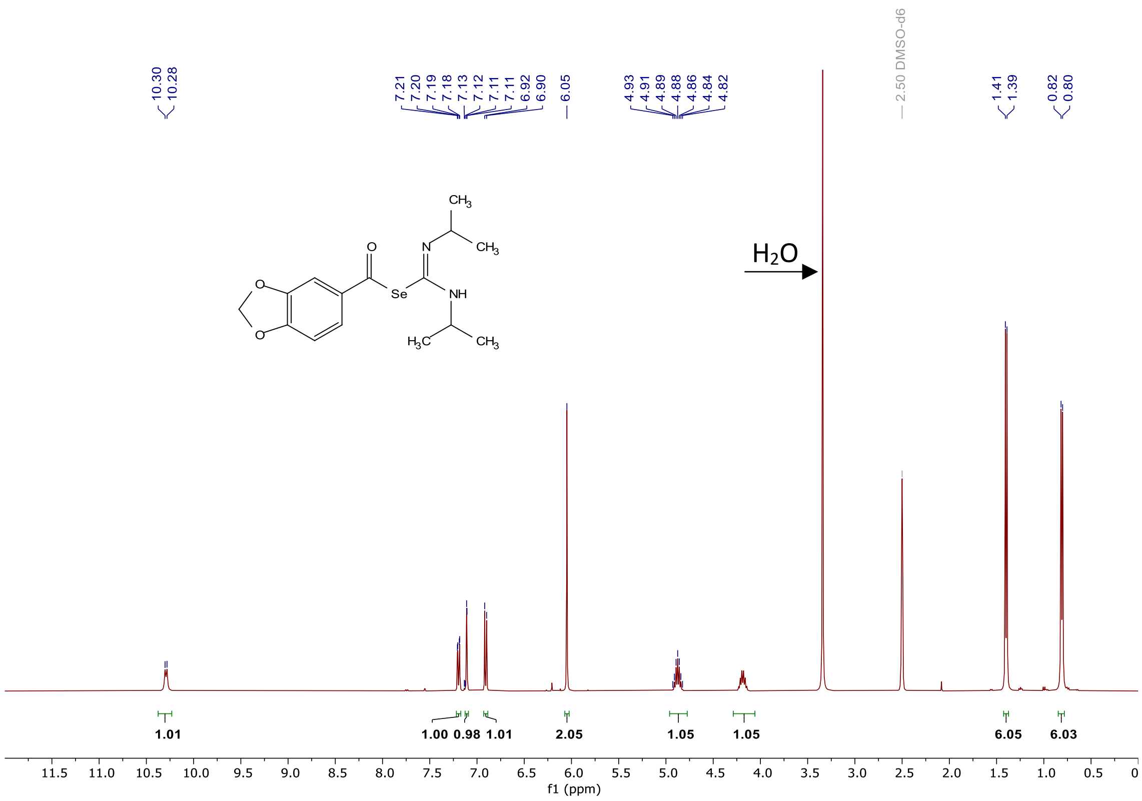

Figure S36. ^{1}H-NMR spectrum of compound **7**.

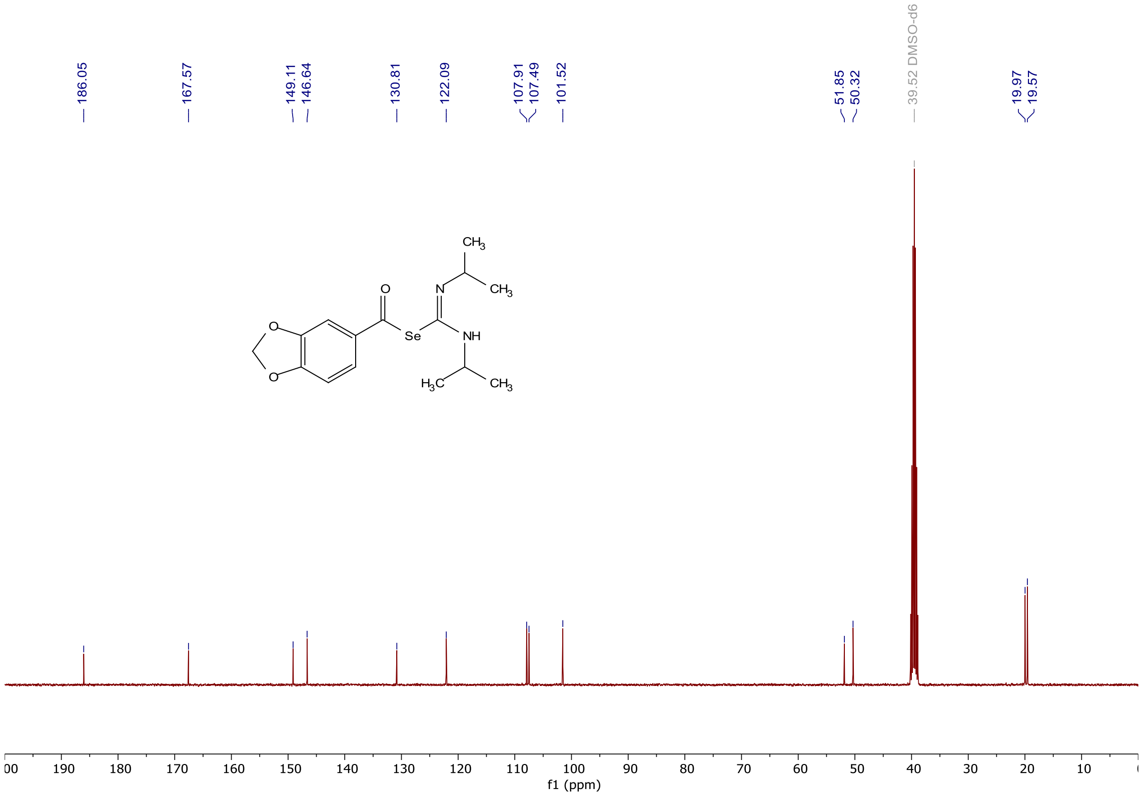

Figure S37. ^{13}C-NMR spectrum of compound **7**.

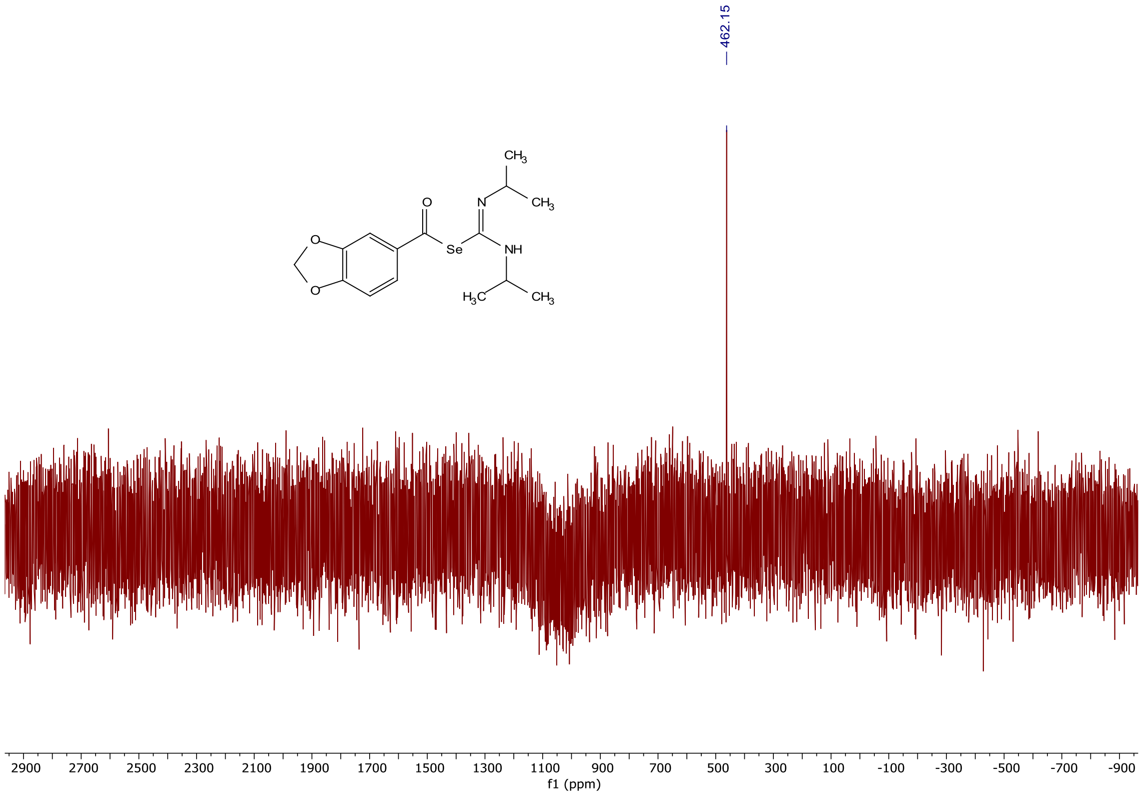

Figure S38. ^{77}Se-NMR spectrum of compound **7**.

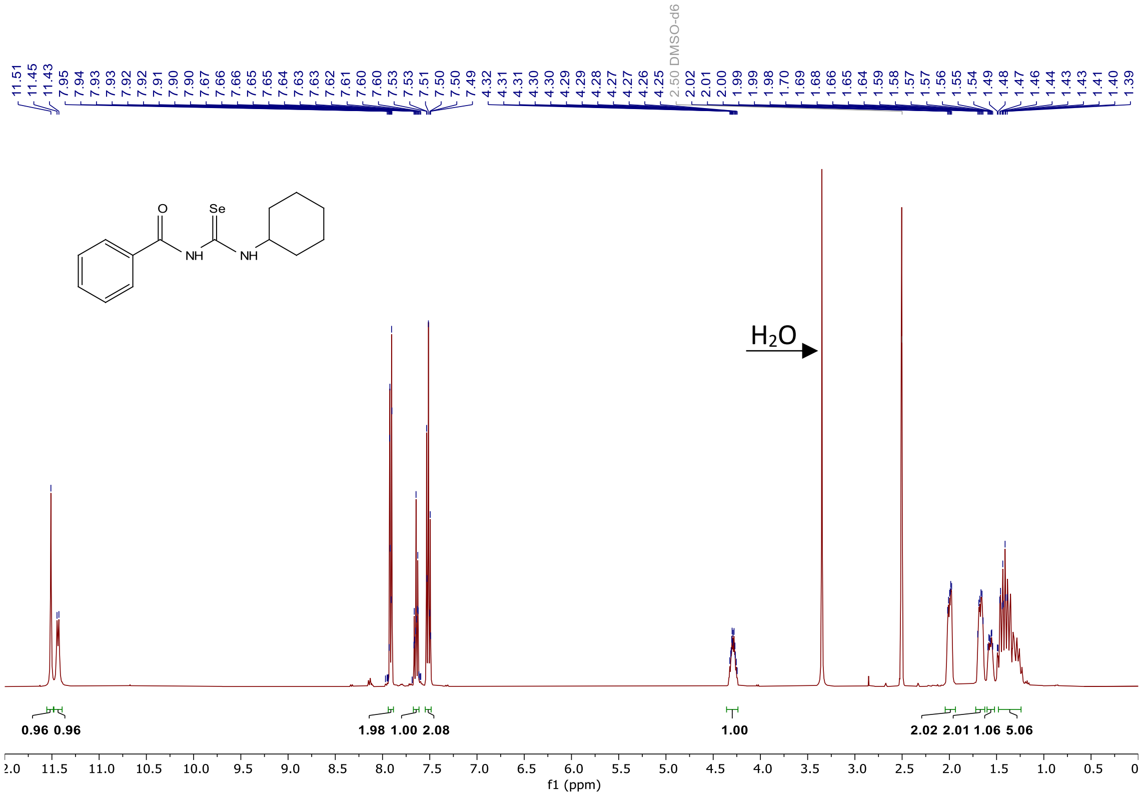

Figure S39. ^{1}H-NMR spectrum of compound **8**.

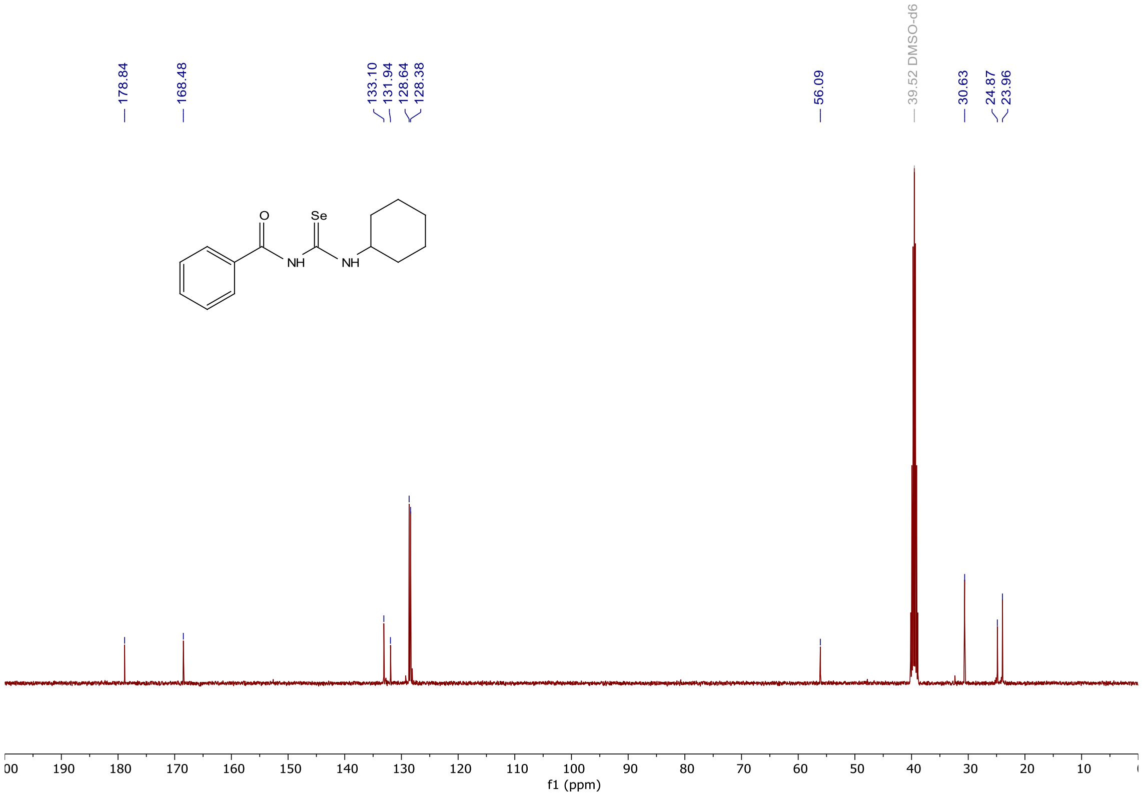

Figure S40. ^{13}C-NMR spectrum of compound **8**.

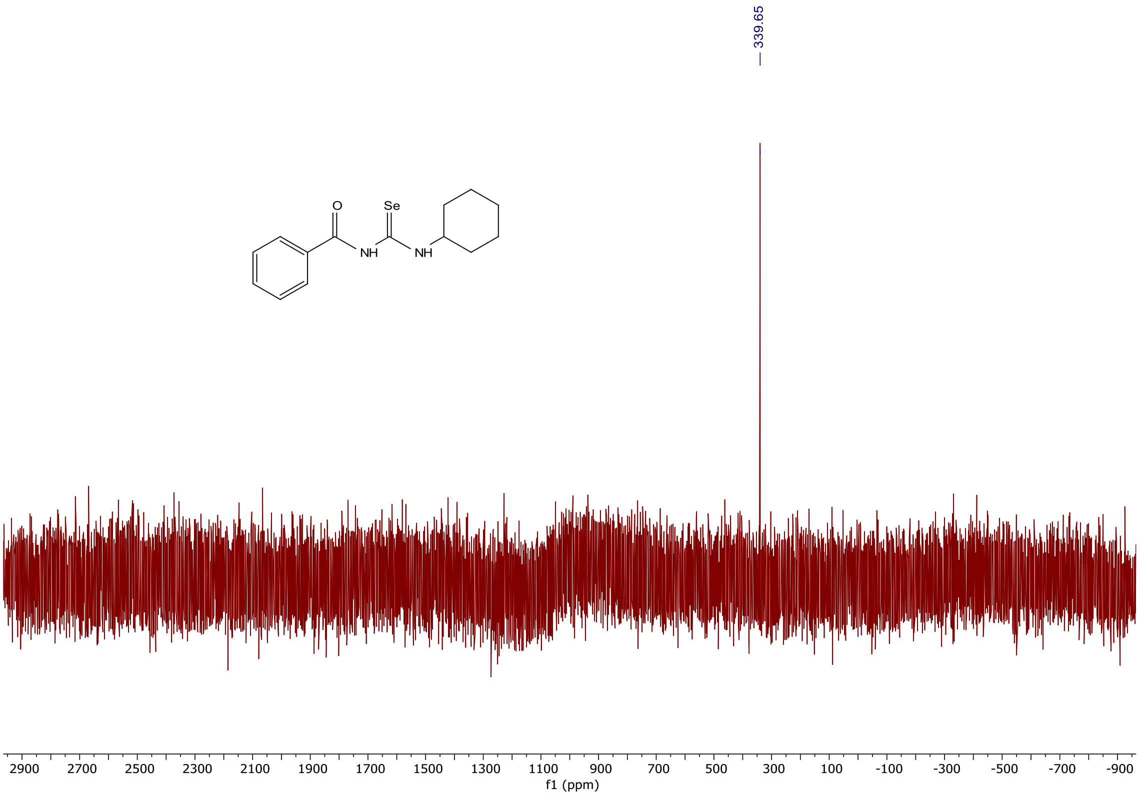

Figure S41. ^{77}Se-NMR spectrum of compound **8**.

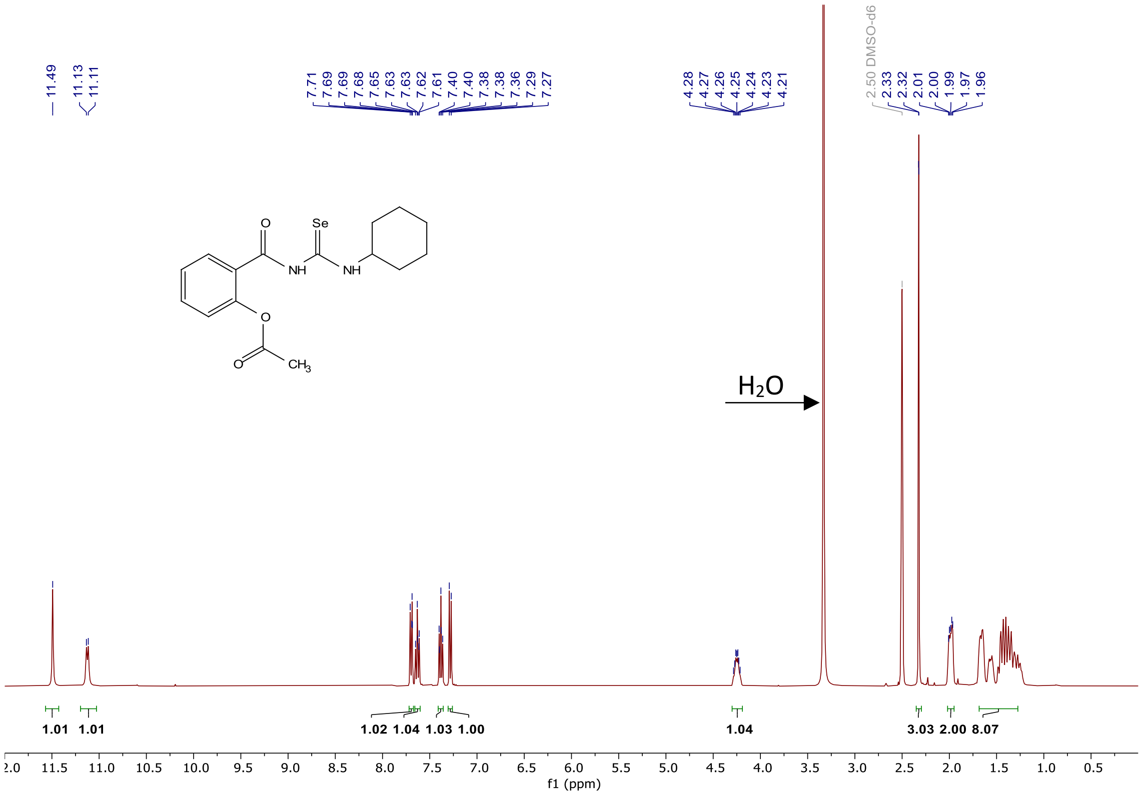

Figure S42. ^{1}H-NMR spectrum of compound **9**.

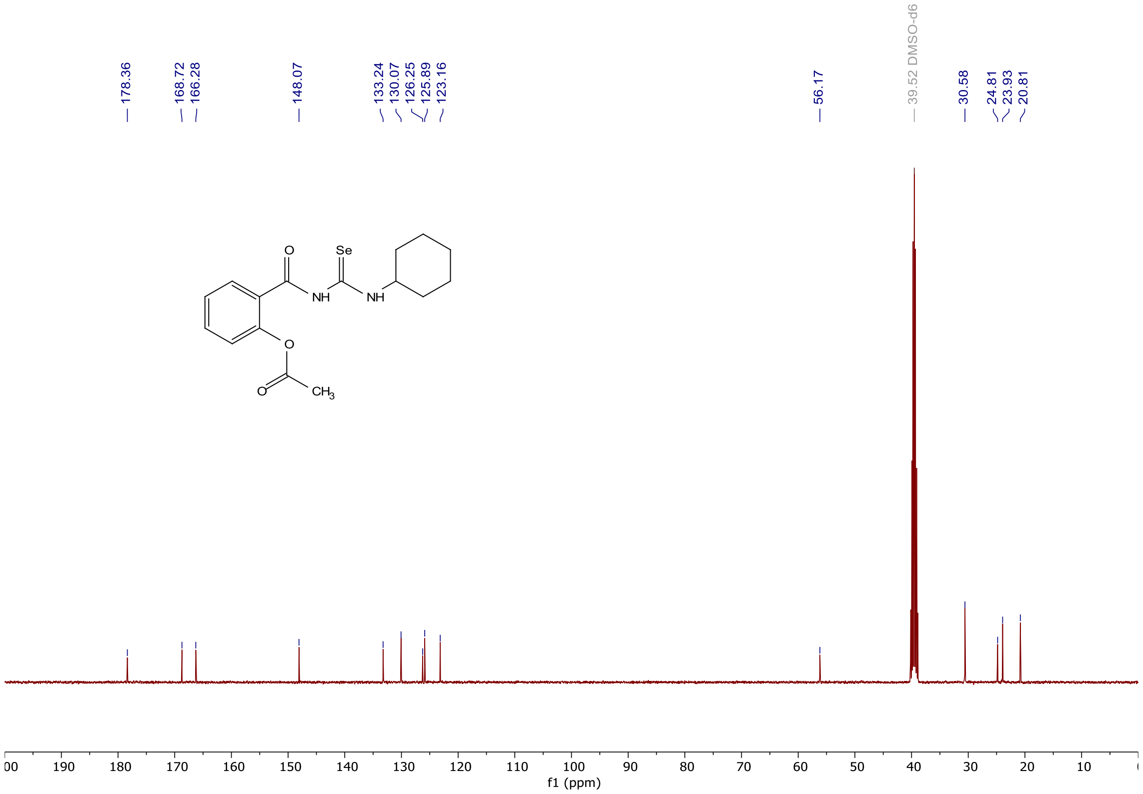

Figure S43. ^{13}C-NMR spectrum of compound **9**.

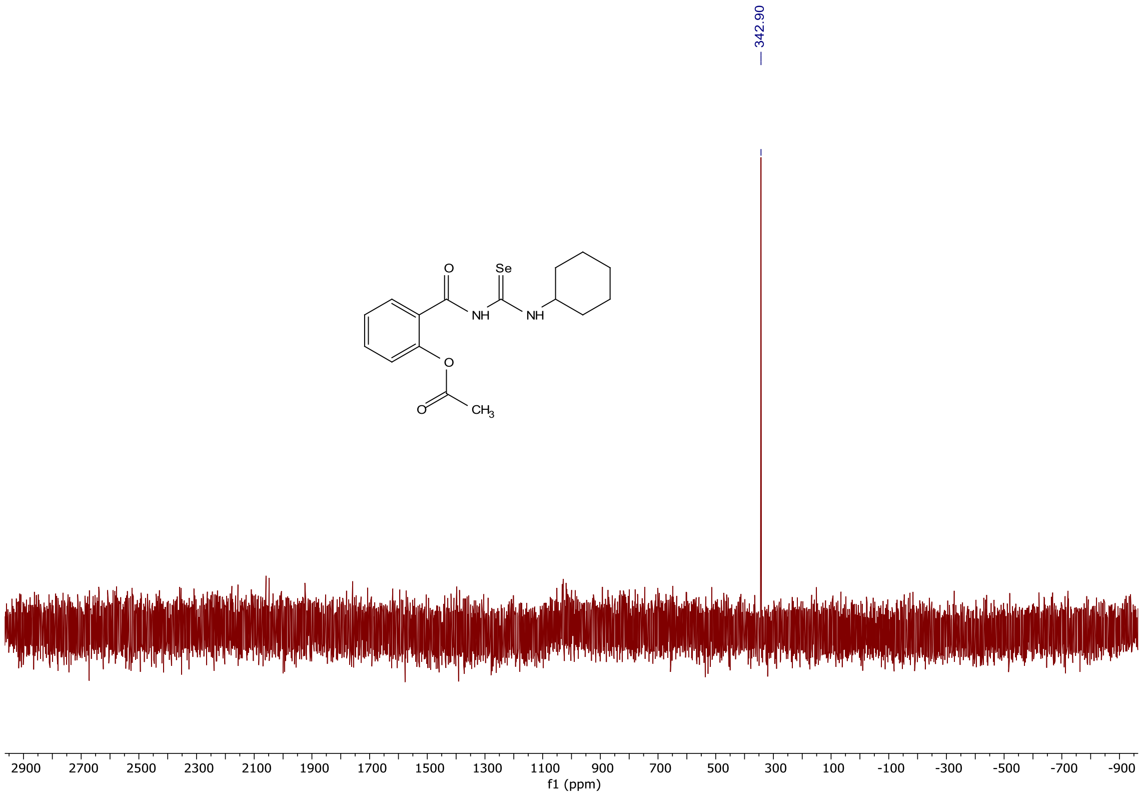

Figure S44. ^{77}Se-NMR spectrum of compound **9**.

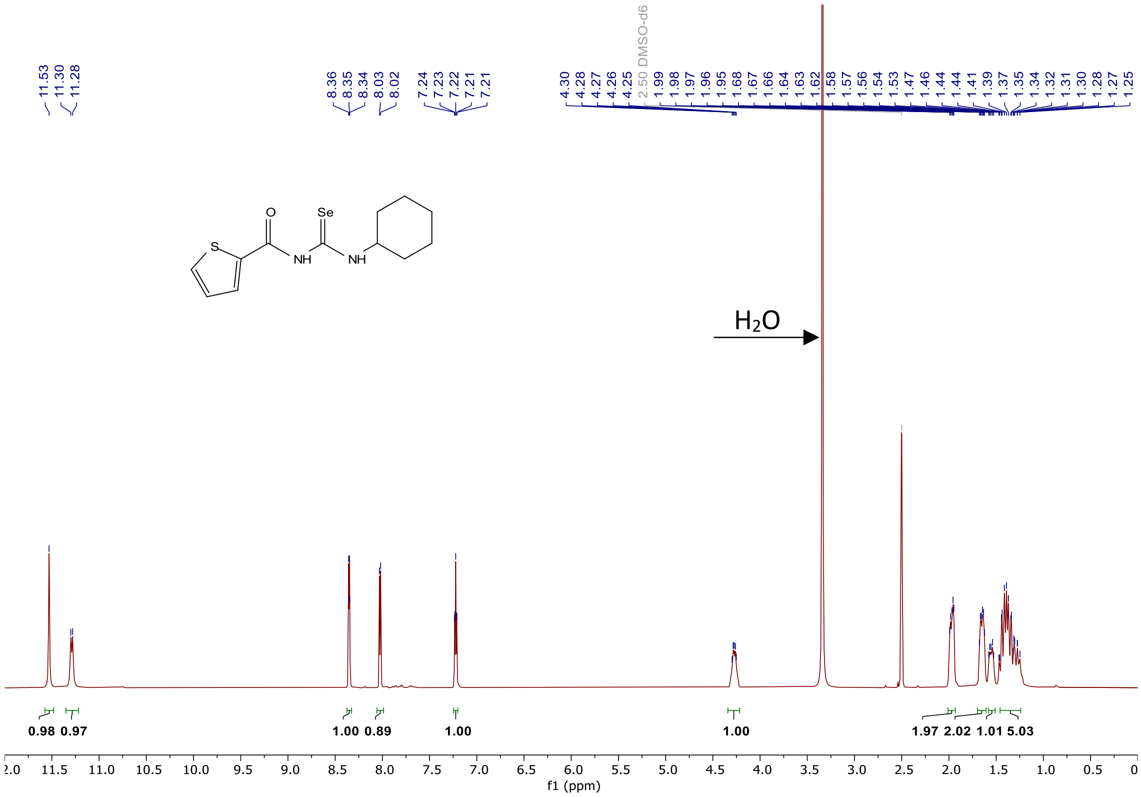

Figure S45. ^{1}H-NMR spectrum of compound **10**.

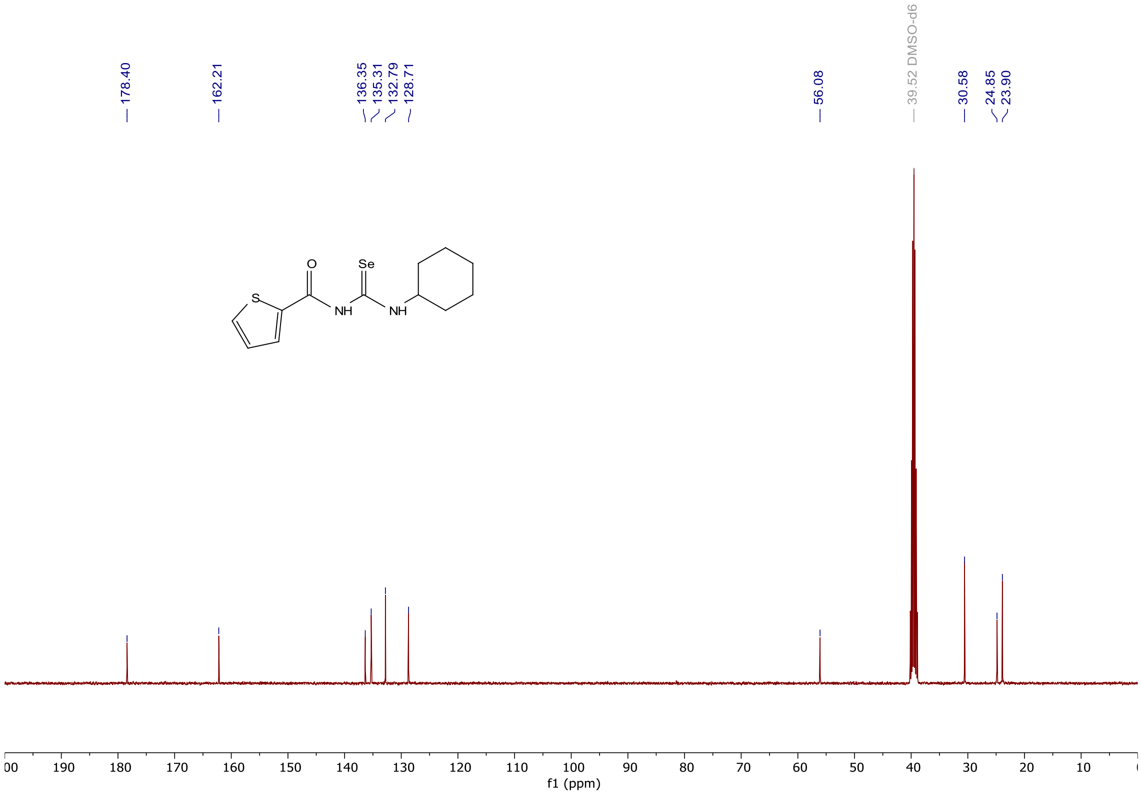

Figure S46. ^{13}C-NMR spectrum of compound **10**.

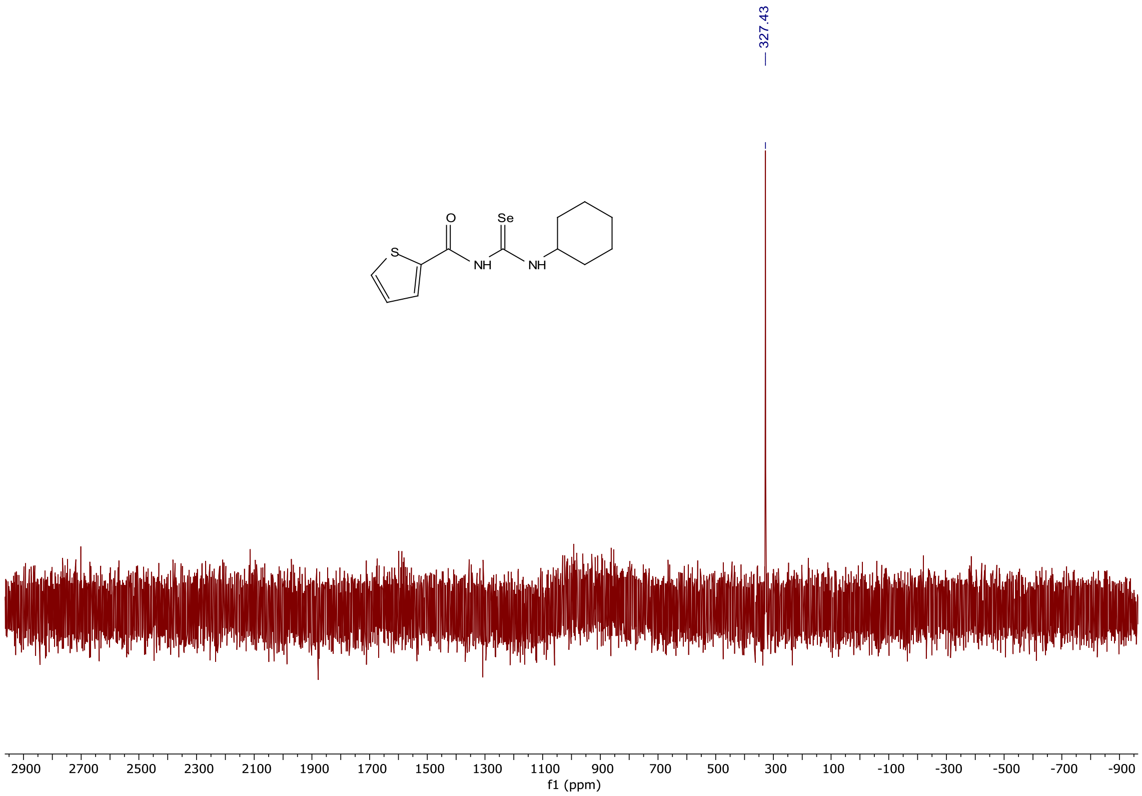

Figure S47. ^{77}Se-NMR spectrum of compound **10**.

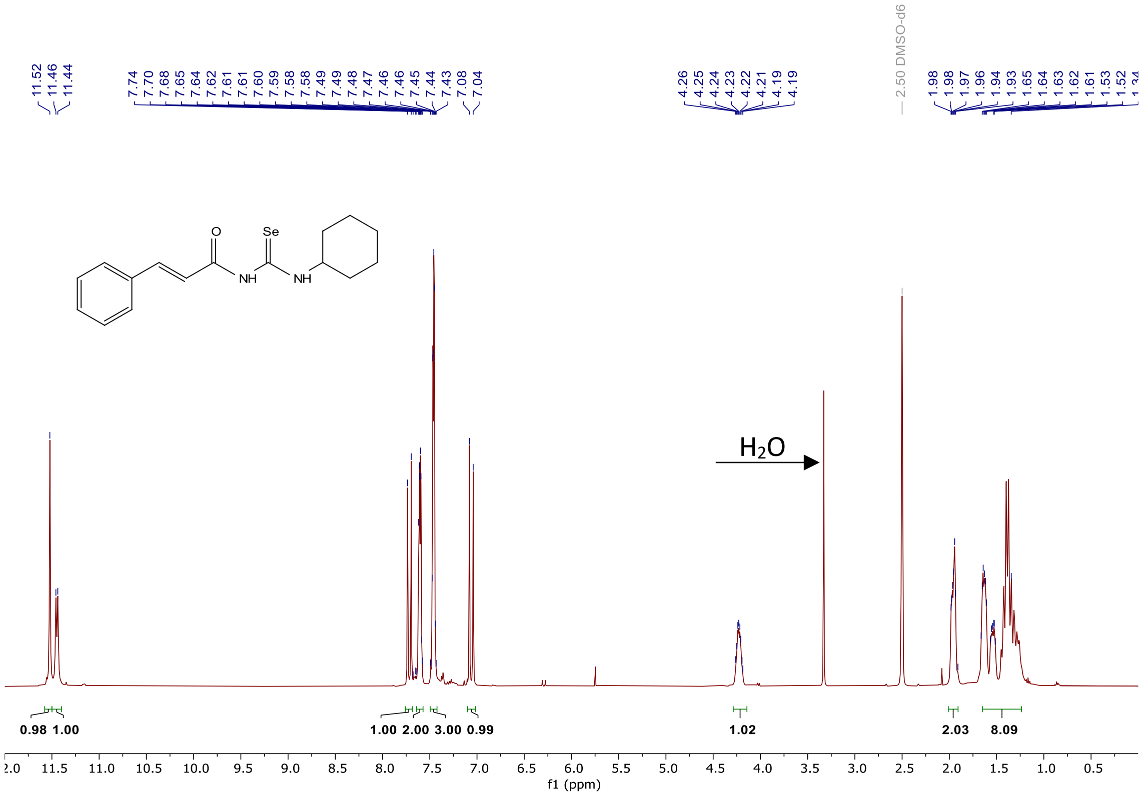

Figure S48. ^{1}H-NMR spectrum of compound **11**.

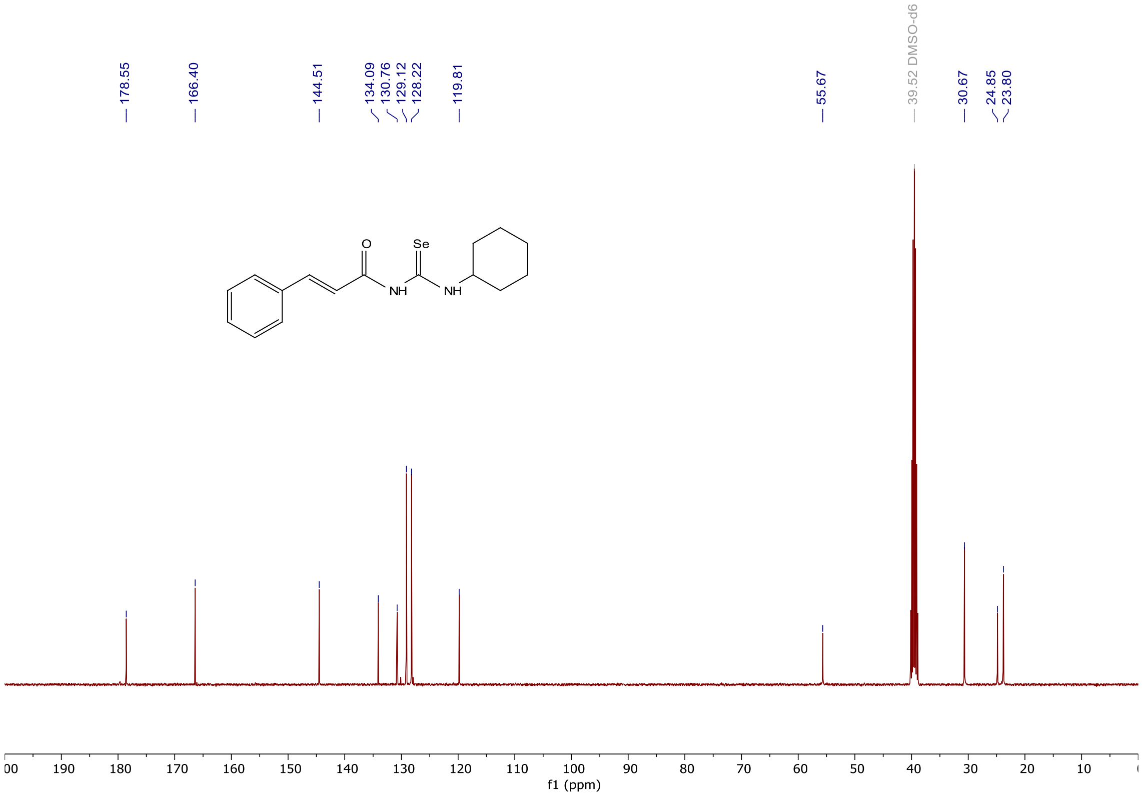

Figure S49. ^{13}C-NMR spectrum of compound **11**.

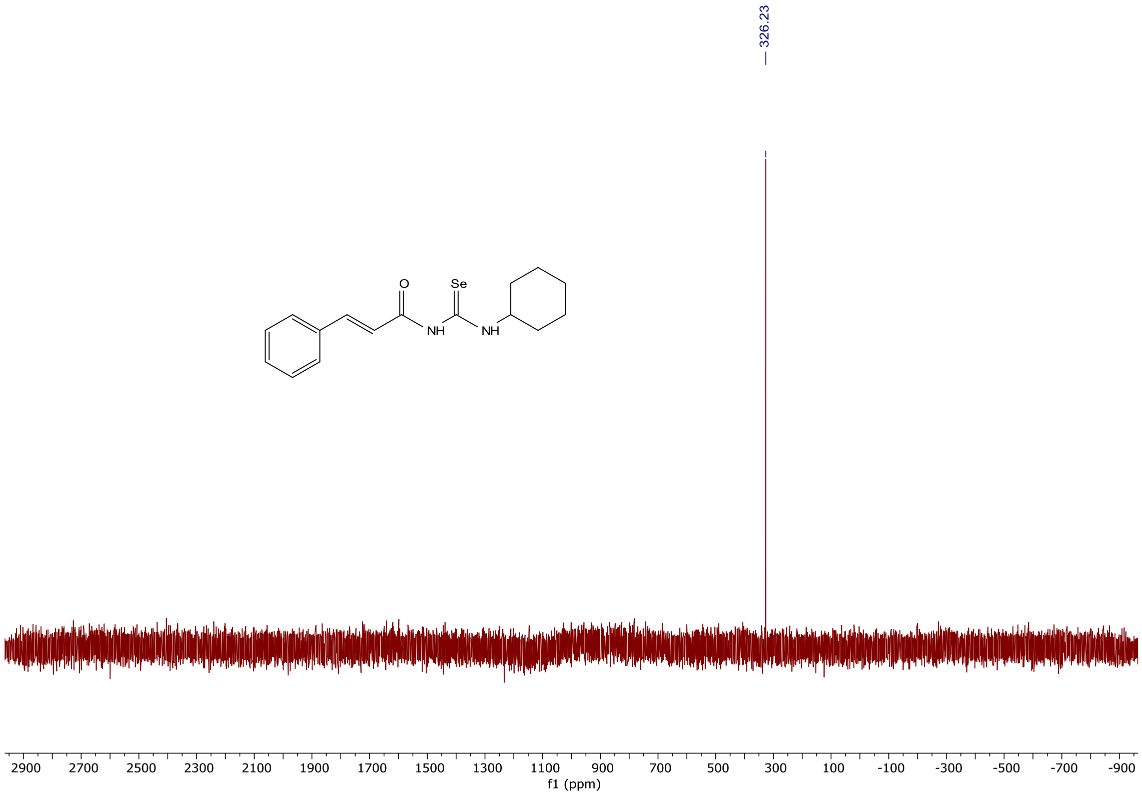

Figure S50. ^{77}Se-NMR spectrum of compound **11**.

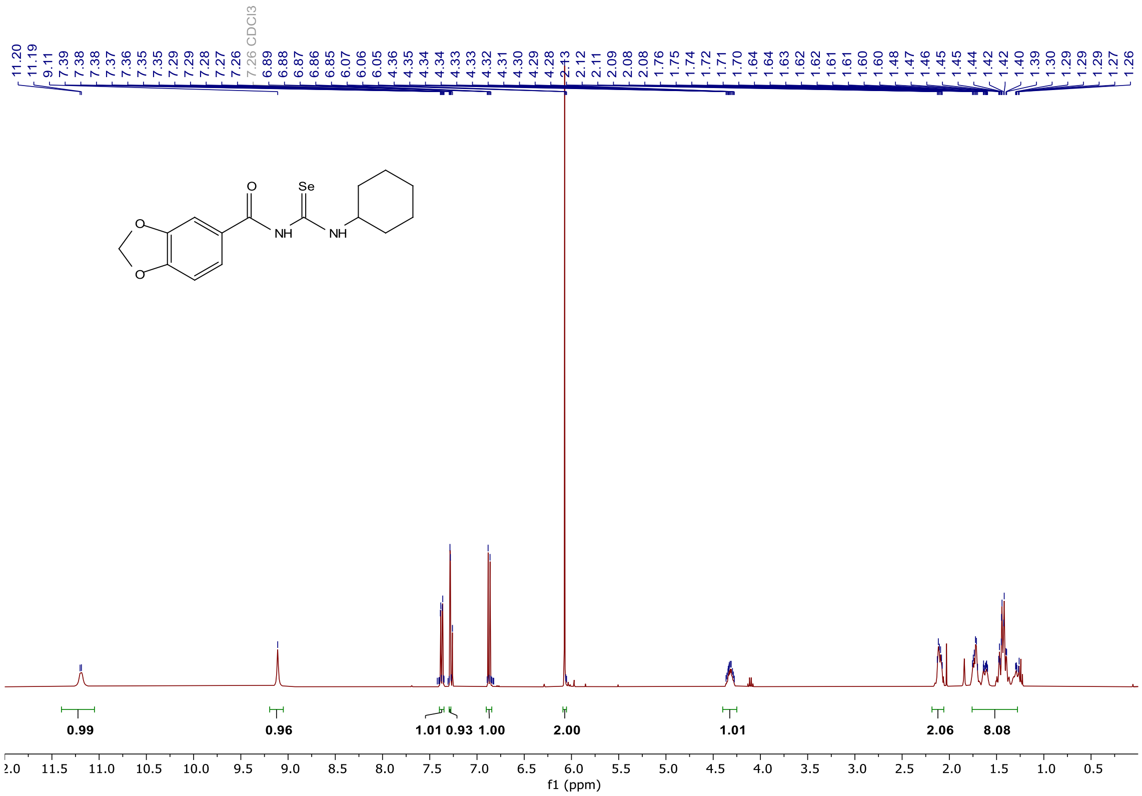

Figure S51. ^{1}H-NMR spectrum of compound **12**.

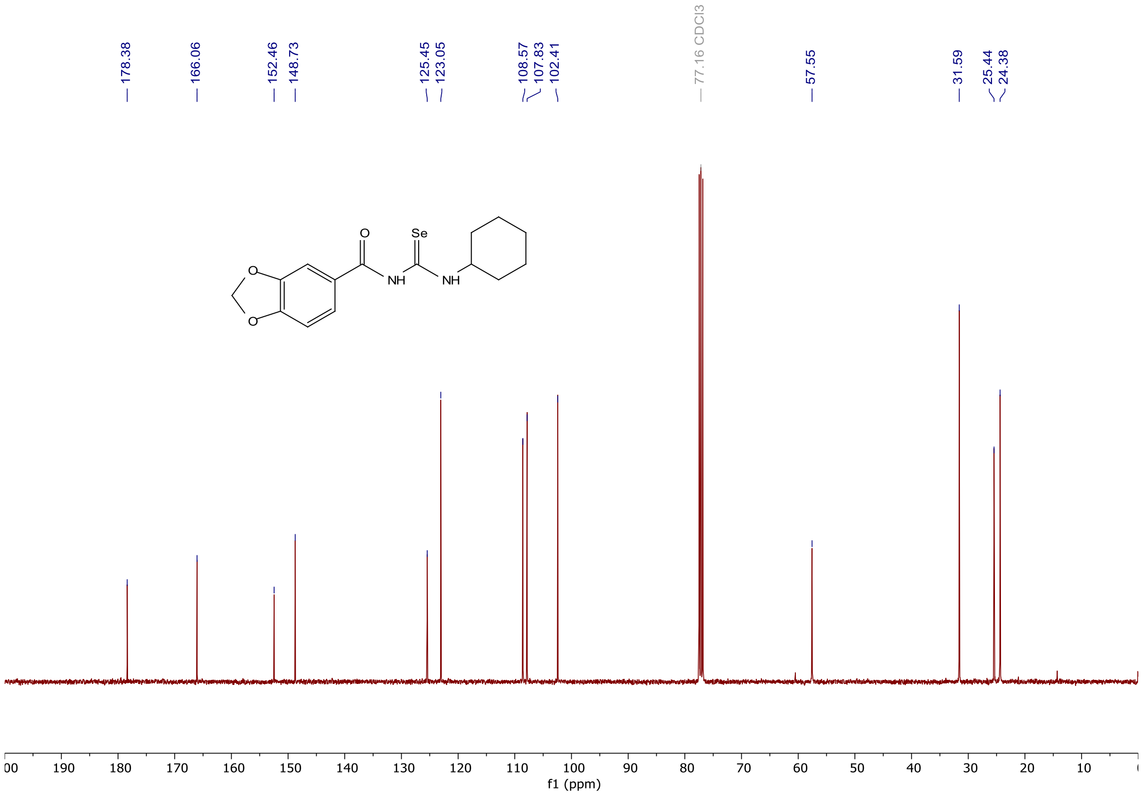

Figure S52. ^{13}C-NMR spectrum of compound **12**.

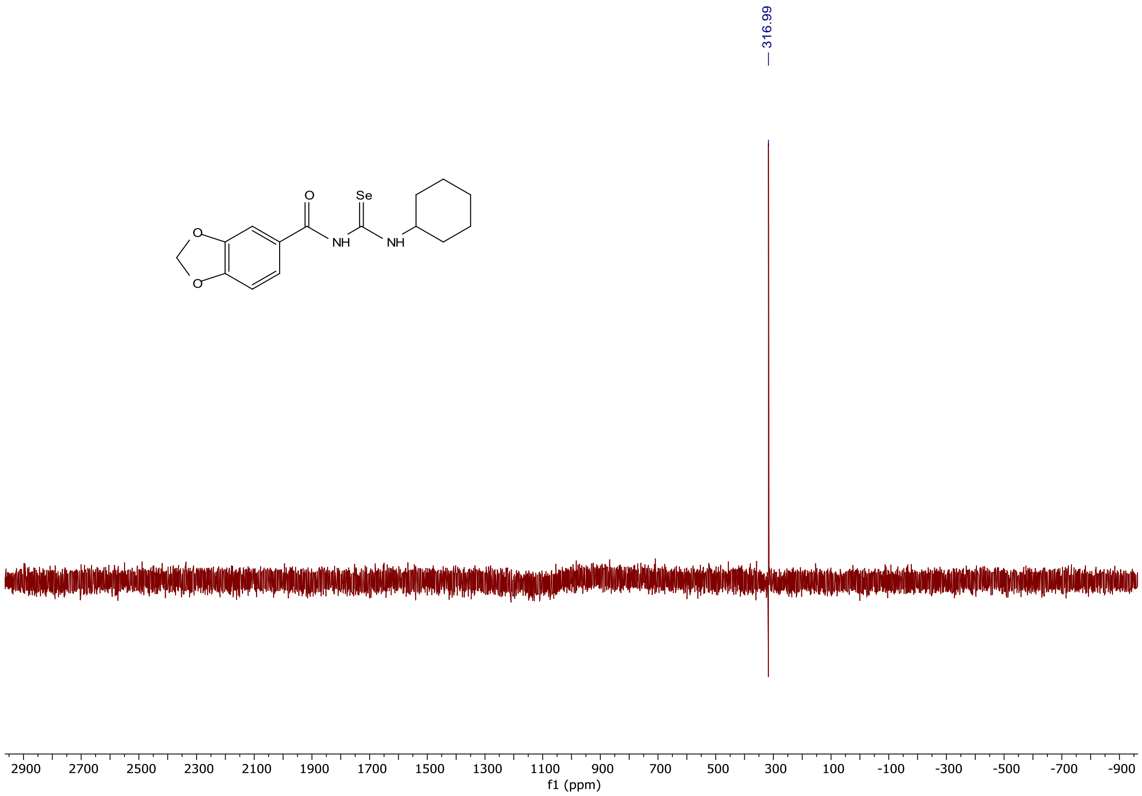

Figure S53. ^{77}Se-NMR spectrum of compound **12**.

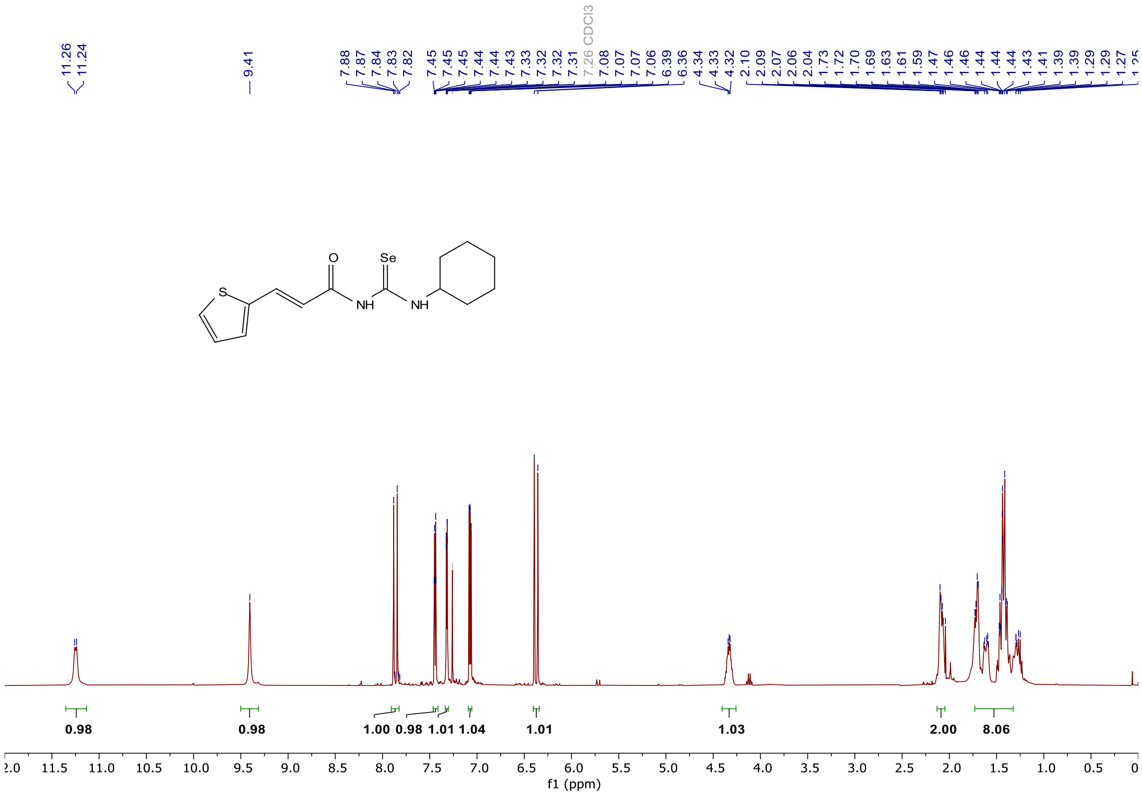

Figure S54. ^{1}H-NMR spectrum of compound **13**.

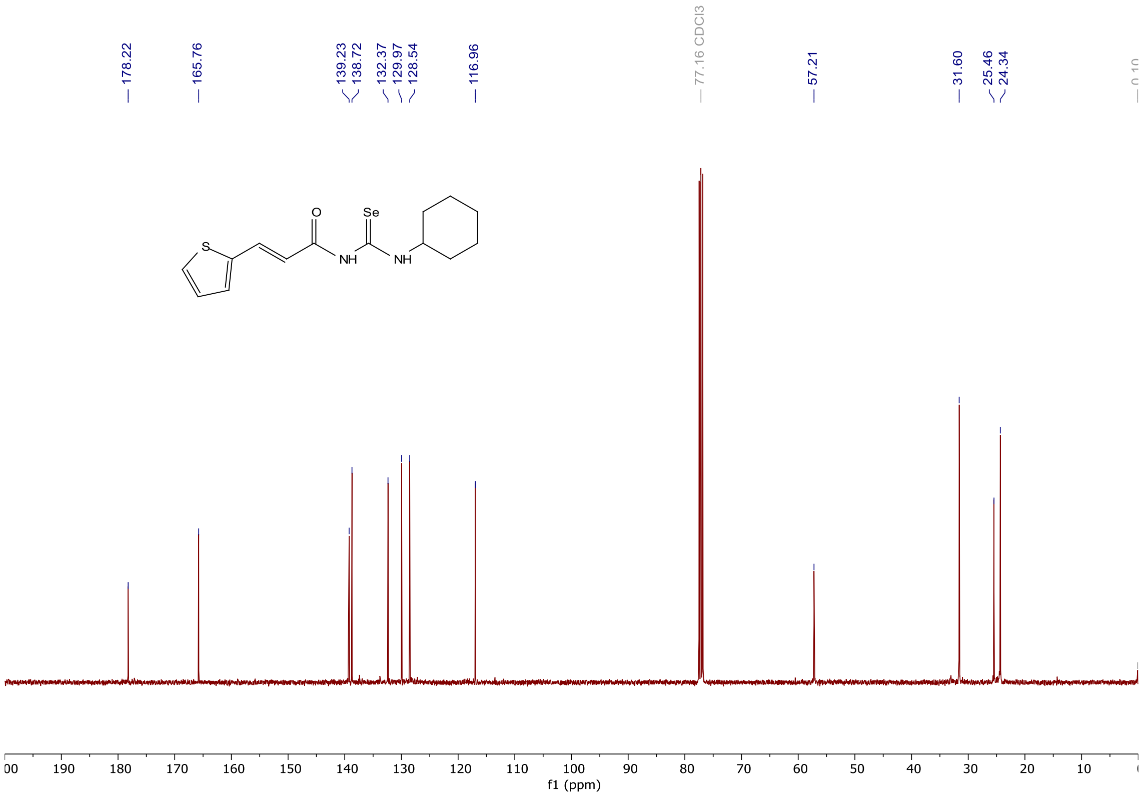

Figure S55. ^{13}C-NMR spectrum of compound **13**.

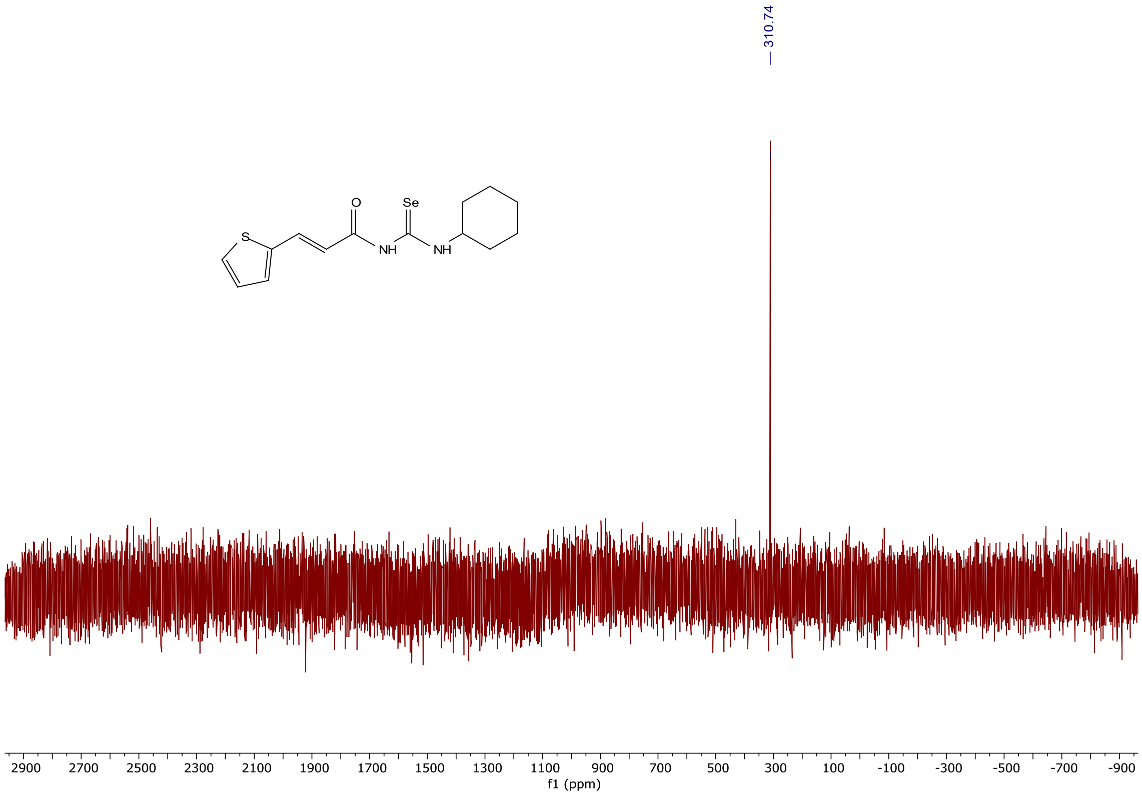

Figure S56. ^{7}Se-NMR spectrum of compound **13**.

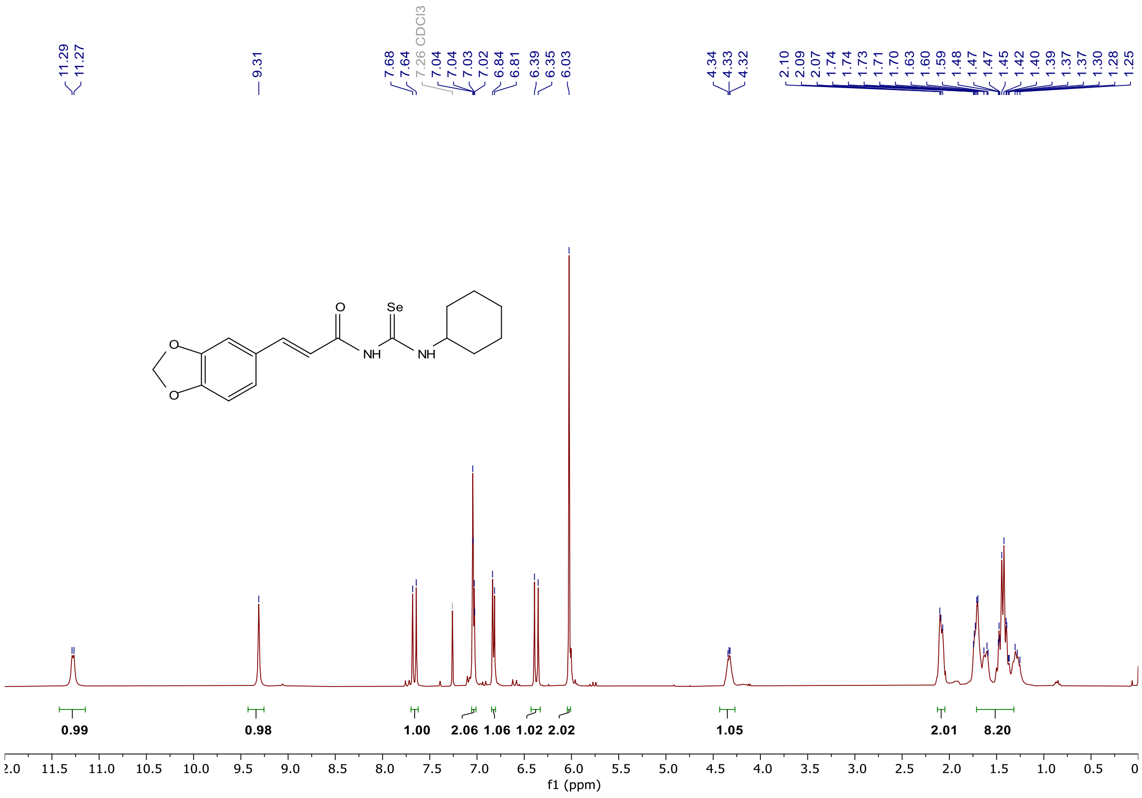

Figure S57. ^{1}H-NMR spectrum of compound **14**.

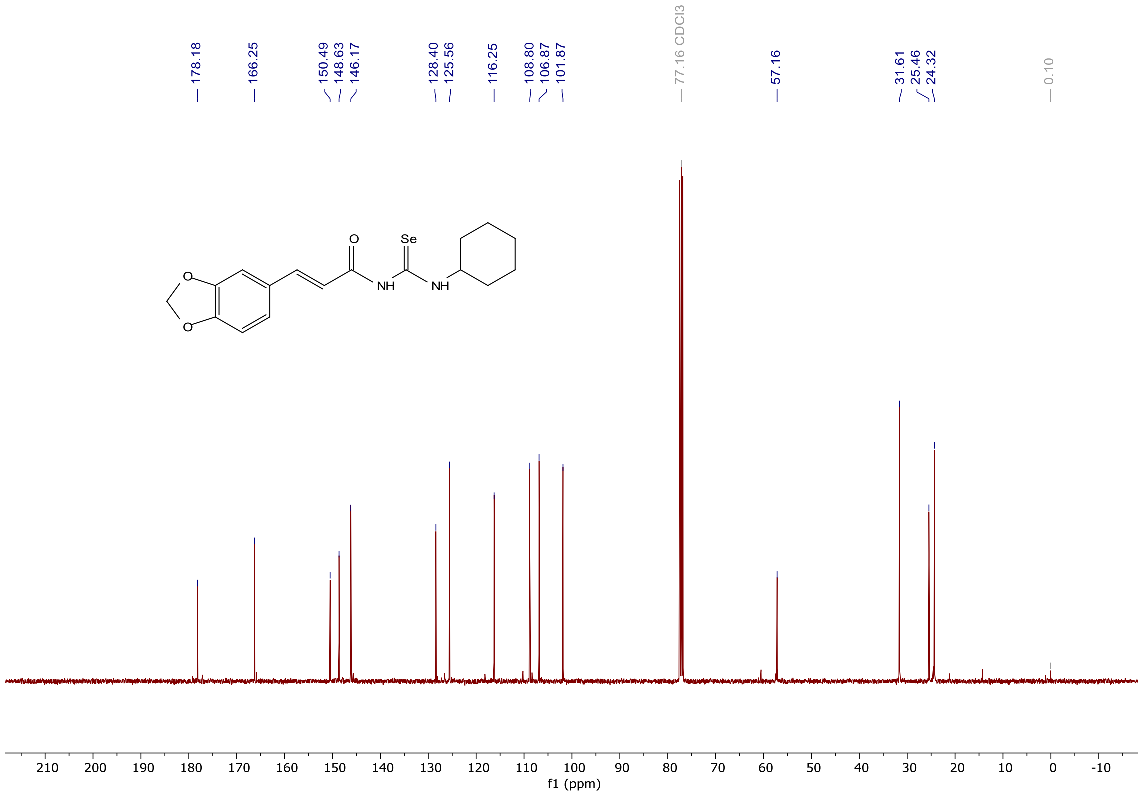

Figure S58. ^{13}C-NMR spectrum of compound **14**.

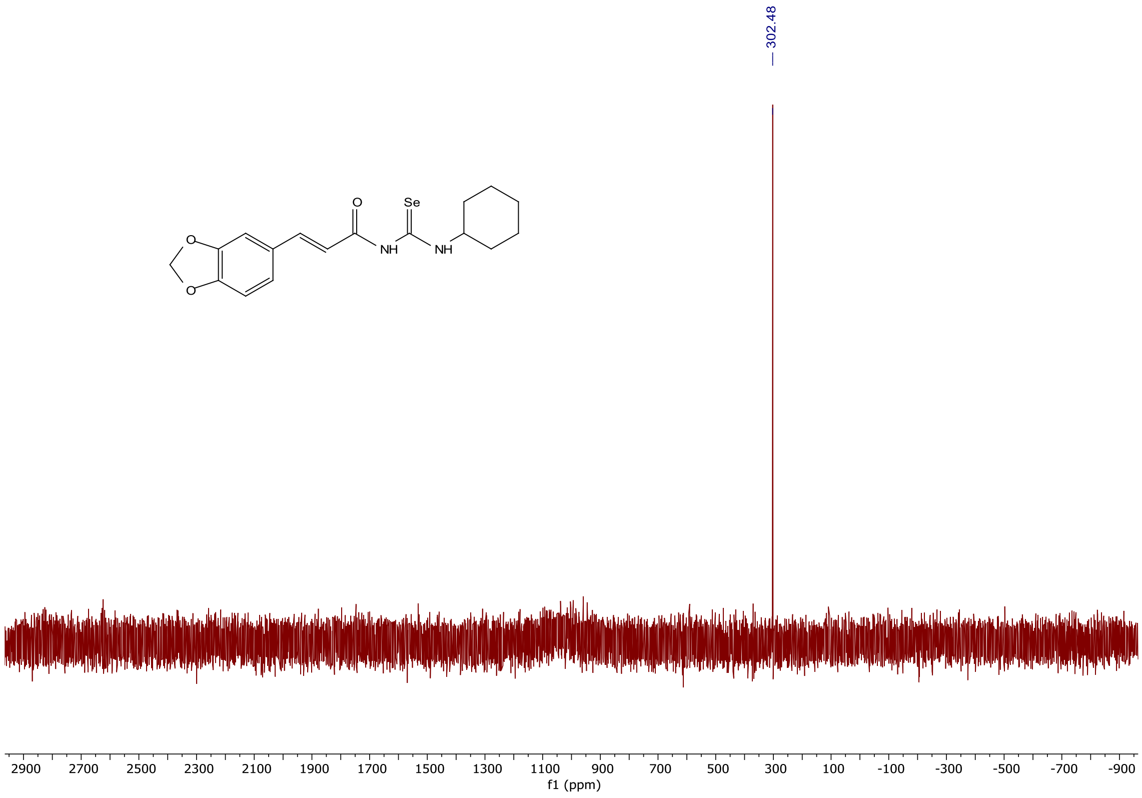

Figure S59. ^{77}Se-NMR spectrum of compound **14**.

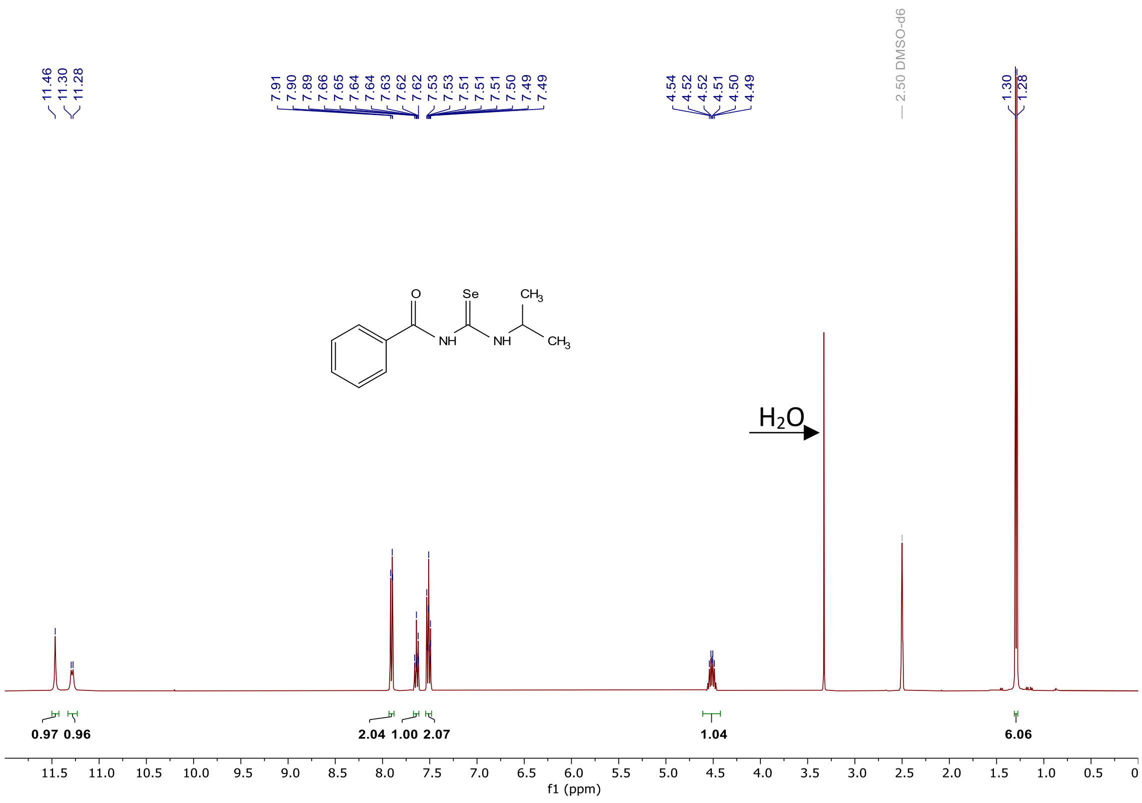

Figure S60. ^{1}H-NMR spectrum of compound **15**.

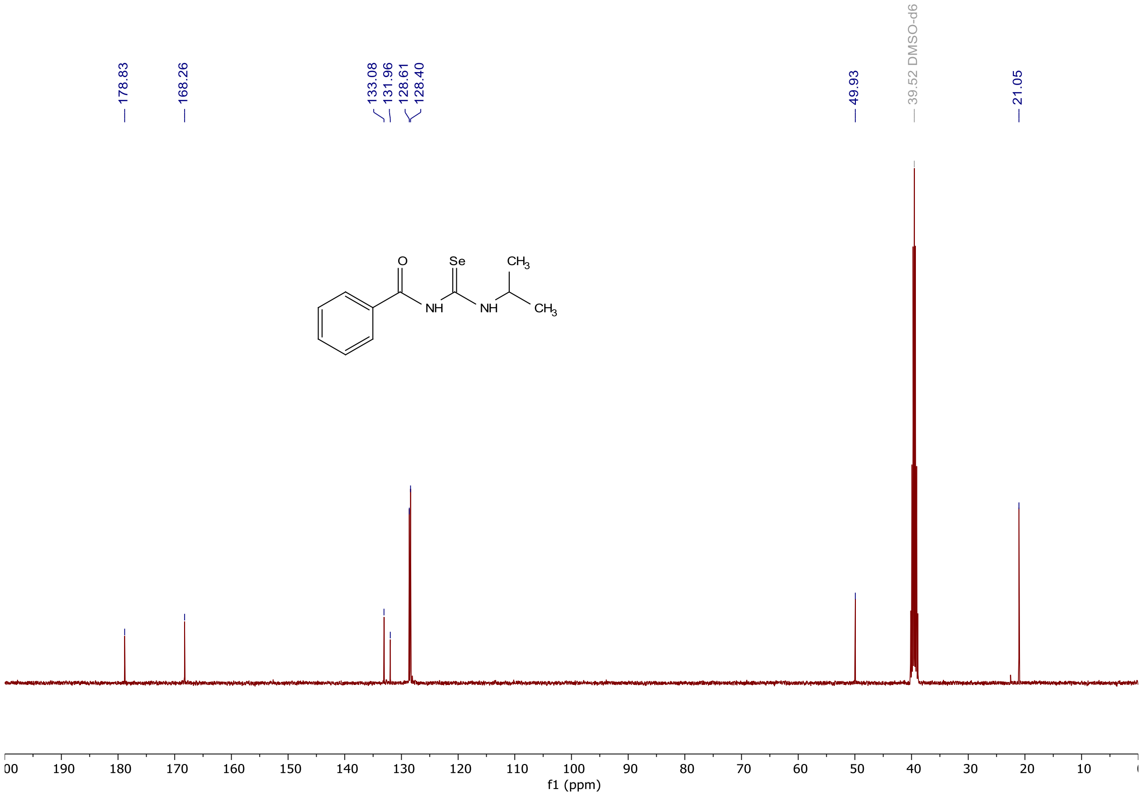

Figure S61. ^{13}C-NMR spectrum of compound **15**.

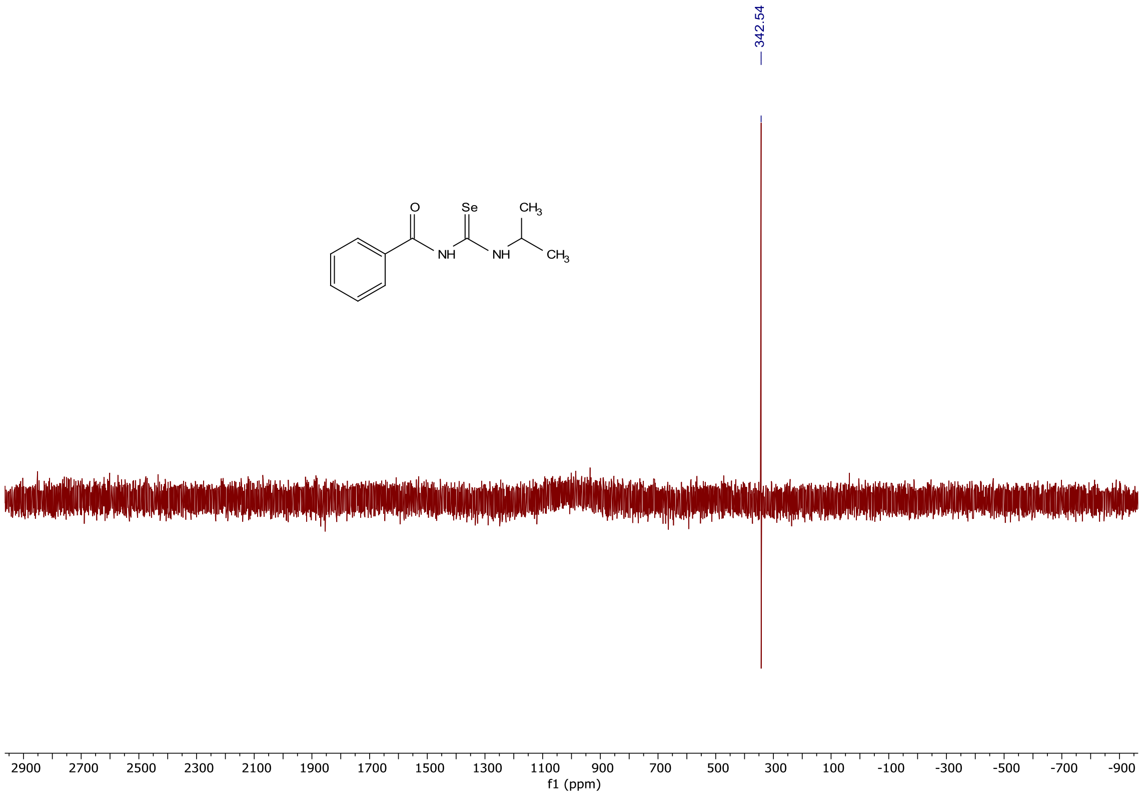

Figure S62. ^{77}Se-NMR spectrum of compound **15**.

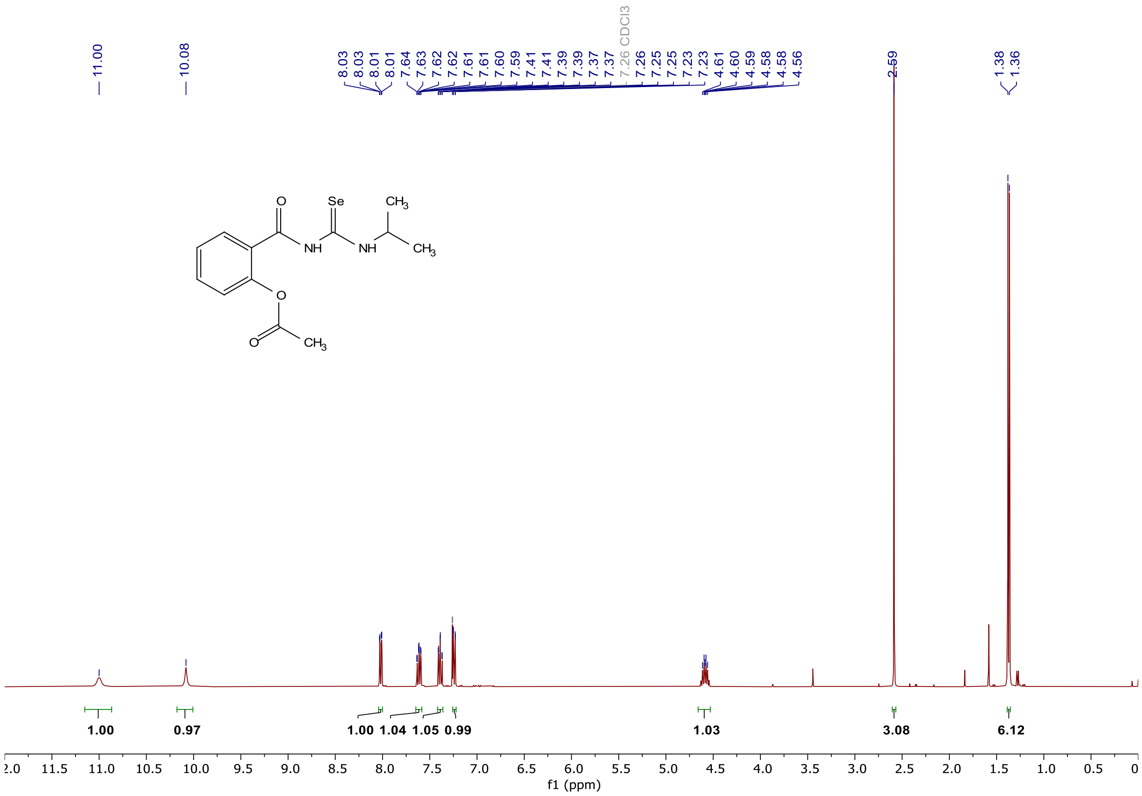

Figure S63. ^{1}H-NMR spectrum of compound **16**.

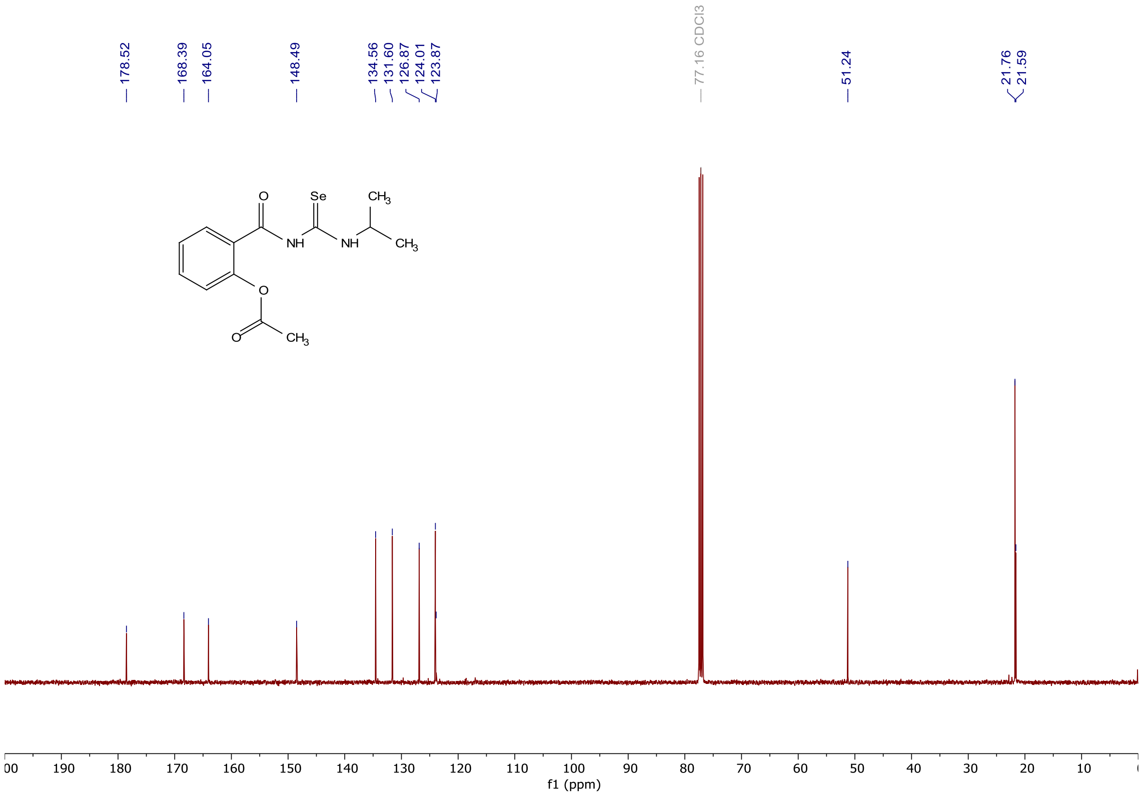

Figure S64. ^{13}C-NMR spectrum of compound **16**.

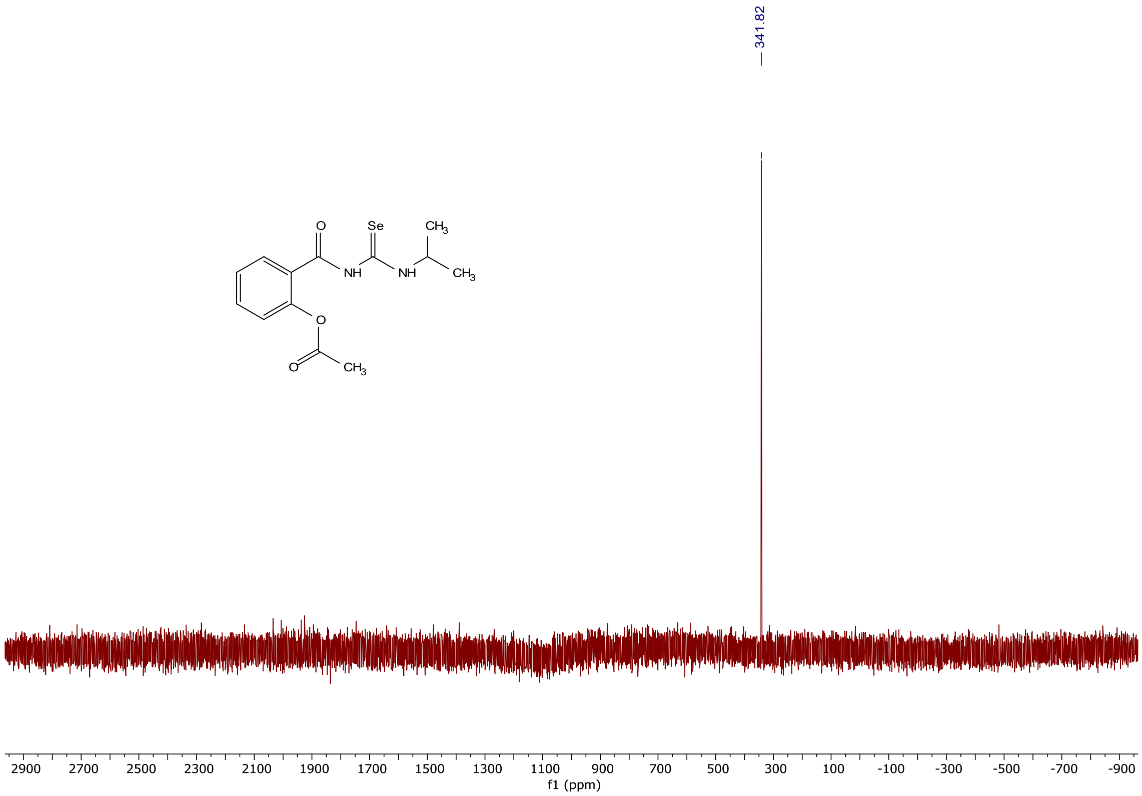

Figure S65. ^{77}Se-NMR spectrum of compound **16**.

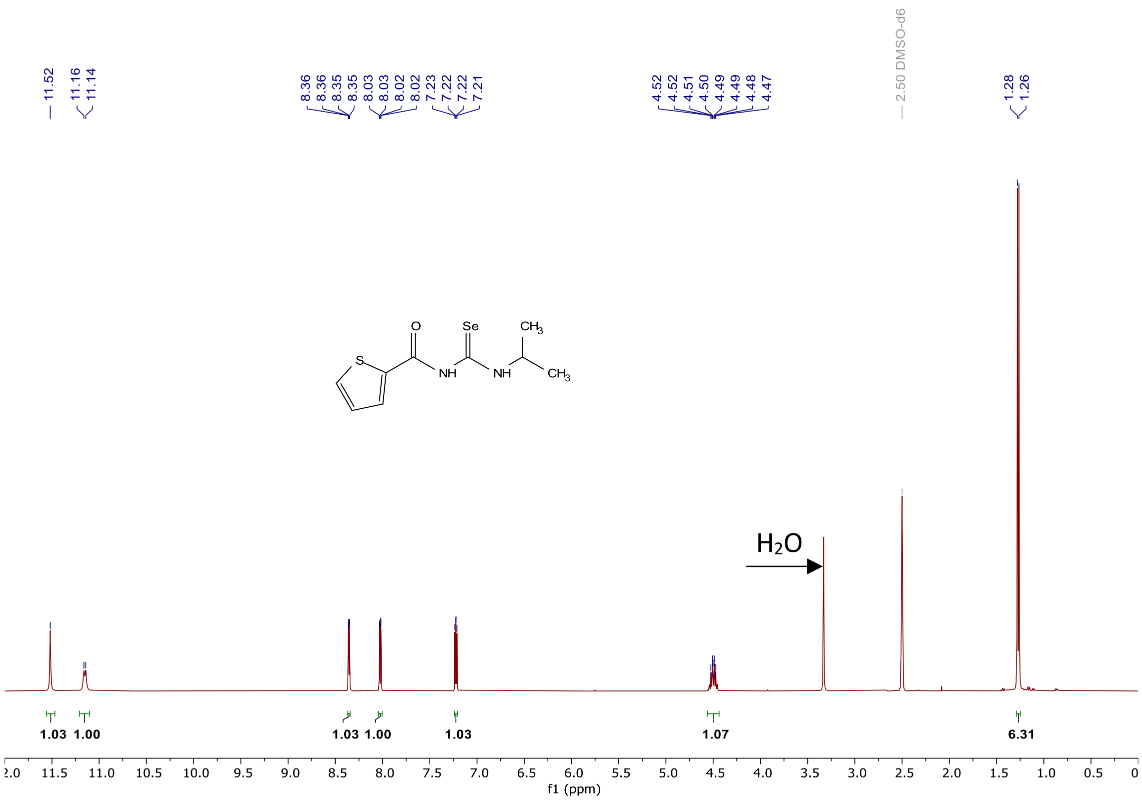

Figure S66. ^{1}H-NMR spectrum of compound **17**.

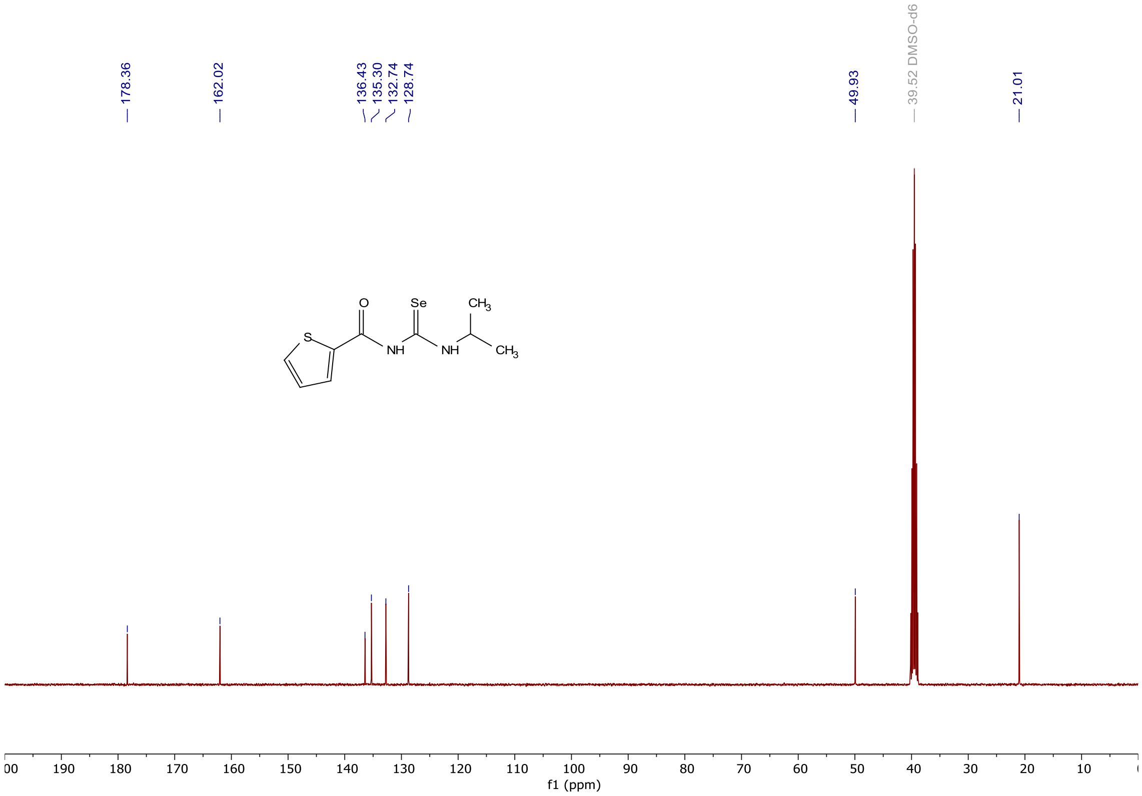

Figure S67. ^{13}C-NMR spectrum of compound **17**.

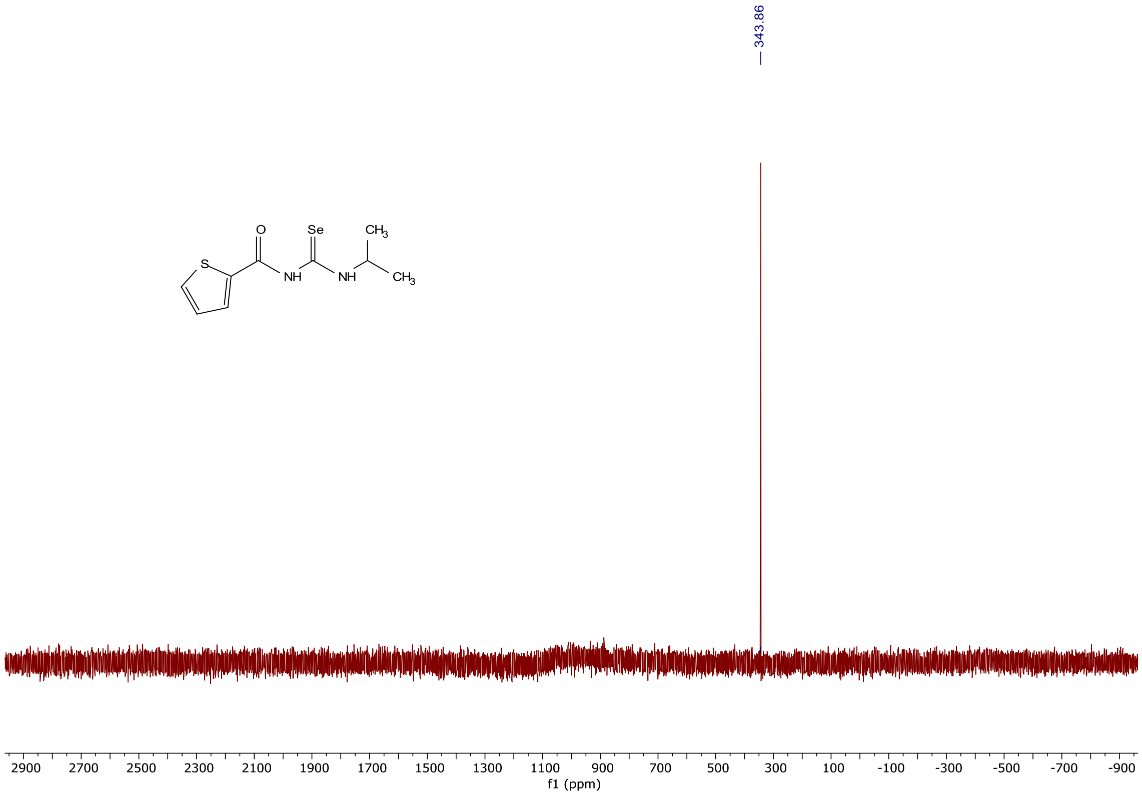

Figure S68. ^{77}Se-NMR spectrum of compound **17**.

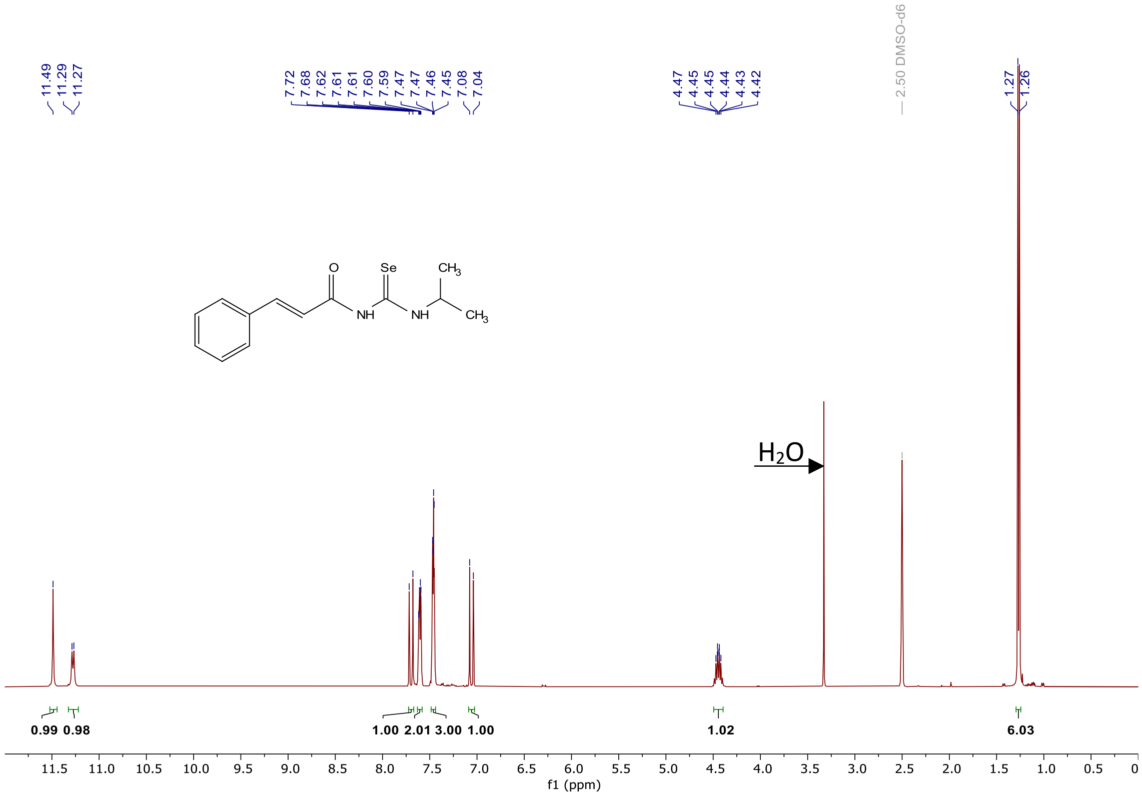

Figure S69. ^{1}H-NMR spectrum of compound **18**.

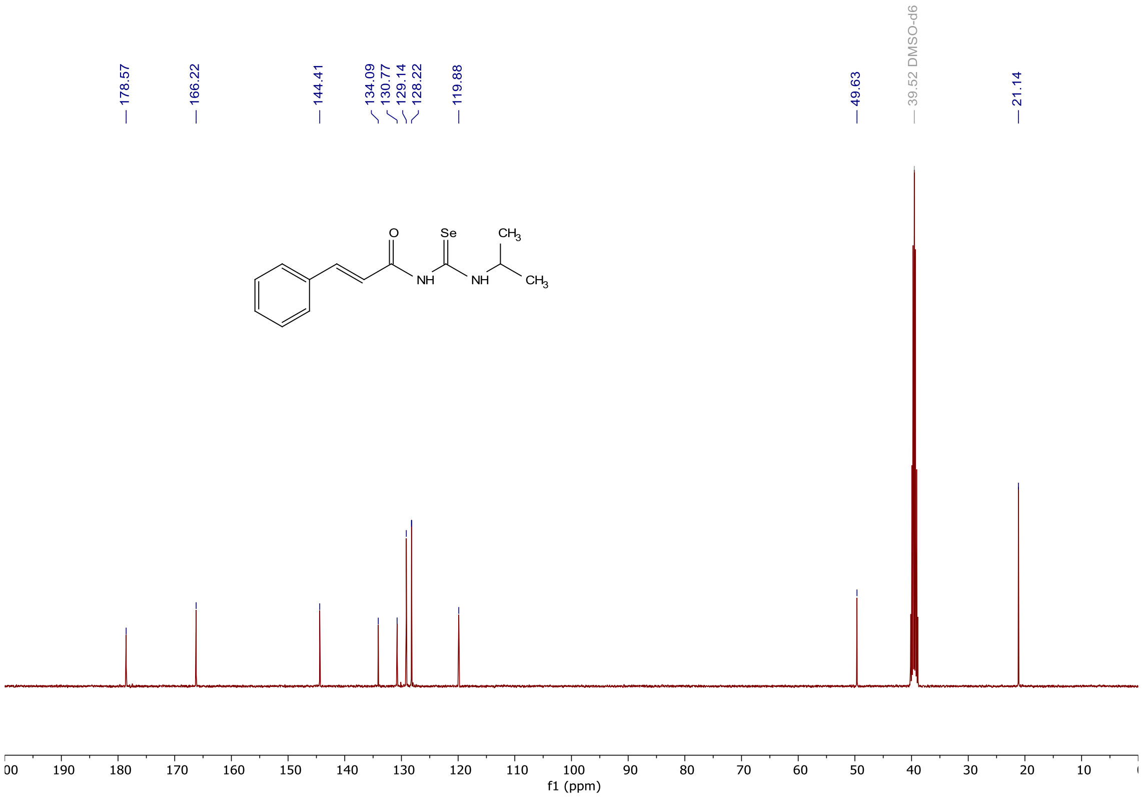

Figure S70. ^{13}C-NMR spectrum of compound **18**.

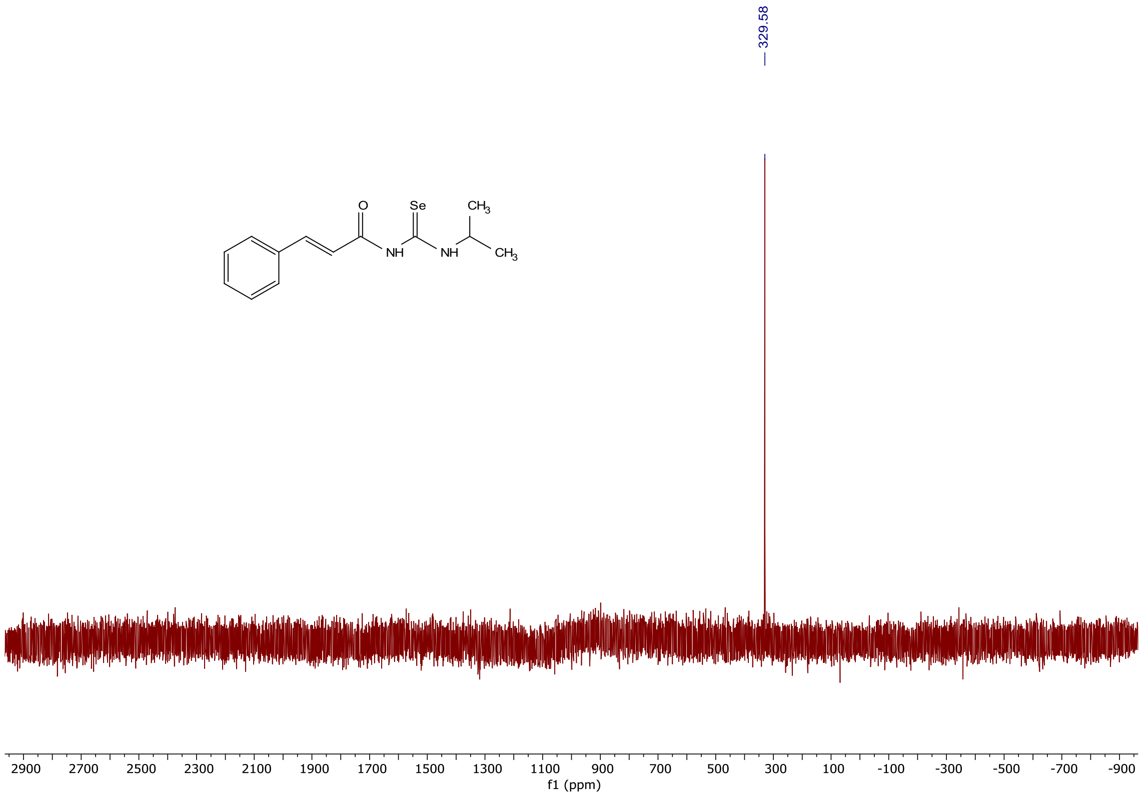

Figure S71. ^{77}Se-NMR spectrum of compound **18**.

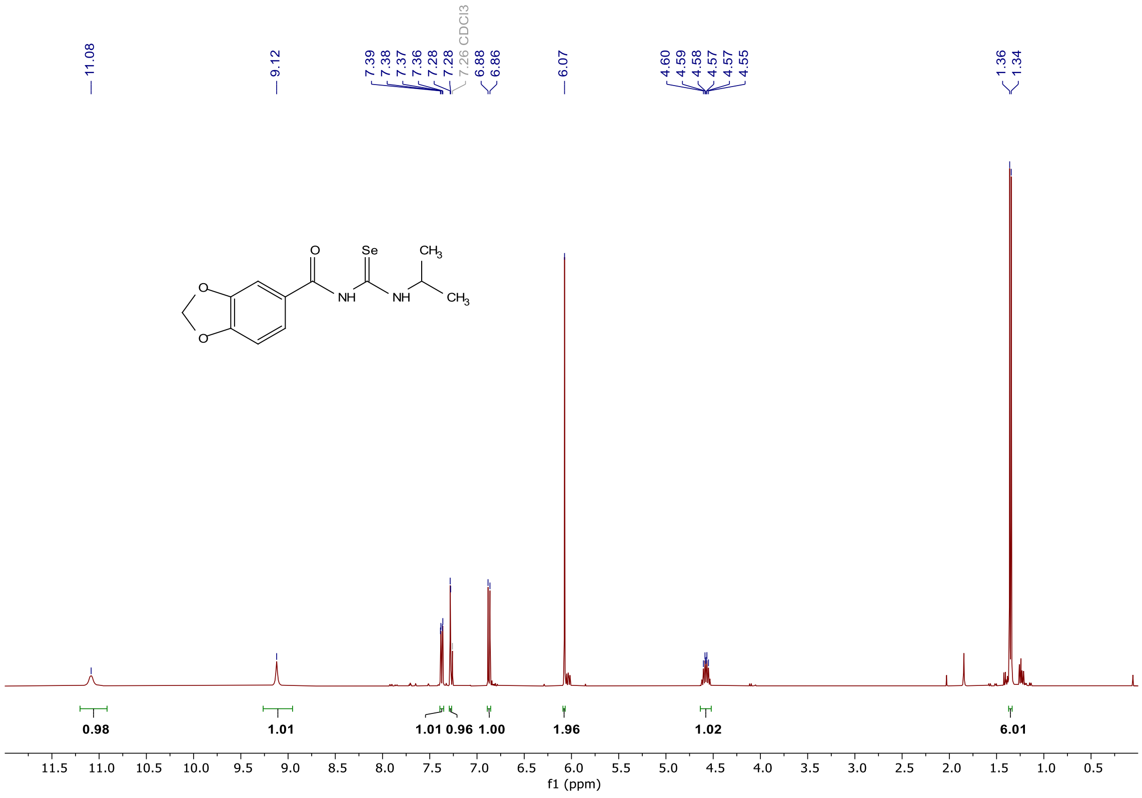

Figure S72. ^{1}H-NMR spectrum of compound **19**.

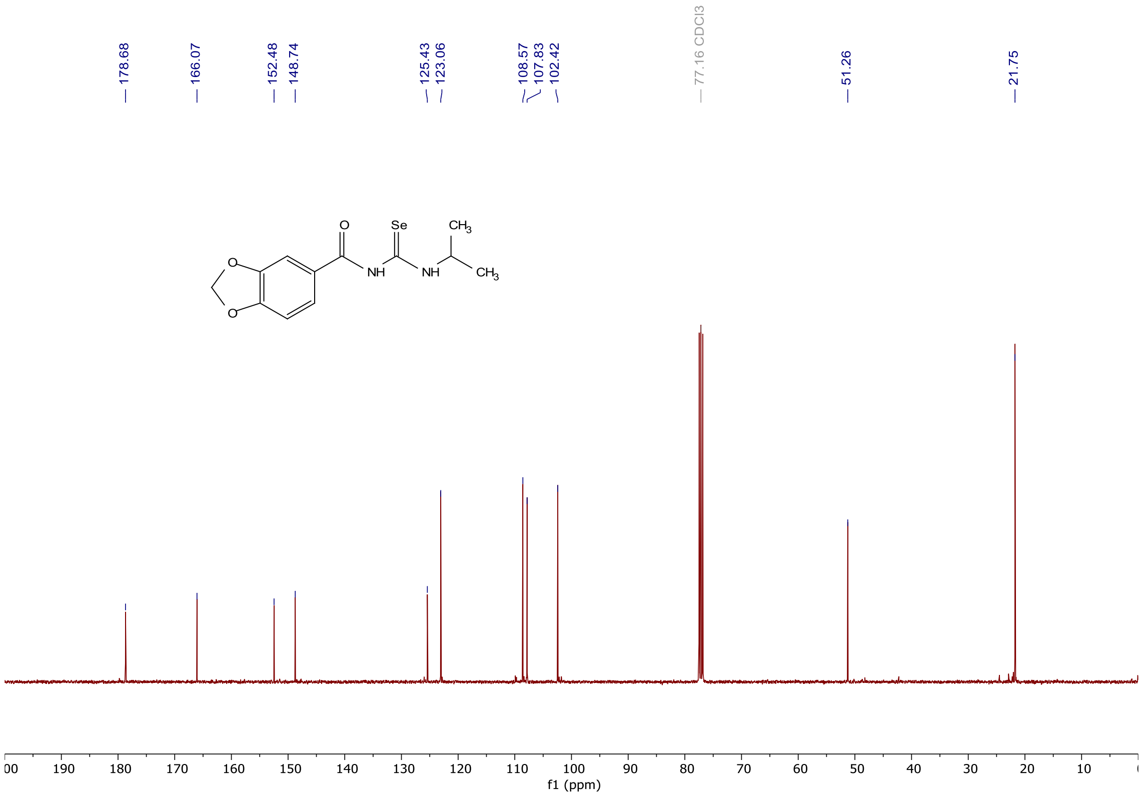

Figure S73. ^{13}C-NMR spectrum of compound **19**.

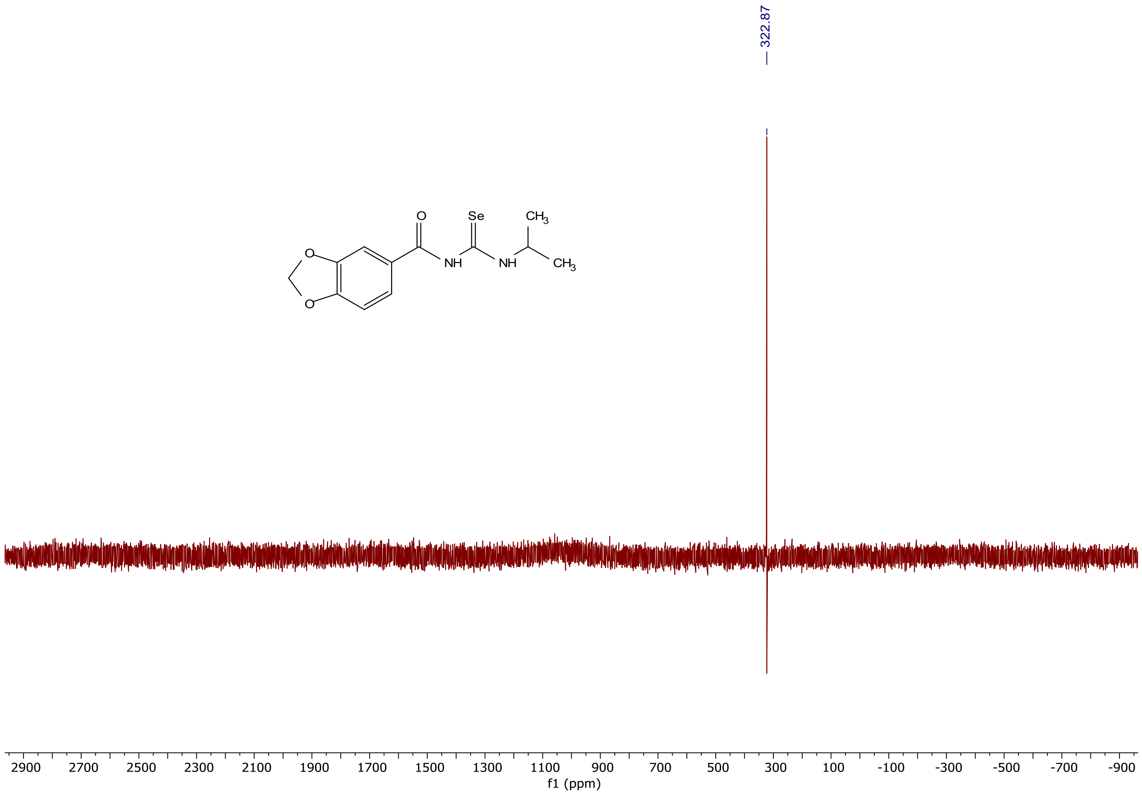

Figure S74. ^{77}Se-NMR spectrum of compound **19**.

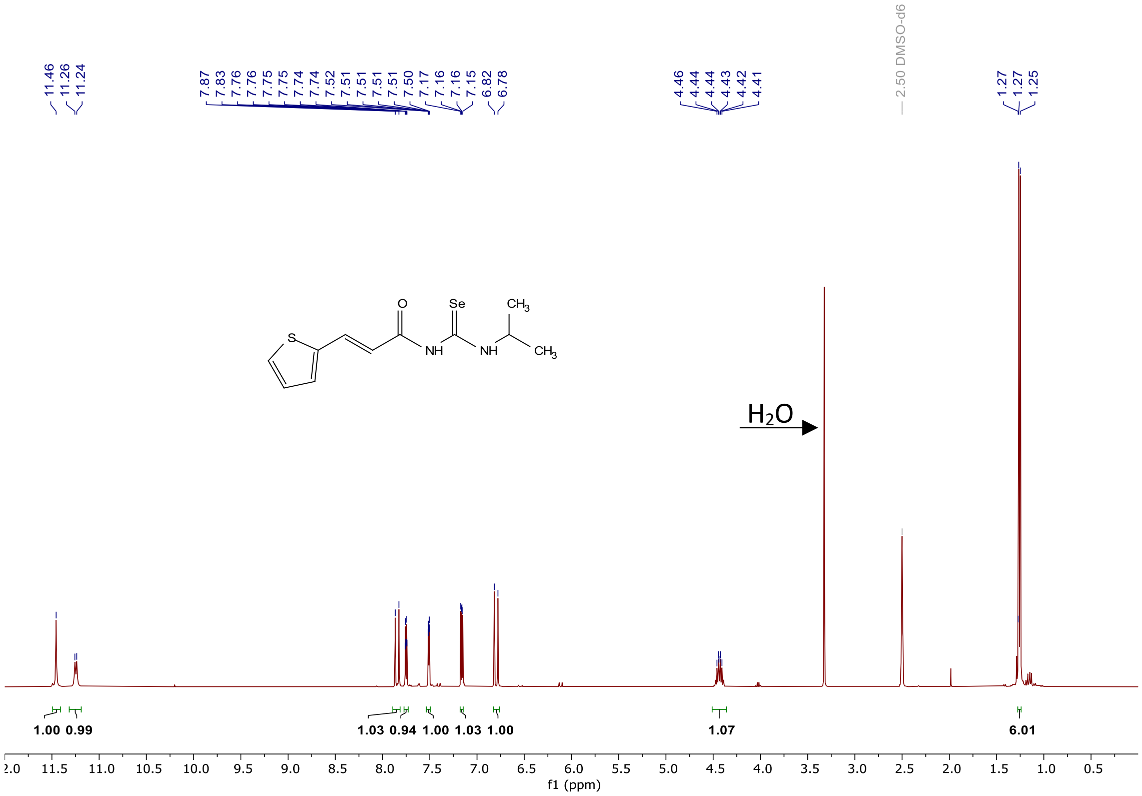

Figure S75. ^{1}H-NMR spectrum of compound **20**.

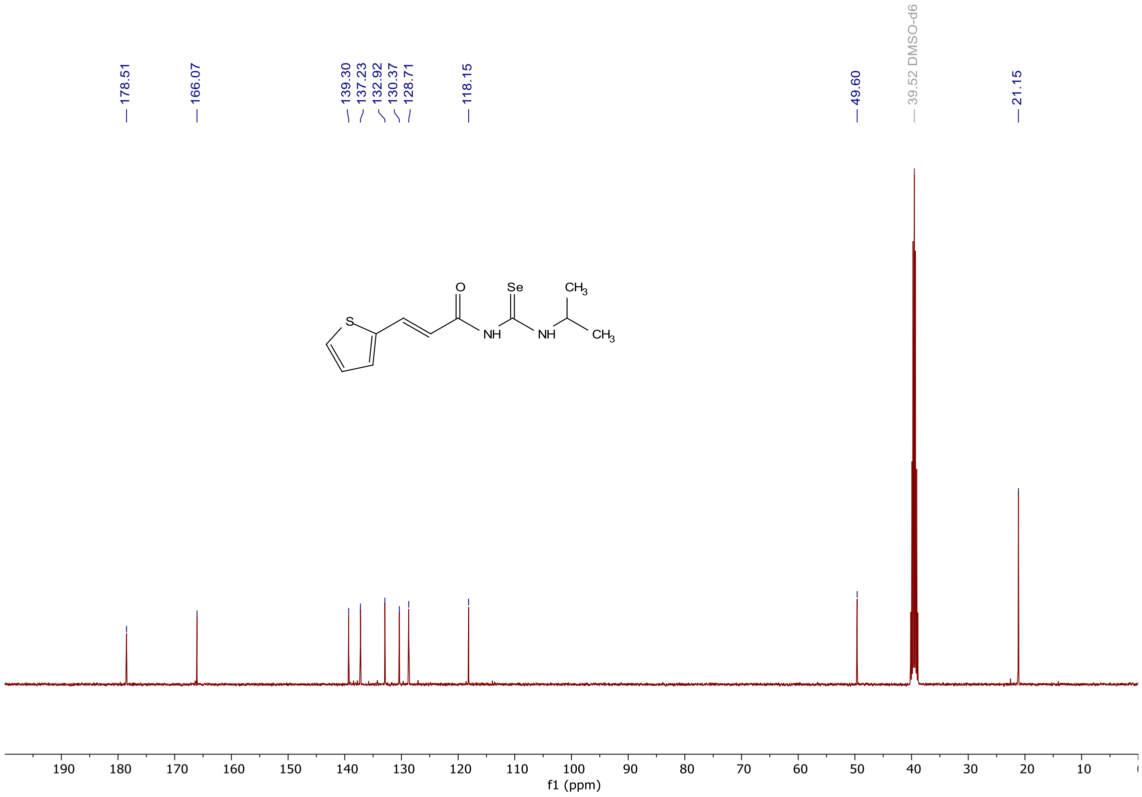

Figure S76. ^{13}C-NMR spectrum of compound **20**.

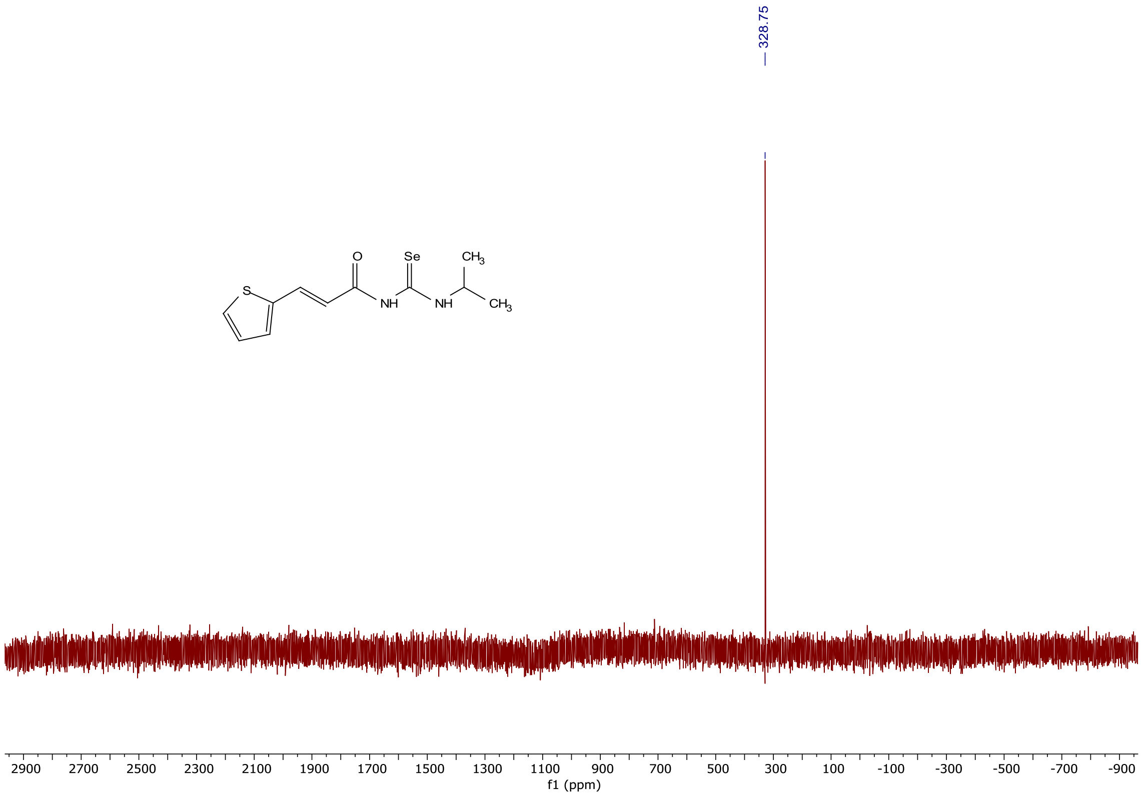

Figure S77. ^{77}Se-NMR spectrum of compound **20**.

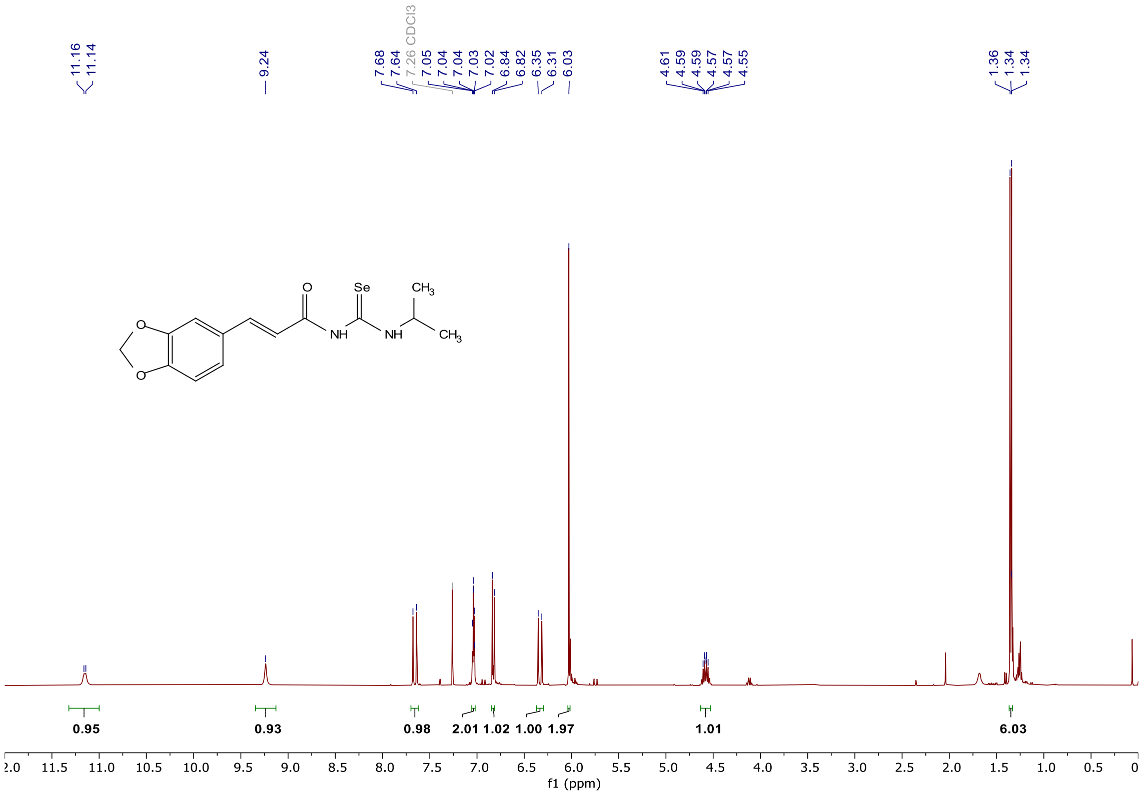

Figure S78. ^{1}H-NMR spectrum of compound **21**.

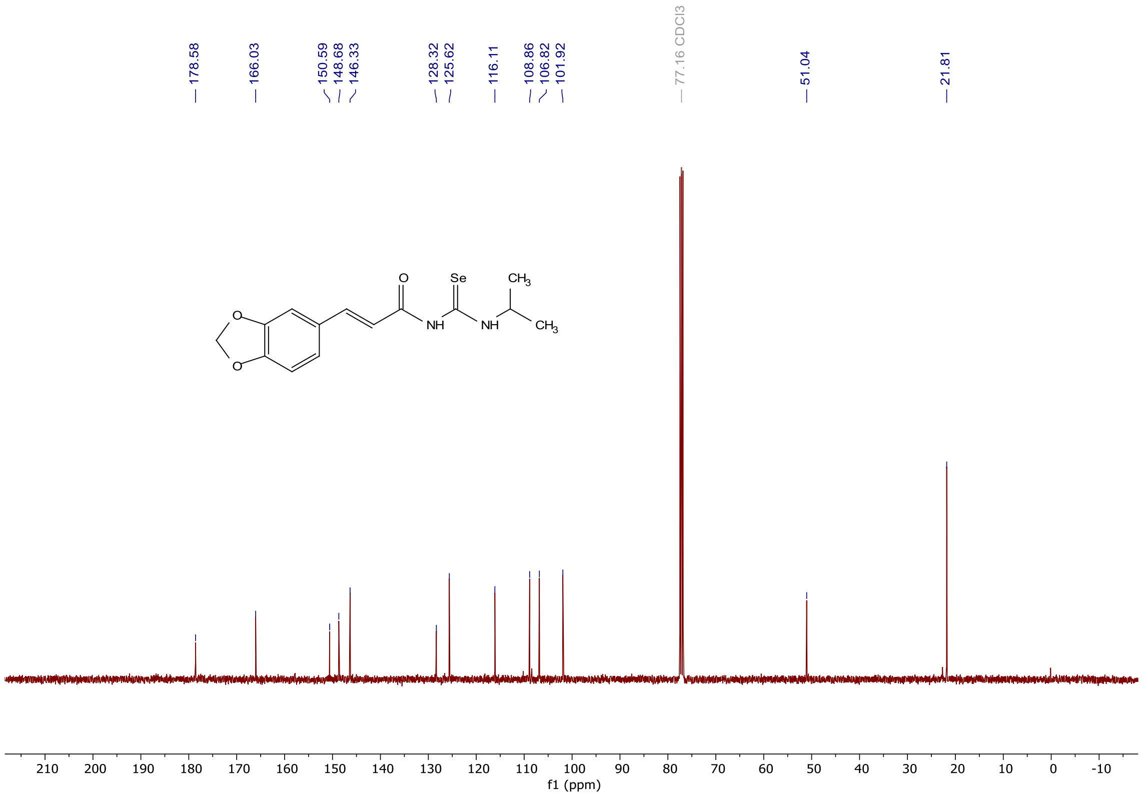

Figure S79. ^{13}C-NMR spectrum of compound **21**.

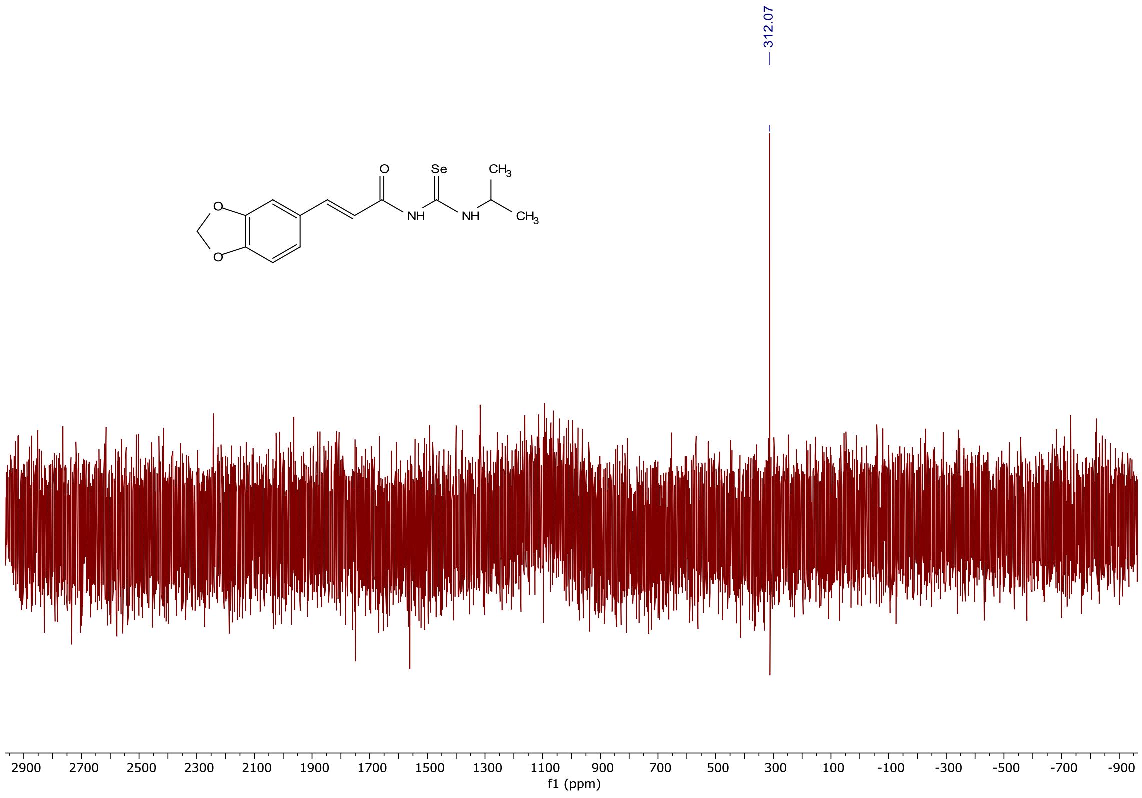

Figure S80. ^{77}Se-NMR spectrum of compound **21**.

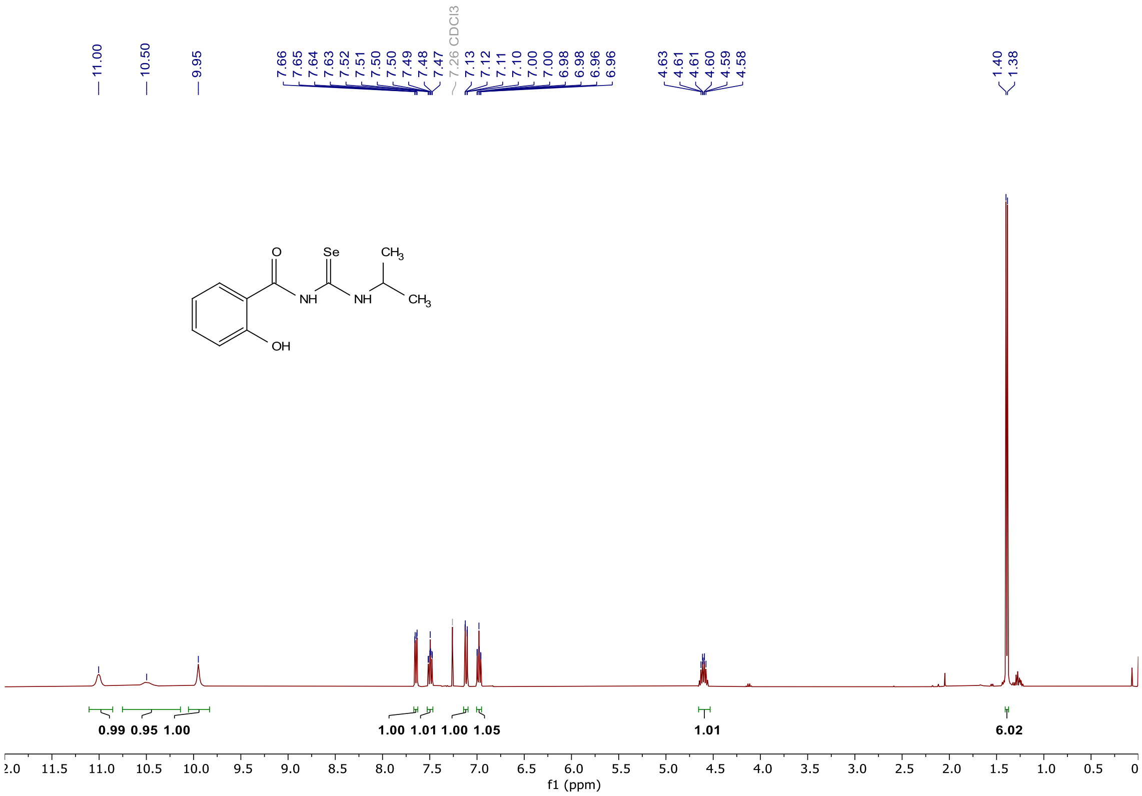

Figure S81. ^{1}H-NMR spectrum of compound **22**.

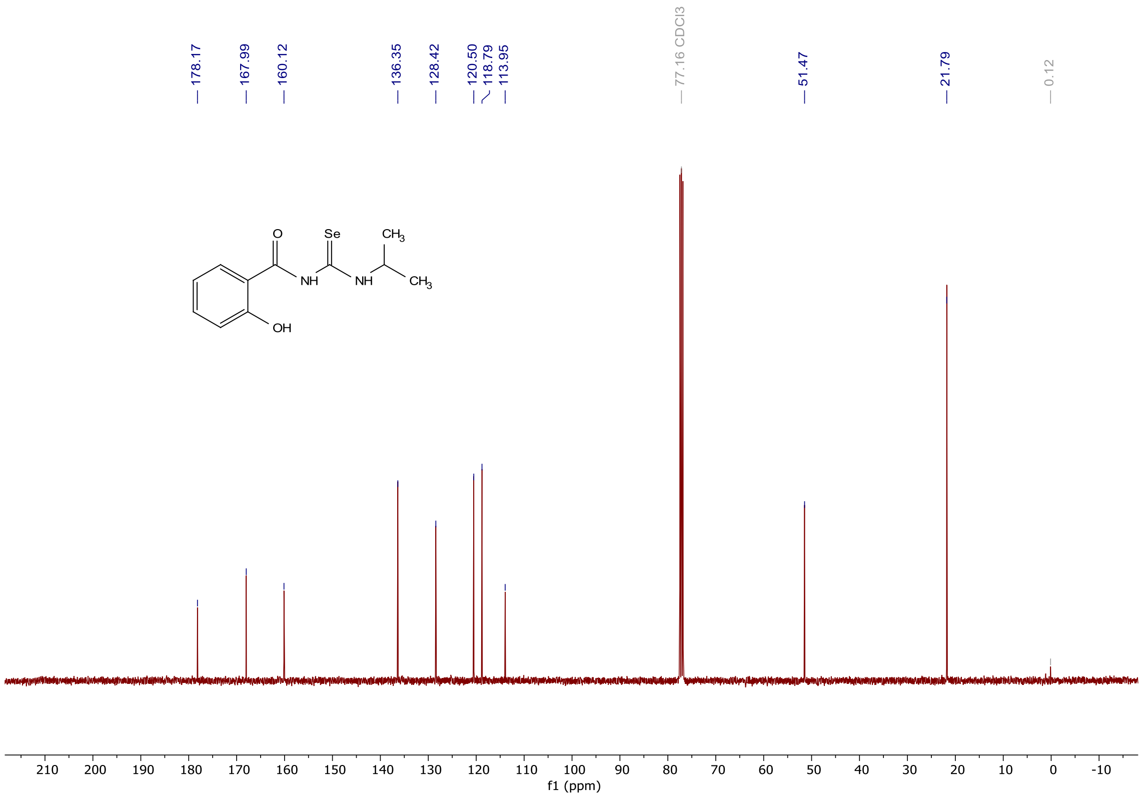

Figure S82. ^{13}C-NMR spectrum of compound **22**.

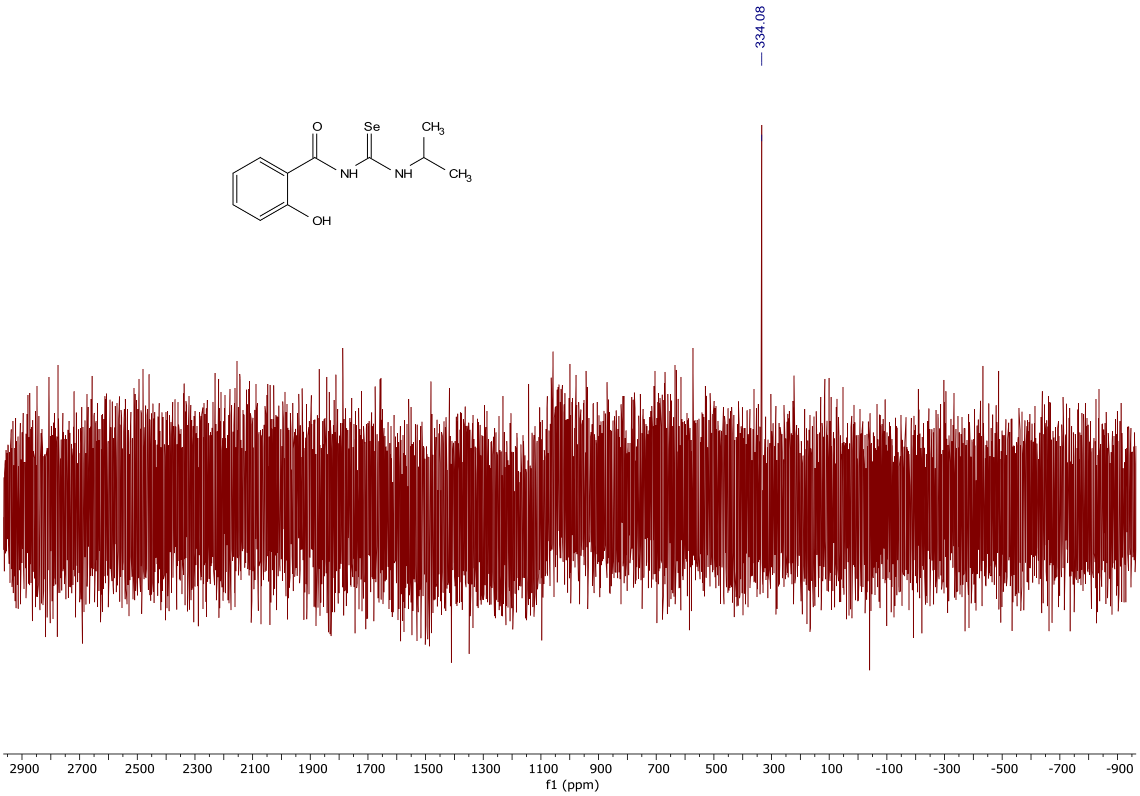

Figure S83. ^{77}Se-NMR spectrum of compound **22**.

Characterization of the compounds synthetized in this work.

(*E*)-2-Acetoxybenzoic (*E*)-*N,N'*-dicyclohexylcarbamimidic selenoanhydride (1). From *O*-acetylsalicyloyl chloride and *N,N´*-dicyclohexylcarbodiimide. Appearance: yellow solid. Melting point: 96-97 ºC. Yield: 21%. ^{1}H-NMR in $CDCl_3$ (400 MHz) δ (ppm): 9.25 (d, *J* = 8.1, 1H, NH), 7.86 (dd, *J* = 7.7, 1.6, 1H, H_{aryl}), 7.40 (td, *J* = 7.8, 1.7, 1H, H_{aryl}), 7.20 (td, *J* = 7.6, 1.1, 1H, H_{aryl}), 7.05 (dd, *J* = 8.2, 1.2, 1H, H_{aryl}), 4.53 (tt, *J* = 12.2, 3.6, 1H, $H_{cyclohexyl}$), 3.97 (dtd, *J* = 10.9, 7.4, 4.0, 1H, $H_{cyclohexyl}$), 2.31 (s, 5H, CH_3 and 2$H_{cyclohexyl}$), 1.93 – 0.79 (m, 18$H_{cyclohexyl}$). ^{13}C-NMR in $CDCl_3$ (100 MHz) δ (ppm): 187.33 (C=N), 171.81 (C=$O_{acetoxy}$), 164.21 (C=O), 148.48 (C_{aryl}), 131.80 (C_{aryl}), 128.63 (C_{aryl}), 127.31 (C_{aryl}), 126.02 (C_{aryl}), 121.92 (C_{aryl}), 62.29 ($C_{cyclohexyl}$), 57.72 ($C_{cyclohexyl}$), 29.58 ($C_{cyclohexyl}$), 26.47 ($C_{cyclohexyl}$), 25.38 ($C_{cyclohexyl}$), 25.18 ($C_{cyclohexyl}$), 24.39 ($C_{cyclohexyl}$), 21.42 (CH_3). ^{77}Se-NMR in $CDCl_3$ (76 MHz) δ (ppm): 462.28. Elemental analysis for $C_{22}H_{30}N_2O_3Se$ calculated/found (percent): C, 58.79/58.83; H, 6.73/6.92; N, 6.23/6.14.

Cinnamic (*E*)-*N,N'*-dicyclohexylcarbamimidic selenoanhydride (2). From cinnamoyl chloride and *N,N´*-dicyclohexylcarbodiimide. Appearance: brown solid. Melting point: 156-158 ºC. Yield: 8%. ^{1}H-NMR in DMSO-d_6 (400 MHz) δ (ppm): 8.41 (d, *J* = 7.9, 1H, NH), 7.59 – 7.48 (m, 3H, H_2, H_{aryl} y H_{alkene}), 7.47 – 7.36 (m, 3H, H_{aryl}), 6.68 (d, *J* = 15.5, 1H, H_{alkene}), 4.09 (ddt, *J* = 12.0, 7.3, 3.5, 1H, $H_{cyclohexyl}$), 3.64 – 3.49 (m, 1H, $H_{cyclohexyl}$), 1.86 – 1.42 (m, 12H, $H_{cyclohexyl}$), 1.33 – 0.97 (m, 8H, $H_{cyclohexyl}$). ^{13}C-NMR in DMSO-d_6 (100 MHz) δ (ppm): 162.74 (C=O), 153.34 (C=N), 140.93 (C_{alkene}), 134.65 (C_{aryl}), 129.83 (C_{aryl}), 129.04 (C_{aryl}), 127.65(C_{aryl}), 119.65 (C_{alkene}), 52.83 (C_{alkene}), 49.60 (C_{alkene}), 31.85 ($C_{cyclohexyl}$), 30.43 ($C_{cyclohexyl}$), 25.45 ($C_{cyclohexyl}$), 25.15 ($C_{cyclohexyl}$), 25.05

($C_{cyclohexyl}$), 24.38 ($C_{cyclohexyl}$). ^{77}Se-NMR in DMSO-d_6 (76 MHz) δ (ppm): peak not found. Elemental analysis for $C_{22}H_{30}N_2OSe$ calculated/found (percent): C, 63.30/63.59; H, 7.24/7.19; N, 6.71/6.74.

Benzoic *N,N'*-diisopropylcarbamimidic selenoanhydride (3). From benzoyl chloride and *N,N´*-diisopropylcarbodiimide. Appearance: yellow solid. Melting point: 163-165 ºC. Yield: 58%. ^{1}H-NMR in DMSO-d_6 (400 MHz) δ (ppm): 10.35 (d, *J* = 7.9, 1H, NH), 7.63 – 7.60 (m, 2H, H_{aryl}) , 7.46 – 7.40 (m, 1H, H_{aryl}), 7.39 – 7.33 (m, 2H, H_{aryl}), 4.87 (p, *J* = 6.8, 1H, $H_{isopropyl}$), 4.09 (dt, *J* = 8.0, 6.5, 1H, $H_{isopropyl}$), 1.43 (d, *J* = 6.9, 6H, $H_{isopropyl}$), 0.70 (d, *J* = 6.6, 6H, $H_{isopropyl}$). ^{13}C-NMR in DMSO-d_6 (100 MHz) δ (ppm): 185.77 (C=N), 167.97 (C=O), 137.01 (C_{aryl}), 130.30 (C_{aryl}), 127.72 (C_{aryl}), 127.08 (s, 2C, C_{aryl}), 51.33 ($C_{isopropyl}$), 50.19 ($C_{isopropyl}$), 19.93 ($C_{isopropyl}$), 19.31 ($C_{isoporpyl}$). ^{77}Se-NMR in DMSO-d_6 (76 MHz) δ (ppm): 474.01. Elemental analysis for $C_{14}H_{20}N_2OSe$ calculated/found (percent): C, 54.02/54.37; H, 6.48/6.23; N, 9.00/8.98.

(*E*)-2-Acetoxybenzoic (*E*)-*N,N'*-diisopropylcarbamimidic selenoanhydride (4). From *O*-acetylsalicyloyl chloride and *N,N´*-diisopropylcarbodiimide. Appearance: yellow solid. Melting point: 79-81 ºC. Yield: 40%. ^{1}H-NMR in DMSO-d_6 (400 MHz) δ (ppm): 10.19 (d, *J* = 8.0, 1H, NH), 7.57 (d, *J* = 7.5, 1H, H_{aryl}), 7.44 (td, *J* = 10.0, 8.0, 2.0, 1H, H_{aryl}), 7.28 – 7.18 (m, 2H, H_{aryl}), 4.77 – 4.65 (m, 1H, $H_{isopropyl}$), 4.10 (h, *J* = 6.7, 1H, $H_{isopropyl}$), 2.24 (s, 3H, CH_3), 1.42 (dd, *J* = 6.8, 2.3, 6H, $H_{isopropyl}$), 0.83 (dd, *J* = 6.7, 2.3, 6H, $H_{isopropyl}$). ^{13}C-NMR in DMSO-d_6 (100 MHz) δ (ppm): 184.80 (C=N), 169.00 (C=$O_{acetoxy}$), 163.61 (C=O), 147.17 (C_{aryl}), 130.54 (C_{aryl}), 129.20 (C_{aryl}), 128.00 (C_{aryl}), 125.03 (C_{aryl}), 122.87 (C_{aryl}), 50.72 ($C_{isopropyl}$), 50.24 ($C_{isopropyl}$), 21.19 (CH_3), 19.74 ($C_{isopropyl}$), 19.36 ($C_{isopropyl}$). ^{77}Se-NMR in $CDCl_3$ (76 MHz) δ (ppm): 458.28. Elemental analysis for $C_{16}H_{22}N_2O_3Se$ calculated/found (percent): C, 52.03/51.95; H, 6.00/6.32; N, 7.58/7.48.

(*E*)-(*E*)-*N,N'*-Diisopropylcarbamimidic 2-(thiophen-2-yl)acetic selenoanhydride (5). From thiophene-2-acetyl chloride and *N,N´*-diisopropylcarbodiimide. Appearance: yellow solid. Melting point: 108-110 ºC. Yield: 15%. ^{1}H-NMR in DMSO-d_6 (400 MHz) δ (ppm): 11.13 (d, *J* = 7.9, 1H, NH), 7.37 (dd, *J* = 5.1, 1.3, 1H, H_{aryl}), 6.94 (dd, *J* = 5.1, 3.5, 1H, H_{aryl}), 6.90 (dd, *J* = 3.5, 1.2, 1H, H_{arryl}), 4.45 (m, 2H, $H_{isopropyl}$), 3.87 (d, *J* = 0.9, 2H, CH_2), 1.27 (dd, *J* = 10.4, 6.7, 12H, $H_{isopropyl}$). ^{13}C-NMR in DMSO-d_6 (100 MHz) δ (ppm): 184.92 (C=N), 164.95 (C=O), 136.43 (C_{aryl}), 126.69 (C_{aryl}), 126.29 (C_{aryl}), 125.34 (C_{aryl}), 50.84 ($C_{isopropyl}$), 48.01 ($C_{isopropyl}$), 34.97 (CH_2), 19.80 ($C_{isopropyl}$), 19.70 ($C_{isopropyl}$). ^{77}Se-NMR in DMSO-d_6 (76 MHz) δ (ppm): 582.90. Elemental analysis for $C_{13}H_{20}N_2OSSe$ calculated/found (percent): C, 47.12/47.37; H, 6.08/6.25; N, 8.45/8.43.

(*E*)-(*E*)-*N,N'*-Diisopropylcarbamimidic thiophene-2-carboxylic selenoanhydride (6). From 2-thiophenecarbonyl chloride and *N,N´*-diisopropylcarbodiimide. Appearance: yellow solid. Melting point: 120-122 ºC. Yield: 46%. ^{1}H-NMR in $CDCl_3$ (400 MHz) δ (ppm): 7.80 (dd, *J* = 3.9, 1.2, 1H, H_{aryl}), 7.72 (d, *J* = 7.1, 1H, NH), 7.48 (dd, *J* = 5.0, 1.2, 1H, H_{aryl}), 7.00 (dd, *J* = 5.0, 3.8, 1H, H_{aryl}), 5.05 (p, *J* = 6.8, 1H, $H_{isopropyl}$), 4.56 – 4.46 (m, 1H, $H_{isopropyl}$), 1.45 (d, *J* = 6.8, 6H, $H_{isopropyl}$), 1.07 (d, *J* = 6.6, 6H, $H_{isopropyl}$). ^{13}C-NMR in $CDCl_3$ (100 MHz) δ (ppm): 187.72 (C=N), 162.59 (C=O), 138.53 (C_{aryl}), 131.76 (C_{aryl}), 131.59 (C_{aryl}), 127.61 (C_{aryl}), 54.22($C_{isopropyl}$), 51.02 ($C_{isopropyl}$), 20.61 ($C_{isopropyl}$), 20.42 ($C_{isopropyl}$). ^{77}Se-NMR in $CDCl_3$ (76 MHz) δ (ppm): 529.29. Elemental analysis for $C_{12}H_{18}N_2OSSe$ calculated/found (percent): C, 45.42/45.22; H, 5.72/5.81; N, 8.83/8.90.

(*E*)-Benzo[*d*][1,3]dioxole-5-carboxylic (*E*)-*N,N'*-diisopropylcarbamimidic selenoanhydride (7). From piperonylic acid and *N,N´*-diisopropylcarbodiimide. Appearance: yellow solid. Melting point: 138-140 ºC. Yield: 14%. ^{1}H-NMR in DMSO-d_6 (400 MHz) δ (ppm): 10.29 (d, *J* = 8.1, 1H, NH), 7.20 (dd, *J* = 8.1, 1.7, 1H, H_{aryl}), 7.11 (d, *J* = 1.7, 1H, H_{aryl}), 6.91 (d, *J* = 8.1, 1H, H_{aryl}), 6.05 (s, 2H, H_{aryl}), 4.88 (p, *J* = 6.8, 1H, $H_{isopropyl}$), 4.26 – 4.12 (m, 1H, $H_{isopropyl}$), 1.40 (d, *J* = 6.8, 6H, $H_{isopropyl}$), 0.81 (d, *J* = 6.7, 6H, $H_{isopropyl}$). ^{13}C-NMR in DMSO-d_6 (100 MHz) δ (ppm): 186.07 (C=N), 167.58 (C=O), 149.13 (C_{aryl}), 146.65 (C_{aryl}), 130.82 (C_{aryl}), 122.10 (C_{aryl}), 107.91 (C_{aryl}), 107.50 (C_{aryl}), 101.53 (CH_2), 51.85 ($C_{isopropyl}$), 50.32 ($C_{isopropyl}$), 19.97 ($C_{isopropyl}$), 19.57 ($C_{isopropyl}$). ^{77}Se-NMR in DMSO-d_6 (76 MHz) δ (ppm): 462.62. Elemental analysis for $C_{15}H_{20}N_2O_3Se$ calculated/found (percent): C, 50.71/51.02; H, 5.67/5.59; N, 7.88/8.00.

***N*-(Cyclohexylcarbamoselenoyl) benzamide (8).** From benzoyl chloride and cyclohexylamine. Appearance: Yellow solid. Melting point: 71-73 ºC. Yield: 11%. ^{1}H-NMR in DMSO-d_6 (400 MHz) δ (ppm): 11.51 (s, 1H, NH), 11.43 (d, *J* = 8.0, 1H, NH), 7.93 – 7.89 (m, 2H, H_{aryl}), 7.67 – 7.61 (m, 1H, H_{aryl}), 7.51 (t, *J* = 7.8, 2H, H_{aryl}), 4.34 – 4.23 (m, 1H, $H_{cyclohexyl}$), 2.03 – 1.96 (m, 2H, $H_{cyclohexyl}$), 1.66 (dt, *J* = 12.8, 4.5, 2H, $H_{cyclohexyl}$), 1.60 – 1.52 (m, 1H, $H_{cyclohexyl}$), 1.50 – 1.25 (m, 5H, $H_{cyclohexyl}$). ^{13}C-NMR in DMSO-d_6 (100 MHz) δ (ppm): 178.85 (C=Se), 168.49 (C=O), 133.11 (C_{aryl}), 131.94 (C_{aryl}), 128.65 (C_{aryl}), 128.39 (C_{aryl}), 56.10 (C_{aryl}), 30.63($C_{cyclohexyl}$), 24.87 ($C_{cyclohexyl}$), 23.96 ($C_{cyclohexyl}$). ^{77}Se-NMR in DMSO-d_6 (76 MHz) δ (ppm): 339.64. Elemental analysis for $C_{14}H_{18}N_2OSe$ calculated/found (percent): C, 54.37/54.56; H, 5.87/5.64; N, 9.06/9.13.

2-((Cyclohexylcarbamoselenoyl)carbamoyl)phenyl acetate (9). From *O*-acetylsalicyloyl chloride and cyclohexylamine. Appearance: brown solid. Melting point: 79-81 ºC. Yield: 10%. ^{1}H-NMR in DMSO-d_6 (400 MHz) δ (ppm): 11.52 (s, 1H, NH), 11.13 (d, *J* = 8.0, 1H, NH), 7.70 (dd, *J* = 7.7, 1.7, 1H, H_{aryl}), 7.63 (ddd, *J* = 8.2, 7.5, 1.7, 1H, H_{aryl}), 7.38 (td, *J* = 7.6, 1.1, 1H, H_{aryl}), 7.28 (dd, *J* = 8.1, 1.1, 1H, H_{aryl}), 4.25 (m, 1H, $H_{cyclohexyl}$), 2.32 (s, 3H, CH_3), 1.99 (s, 2H, $H_{cyclohehyl}$), 1.72 – 1.21 (m, 8H, $H_{cyclohexyl}$). ^{13}C-NMR in DMSO-d_6 (100 MHz) δ (ppm): 178.39 (C=Se), 168.75 (C=$O_{acetoxy}$), 166.33 (C=O), 148.10 (C_{aryl}), 133.25 (C_{aryl}), 130.10 (C_{aryl}), 126.28 (C_{aryl}), 125.90 (C_{aryl}), 123.17 (C_{aryl}), 56.18 ($C_{cyclohexyl}$), 30.59 ($C_{cyclohexyl}$), 24.82 ($C_{cyclohexyl}$), 23.95 ($C_{ciclohexilo}$), 20.82 (CH_3). ^{77}Se-NMR in DMSO-d_6 (76 MHz) δ (ppm): 343.24. Elemental analysis for $C_{16}H_{20}N_2O_3Se$ calculated/found (percent): C, 52.32/52.01; H, 5.49/5.58; N, 7.63/7.70.

***N*-(Cyclohexylcarbamoselenoyl) tiophene-2-carboxamide (10).** From 2-thiophenecarbonyl chloride and cyclohexylamine. Appearance: yellow solid. Melting point: 64-66 ºC. Yield: 12%. ^{1}H-NMR in DMSO-d_6 (400 MHz) δ (ppm): 11.57 (s, 1H, NH), 11.31 (d, *J* = 8.0, 1H, NH), 8.37 (dd, *J* = 4.0, 1.1, 1H, H_{aryl}), 8.04 (dd, *J* = 5.0, 1.1, 1H, H_{aryl}), 7.23 (dd, *J* = 5.0, 3.9, 1H, H_{aryl}), 4.28 (tq, *J* = 9.3, 5.4, 4.7, 1H, $H_{cyclohexyl}$), 2.00 – 1.94 (m, 2H, $H_{cyclohexyl}$), 1.64 (dt, *J* = 8.8, 4.2, 2H, $H_{cyclohexyl}$), 1.58 – 1.51 (m, 1H, $H_{cyclohexyl}$), 1.47 – 1.20 (m, 5H, $H_{cyclohexyl}$). ^{13}C-NMR in DMSO-d_6 (100 MHz) δ (ppm): 178.41 (C=Se), 162.25 (C=O), 136.39 (C_{aryl}), 135.36 (C_{aryl}), 132.83 (C_{aryl}), 128.75 (C_{aryl}), 56.09 ($C_{cyclohexyl}$), 30.59 ($C_{cyclohexyl}$), 24.87 ($C_{cyclohexyl}$), 23.92 ($C_{cyclohexyl}$). ^{77}Se-NMR in $CDCl_3$ (76 MHz) δ (ppm): 327.75. Elemental analysis for $C_{12}H_{16}N_2OSSe$ calculated/found (percent): C, 45.71/45.91; H, 5.12/5.14; N, 8.88/8.73.

***N*-(Cyclohexylcarbamoselenoyl)cinnamamide (11).** From cinnamoyl chloride and cyclohexylamine. Appearance: yellow solid. Melting point: 140-142 ºC. Yield: 38%. ^{1}H-NMR in DMSO-d_6 (400 MHz) δ (ppm): 11.52 (s, 1H, NH), 11.45 (d, *J* = 8.1, 1H, NH), 7.72 (d, *J* = 15.7, 1H, H_{alkene}), 7.60 (m, 2H, H_{aryl}), 7.48 – 7.44 (m, 3H, H_{aryl}), 7.06 (d, *J* = 15.7, 1H, H_{alkene}), 4.23 (dt, *J* = 8.6, 4.3, 1H, $H_{cyclohexyl}$), 1.96 (dt, *J* = 8.7, 4.4, 2H, $H_{cyclohexyl}$), 1.67 – 1.21 (m, 8H, $H_{cyclohexyl}$). ^{13}C-NMR in DMSO-d_6 (100 MHz) δ (ppm): 178.56 (C=Se), 166.41 (C=O), 144.52 (C_{alkene}), 134.10 (C_{aryl}), 130.77 (C_{aryl}), 129.13 (C_{aryl}), 128.23 (C_{aryl}), 119.82 (C_{alkene}), 55.67 ($C_{cyclohexyl}$), 30.67 ($C_{cyclohexyl}$), 24.85 ($C_{cyclohexyl}$), 23.80 ($C_{cyclohexyl}$). ^{77}Se-NMR in DMSO-d_6 (76 MHz) δ (ppm): 326.55. Elemental analysis for $C_{16}H_{20}N_2OSe$ calculated/found (percent): C, 57.31/57.28; H, 6.01/5.94; N, 8.35/8.53.

***N*-(Cyclohexylcarbamoselenoyl)benzo[d][1,3]dioxole-5-carboxamide (12).** From piperonylic acid and cyclohexylamine. Appearance: brown solid. Melting point: 117-119 ºC. Yield: 51%. ^{1}H-NMR in $CDCl_3$ (400 MHz) δ (ppm): 11.19 (d, 1H, NH), 9.11 (s, 1H, NH), 7.38 (dd, *J* = 8.2, 1.9, 1H, H_{aryl}), 7.28 (d, *J* = 1.9, 1H, H_{aryl}), 6.87 (d, *J* = 8.2, 1H, H_{aryl}), 6.07 (s, 2H, CH_2), 4.37 – 4.27 (m, 1H, $H_{cyclohexyl}$), 2.14 – 2.06 (m, 2H, $H_{cyclohexyl}$), 1.76 – 1.26 (m, 8H, $H_{cyclohexyl}$). ^{13}C-NMR in $CDCl_3$ (100 MHz) δ (ppm): 178.39, (C=Se) 166.07 (C=O), 152.47 (C_{aryl}), 148.74 (C_{aryl}) 125.45 (C_{aryl}), 123.06 (C_{aryl}), 108.58 (C_{aryl}), 107.83 (C_{aryl}), 102.42 (CH_2), 57.55 ($C_{cyclohexyl}$), 31.58 ($C_{cyclohexyl}$), 25.44 ($C_{cyclohexyl}$), 24.37 ($C_{cyclohexyl}$). ^{77}Se-NMR in $CDCl_3$ (76 MHz) δ (ppm): 317.31. Elemental analysis for $C_{15}H_{18}N_2O_3Se$ calculated/found (percent): C, 51.00/50.89; H, 5.14/5.03; N, 7.93/8.06.

(*E*)-*N*-(Cyclohexylcarbamoselenoyl)-3-(thiophen-2-yl)acrylamide (13). From 3-(2-thienyl)acrylic acid and cyclohexylamine. Appearance: brown solid. Melting point: 113-115 ºC.

Yield: 33%. ^{1}H-NMR in $CDCl_3$ (400 MHz) δ (ppm): 11.25 (d, *J* = 8.2, 1H, NH), 9.40 (s, 1H, NH), 7.86 (d, *J* = 15.2, 1H, H_{alkene}), 7.44 (d, *J* = 5.3, 1H, H_{aryl}), 7.32 (dd, *J* = 3.8, 1.1, 1H, H_{aryl}), 7.07 (dd, *J* = 5.1, 3.6, 1H, H_{aryl}), 6.37 (d, *J* = 15.2, 1H, H_{alkene}), 4.33 (m, 1H, $H_{cyclohexyl}$), 2.12 – 2.05 (m, 2H, $H_{cyclohexyl}$), 1.75 – 1.23 (m, 8H, $H_{cyclohexyl}$). ^{13}C-NMR in $CDCl_3$ (100 MHz) δ (ppm): 178.23 (C=Se), 165.77 (C=O), 139.24 (C_{aryl}), 138.73 (C_{alkene}), 132.37 (C_{aryl}), 129.97 (C_{aryl}), 128.54 (C_{aryl}), 116.97 (C_{alkene}), 57.21 ($C_{cyclohexyl}$), 31.59 ($C_{cyclohexyl}$), 25.45 ($C_{cyclohexyl}$), 24.34 ($C_{cyclohexyl}$). ^{77}Se-NMR in $CDCl_3$ (76 MHz) δ (ppm): 311.05. Elemental analysis for $C_{14}H_{18}N_2OSSe$ calculated/found (percent): C, 49.26/49.36; H, 5.32/5.48; N, 8.21/8.15.

(*E*)-3-(Benzo[*d*][1,3]dioxol-5-yl)-*N*-(cyclohexylcarbamoselenoyl)acrylamide (14). From 3,4-(methylenedioxy)cinnamic acid and cyclohexylamine. Appearance: orange solid. Melting point: 149-151 ºC. Yield: 30%. ^{1}H-NMR in $CDCl_3$ (400 MHz) δ (ppm): 11.28 (d, *J* = 8.3, 1H, NH), 9.32 (s, 1H, NH), 7.66 (d, *J* = 15.4, 1H, H_{alkene}), 7.05 – 7.01 (m, 2H, H_{aryl}), 6.83 (d, *J* = 8.5, 1H, H_{aryl}), 6.37 (d, *J* = 15.4, 1H, H_{alkene}), 6.03 (s, 2H, CH_2), 4.33 (dt, *J* = 9.3, 4.6, 1H, $H_{cyclohexyl}$), 2.11 – 2.05 (m, 2H, $H_{cyclohexyl}$), 1.75 – 1.23 (m, 8H, $H_{cyclohexyl}$). ^{13}C-NMR in $CDCl_3$ (100 MHz) δ (ppm): 178.19 (C=Se), 166.26 (C=O), 150.50 (C_{aryl}), 148.64 (C_{aryl}), 146.18 (C_{alkene}), 128.41 (C_{aryl}), 125.56 (C_{aryl}), 116.26 (C_{alkene}), 108.80 (C_{aryl}), 106.87 (C_{aryl}), 101.87 (CH_2), 57.16 ($C_{cyclohexyl}$), 31.61 ($C_{cyclohexyl}$), 25.46 ($C_{cyclohexyl}$), 24.31 ($C_{cyclohexyl}$). ^{77}Se-NMR in $CDCl_3$ (76 MHz) δ (ppm): 302.79. Elemental analysis for $C_{17}H_{20}N_2O_3Se$ calculated/found (percent): C, 53.83/54.02; H, 5.31/5.69; N, 7.39/7.35.

***N*-(Isopropylcarbamoselenoyl) benzamide (15).** From benzoyl chloride and isopropylamine. Appearance: brown solid. Melting point: 125-127 ºC. Yield: 10%. ^{1}H-NMR in DMSO-d_6 (400 MHz) δ (ppm): 11.46 (s, 1H, NH), 11.29 (d, *J* = 8.0, 1H, NH), 7.91 (dd, *J* = 8.4, 1.3, 2H, H_{aryl}), 7.64 (m, 1H, H_{aryl}), 7.51 (ddd, *J* = 8.1, 6.7, 1.2, 2H, H_{aryl}), 4.51 (dt, *J* = 8.0, 6.6, 1H, $H_{isopropyl}$), 1.29 (d, *J* = 6.6, 6H, $H_{isopropyl}$). ^{13}C-NMR in DMSO-d_6 (100 MHz) δ (ppm): 178.84 (C=Se), 168.27 (C=O), 133.09 (C_{aryl}), 131.97 (C_{aryl}), 128.62 (C_{aryl}), 128.41 (C_{aryl}), 49.93 ($C_{isopropyl}$), 21.05 ($C_{isopropyl}$). ^{77}Se-NMR in DMSO-d_6 (76 MHz) δ (ppm): 342.44. Elemental analysis for $C_{11}H_{14}N_2OSe$ calculated/found (percent): C, 49.08/48.94; H, 5.24/5.13; N, 10.41/10.56.

2-((Isopropylcarbamoselenoyl)carbamoyl)phenyl acetate (16). From *O*-acetylsalicyloyl chloride and isopropylamine. Appearance: red solid. Melting point: 75-77 ºC. Yield: 9%. ^{1}H-NMR in $CDCl_3$ (400 MHz) δ (ppm): 11.00 (s, 1H, NH), 10.08 (s, 1H, NH), 8.02 (dd, *J* = 7.9, 1.7, 1H, H_{aryl}), 7.61 (ddd, *J* = 8.2, 7.4, 1.8, 1H, H_{aryl}), 7.39 (td, *J* = 7.6, 1.1, 1H, H_{aryl}), 7.25 (dd, *J* = 8.3, 1.1, 1H, H_{aryl}), 4.58 (m, 1H, $H_{isopropyl}$), 2.59 (s, 3H, CH_3), 1.37 (d, *J* = 6.5, 6H, $H_{isopropyl}$). ^{13}C-NMR in $CDCl_3$ (100 MHz) δ (ppm): 178.53 (C=Se), 168.39 (C=$O_{acetoxi}$), 164.06 (C=O), 148.50 (C_{aryl}), 134.56 (C_{aryl}), 131.61 (C_{aryl}), 126.87 (C_{aryl}), 124.01 (C_{aryl}), 123.88 (C_{aryl}), 51.24 ($C_{isopropyl}$), 21.75 ($C_{isopropyl}$), 21.59 (CH_3). ^{77}Se-NMR in $CDCl_3$ (76 MHz) δ (ppm): 341.81. Elemental analysis for $C_{13}H_{16}N_2O_3Se$ calculated/found (percent): C, 47.71/47.83; H, 4.93/5.06; N, 8.56/8.68.

***N*-(Isopropylcarbamoselenoyl)thiophene-2-carboxamide (17).** From 2-thiophenecarbonyl chloride and isopropylamine. Appearance: brown solid. Melting point: 120-122 ºC. Yield: 16%. ^{1}H-NMR in DMSO-d_6 (400 MHz) δ (ppm): 11.52 (s, 1H, NH), 11.15 (d, *J* = 7.9, 1H, NH), 8.35 (dd, *J* = 3.9, 1.1, 1H, H_{aryl}), 8.02 (dd, *J* = 5.0, 1.1, 1H, H_{aryl}), 7.22 (dd, *J* = 5.0, 3.9, 1H, H_{aryl}), 4.78 – 4.22 (m, 1H, $H_{isopropyl}$), 1.27 (d, *J* = 6.5, 6H, $H_{isopropyl}$). ^{13}C-NMR in DMSO-d_6 (100 MHz) δ (ppm): 178.37 (C=Se), 162.03 (C=O), 136.44 (C_{aryl}), 135.31 (C_{aryl}), 132.75 (C_{aryl}), 128.75 (C_{aryl}), 49.94 (s, 1C, $C_{isopropyl}$), 21.01 (s, 2C, $C_{isopropyl}$). ^{77}Se-NMR in DMSO-d_6 (76 MHz) δ (ppm): 343.84. Elemental analysis for $C_9H_{12}N_2OSSe$ calculated/found (percent): C, 39.27/39.33; H, 4.39/4.56; N, 10.18/10.09.

***N*-(Isopropylcarbamoselenoyl)cinnamamide (18).** From cinnamoyl chloride and isopropylamine. Appearance: yellow solid. Melting point: 147-149 ºC. Yield: 38%. ^{1}H-NMR in DMSO-d_6 (400 MHz) δ (ppm): 11.49 (s, 1H, NH), 11.28 (d, *J* = 7.9, 1H, NH), 7.70 (d, *J* = 15.7, 1H, H_{alkene}), 7.60 (dd, *J* = 6.8, 2.9, 2H, H_{aryl}), 7.46 (dd, *J* = 4.8, 1.9, 3H, H_{aryl}), 7.06 (d, *J* = 15.8, 1H, H_{alkene}), 4.44 (dt, *J* = 8.0, 6.6, 1H, $H_{isopropylamine}$), 1.27 (d, *J* = 6.5, 6H, $H_{isopropylamine}$). ^{13}C-NMR in DMSO-d_6 (100

MHz) δ (ppm): 178.58 (C=Se), 166.23 (C=O), 144.42 (C_{alkene}), 134.10 (C_{alkene}), 130.78 (C_{aryl}), 129.15 (C_{aryl}), 128.23 (C_{aryl}), 119.89 (C_{alkene}), 49.63 ($C_{isopropyl}$), 21.14 ($C_{isopropyl}$). ^{77}Se-NMR in DMSO-d_6 (76 MHz) δ (ppm): 329.91. Elemental analysis for $C_{13}H_{16}N_2OSe$ calculated/found (percent): C, 52.88/52.73; H, 5.46/5.51; N, 9.49/9.37.

***N*-(Isopropylcarbamoselenoyl)benzo[*d*][1,3]dioxole-5-carboxamide (19).** From piperonylic acid and isopropylamine. Appearance: brown solid. Melting point: 128-130 ºC. Yield: 22%. ^{1}H-NMR in $CDCl_3$ (400 MHz) δ (ppm): 11.08 (s, 1H, NH), 9.12 (s, 1H, NH), 7.37 (dd, *J* = 8.2, 1.9, 1H, H_{aryl}), 7.28 (d, *J* = 1.9, 1H, H_{aryl}), 6.87 (d, *J* = 8.2, 1H, H_{aryl}), 6.07 (s, 2H, CH_2), 4.58 (dt, *J* = 8.2, 6.6, 1H, $H_{isopropyl}$), 1.35 (d, *J* = 6.6, 6H, $H_{isopropyl}$). ^{13}C-NMR in $CDCl_3$ (100 MHz) δ (ppm): 178.69 (C=Se), 166.08 (C=O), 152.48 (C_{aryl}), 148.74 (C_{aryl}), 125.43 (C_{aryl}), 123.06 (C_{aryl}), 108.58 (C_{aryl}), 107.83 (C_{aryl}), 102.42 (CH_2), 51.26 ($C_{isopropyl}$), 21.74 ($C_{isopropyl}$). ^{77}Se-NMR in $CDCl_3$ (76 MHz) δ (ppm): 322.77. Elemental analysis for $C_{12}H_{14}N_2O_3Se$ calculated/found (percent): C, 46.02/45.90; H, 4.51/4.58; N, 8.94/9.10.

(E)-*N*-(Isopropylcarbamoselenoyl)-3-(thiophen-2-yl)acrylamide (20). From 3-(2-thienyl)acrylic acid and isopropylamine. Appearance: yellow solid. Melting point: 148-149 ºC. Yield: 15%. ^{1}H-NMR in DMSO-d_6 (400 MHz) δ (ppm): 11.46 (s, 1H, NH), 11.25 (d, *J* = 8.0, 1H, NH), 7.85 (d, *J* = 15.5, 1H, H_{alkene}), 7.75 (d, *J* = 5.1, 1H, H_{aryl}), 7.51 (d, *J* = 3.51, 1H, H_{aryl}), 7.16 (dd, *J* = 5.0, 3.6, 1H, H_{aryl}), 6.80 (d, *J* = 15.4, 1H, H_{alkene}), 4.43 (dt, *J* = 8.0, 6.6, 1H, $H_{isopropyl}$), 1.26 (d, *J* = 6.6, 6H, $H_{isopropyl}$). ^{13}C-NMR in DMSO-d_6 (100 MHz) δ (ppm): 178.52 (C=Se), 166.08 (C=O), 139.31 (C_{aryl}), 137.24 (C_{alkene}), 132.93 (C_{alkene}), 130.37 (C_{aryl}), 128.72 (C_{aryl}), 118.16 (C_{alkene}), 49.60 ($C_{isopropyl}$), 21.15 ($C_{isopropyl}$). ^{77}Se-NMR in DMSO-d_6 (76 MHz) δ (ppm): 329.07. Elemental analysis for $C_{11}H_{14}N_2OSSe$ calculated/found (percent): C, 43.85/43.80; H, 4.68/4.44; N, 9.30/9.62.

(*E*)-3-(Benzo[*d*][1,3]dioxol-5-yl)-*N*-(isopropylcarbamoselenoyl)acrylamide (21). From 3,4-(methylenedioxy)cinnamic acid and isopropylamine. Appearance: orange solid. Melting point: 172-174 ºC. Yield: 10%. ^{1}H-NMR in $CDCl_3$ (400 MHz) δ (ppm): 11.15 (d, *J* = 8.0, 1H, NH), 9.24 (s, 1H, NH), 7.66 (d, *J* = 15.4, 1H, H_{alkene}), 7.07 – 7.00 (m, 2H, H_{aryl}), 6.83 (d, *J* = 8.4, 1H, H_{aryl}), 6.33 (d, *J* = 15.4, 1H, H_{alkene}), 6.03 (s, 2H, CH_2), 4.83 – 4.37 (m, 1H, $H_{isopropyl}$), 1.35 (d, *J* = 6.6, 6H, $H_{isopropyl}$). ^{13}C-NMR in $CDCl_3$ (100 MHz) δ (ppm): 178.59 (C=Se), 166.04 (C=O), 150.59 (C_{aryl}), 148.69 (C_{aryl}), 146.34 (C_{alkene}), 128.33 (C_{aryl}), 125.62 (C_{aryl}), 116.11 (C_{alkene}), 108.86 (C_{aryl}), 106.83 (C_{aryl}), 101.92 (CH_2), 51.03 ($C_{isopropyl}$), 21.81 ($C_{isopropyl}$). ^{77}Se-NMR in $CDCl_3$ (76 MHz) δ (ppm): 312.38. Elemental analysis for $C_{14}H_{16}N_2O_3Se$ calculated/found (percent): C, 49.56/49.67; H, 4.75/4.59; N, 8.26/8.48.

2-Hydroxy-*N*-(isopropylcarbamoselenoyl)benzamide (22). From *O*-acetylsalicyloyl chloride and isopropylamine. Appearance: yellow solid. Melting point: 162-164 º C. Yield: 9%. ^{1}H-NMR in $CDCl_3$ (400 MHz) δ (ppm): 11.00 (s, 1H, OH), 10.50 (s, 1H, NH), 9.95 (s, 1H, NH), 7.65 (dd, *J* = 8.1, 1.6, 1H, H_{aryl}), 7.50 (ddd, *J* = 8.7, 7.2, 1.6, 1H, H_{aryl}), 7.12 (dd, *J* = 8.5, 1.1, 1H, H_{aryl}), 6.98 (ddd, *J* = 7.6, 7.0, 1.1, 1H, H_{aryl}), 4.60 (dt, *J* = 8.2, 6.6, 1H, $H_{isopropyl}$), 1.39 (d, *J* = 6.5, 6H, $H_{isopropyl}$). ^{13}C-NMR in $CDCl_3$ (100 MHz) δ (ppm): 178.17 (C=Se), 167.99 (C=O), 160.12 (C_{aryl}), 136.35 (C_{aryl}), 128.42 (C_{aryl}), 120.50 (C_{aryl}), 118.79 (C_{aryl}), 113.95 (C_{aryl}), 51.47 ($C_{isopropyl}$), 21.79 ($C_{isopropyl}$). ^{77}Se-NMR in $CDCl_3$ (76 MHz) δ (ppm): 334.36. Elemental analysis for $C_{11}H_{14}N_2O_2Se$ calculated/found (percent): C, 46.32/46.56; H, 4.95/4.88; N, 9.82/9.71.
